# Diffusive and Electro-osmotic Swelling of Neutral and Ion-Containing Poly(ethylene glycol) Hydrogels

**DOI:** 10.1021/acsomega.5c00865

**Published:** 2025-08-19

**Authors:** Shefik D. Bowen, Lexy D. Herrera, Daniel T. Hallinan

**Affiliations:** 86485 Florida A&M University-Florida State University College of Engineering, 2525 Pottsdamer Street, Tallahassee, Florida 32310-6046, United States

## Abstract

This study presents a comprehensive analysis of the swelling behavior of poly­(ethylene glycol) (PEG)-based hydrogels of different molecular weights under various conditions. The rheological response and swelling kinetics of PEG hydrogels with molecular weight between cross-links ranging from 700 to 10 000 g/mol reveal the connection between architecture and material properties that are important for soft actuators. In addition to providing insight into the network structure and cross-linking density, rheological measurements find that the shear moduli of the networks increase with the degree of water swelling. Furthermore, the moving boundary of the gels during swelling, which is captured using a numerical model, is found to result in apparent super Case II water transport behavior. Finally, the application of an electric field in the presence of a salt solution is found to increase the rate of swelling by an order of magnitude when electro-osmosis is in the same direction as diffusion or decrease the swelling rate by an even greater amount when directions are opposed. The integration of experimental data with numerical models contributes to a deeper understanding of hydrogel behavior, guiding the design of PEG-based systems for biomedical or soft robotics applications. PEG hydrogels with dense network structures can do useful work and are promising for applications such as twisted and coiled actuators due to their ability to maintain a high modulus while radially swelling.

## Graphical abstract

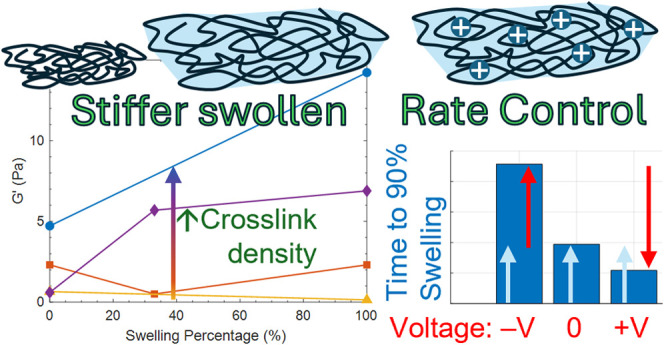

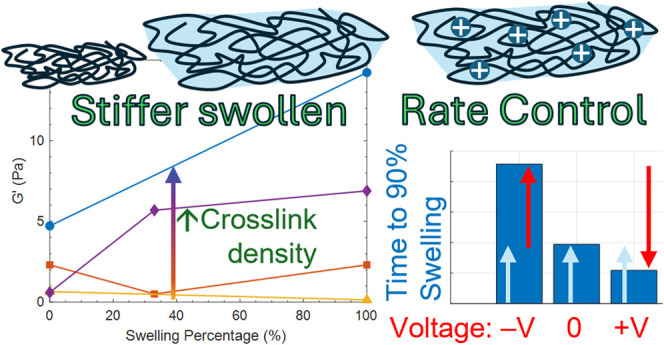

## Introduction

Artificial muscles are a critical component of future robotics, autonomous vehicles, and advanced biomimetic electronics and require materials that mimic biological actuation. Such materials must be soft, nonmetallic for stealth, tough, biocompatible, lightweight, and self-healing.^1^ Rapid, powerful actuation in the aforementioned applications is best suited to electric control, which requires the material to additionally be electroactive or electro-responsive. Charge-containing soft materials, such as polymers, networks, and hydrogels, exhibit these properties and are a promising class of materials to study.

Large strains are possible in twisted and coiled actuators (TCAs) that can be formed from polymer-containing fibers and filaments.^2,3^ The response of TCAs is a fundamental phenomenon captured by elastic rod theory, whereby radial swelling results in much larger axial contraction.^3–5^ Radial swelling of a TCA has been induced most commonly with thermal expansion^6–15^ but is also achieved via solvent uptake and/or pH changes.^16–21^ Network cross-link density affects two important aspects of TCA design: the amount of radial swelling, which controls TCA strain, and the elastic modulus, which affects the amount of load that a TCA can lift. As shown in eq 1, both fiber radius, *R*, and modulus, *E*, affect the length of a coil, *l* cos β, for a given amount of twist, *n*, and applied load, *F*. This expression for TCA fiber coil length based on untwisted fiber length, *l*, and coil angle, β, is derived from elastic rod theory for a fiber of circular cross-section composed of an ideal elastomer.^4,22^

$$l\cos\beta =l\left|nR^{2}\sqrt{\frac{\pi E}{F}}-5\right|$$ 1

The fact that network modulus and degree of swelling depend on cross-link density motivates the detailed exploration of the effect of cross-link density on gel actuation in this study.^23^

This work also explores the possibility of accelerating solvent uptake using electroosmosis, which we previously showed is possible using a continuum transport/swelling model (to predict rate) combined with a thermodynamic model based on Flory–Rehner Theory (to predict equilibrium swelling).^22^ The transport/swelling model, as shown in eq 2

2a $$\frac{d\phi_{s}}{dt}=D_{12}\left[\nabla^{2}\phi_{s}+\frac{z_{1}F}{R_{gas}T}\nabla \cdot \left(\phi_{s}\nabla \Phi \right)\right]$$

2b $$\nabla \left(j,k\right)={\left(1-\phi_{s}\right)}^{1/3}\nabla \left(j,0\right)$$

predicts the swelling rate by scaling the discrete “differential” pieces of volume of polymer based on the local volume fraction of solvent (ϕ_s_) as it changes in time due to diffusion (first term of eq 2a RHS) and ion solvation shell migration (second term of eq 2a RHS). In this equation, *t* is time, *D*_12_ is the mutual diffusion coefficient, *z* is the solvation number of the ion, F is Faraday’s constant, *R*_gas_ is the universal gas constant, *T* is the temperature, Φ is the electric potential, and ∇ represents the gradient operator. The model was able to calculate swelling rate with and without applied electric field but has not been validated experimentally.^22^

From the perspective of green chemistry, cost, and environmental considerations, it is desirable to use aqueous solutions. Hydrogels play a pivotal role in this regard due to their intrinsic capacity to absorb substantial water quantities. Their swelling behavior, hitherto leveraged in traditional applications like superabsorbents, is now being repurposed for innovative uses in flexible electronics and smart actuators.^24–26^ However, the large volume increase that typifies hydrogels can compromise mechanical performance, which new applications often cannot afford.

The rate of hydrogel swelling is significantly influenced by the polymer network structure and physicochemical properties, with fast swelling rates being crucial for applications such as drug delivery and soft robotics. Applying an electric field, as demonstrated for bending actuation by Eun-Ju Ha et al.,^27^ can accelerate swelling in polyelectrolyte hydrogels from hours to minutes by enhancing water and ion movement within the gel. Furthermore, adjustments in cross-link density and hydrophilic group concentration can effectively tailor the swelling kinetics of hydrogels. Networks with lower cross-link density, due to higher molecular weight cross-linker and/or larger water content during gelation, have a larger volumetric swelling ratio and a faster swelling rate.^28^ The swelling rate can also be influenced by the concentration of hydrophilic groups such as hydroxyl (−OH) or carboxyl (−COOH) groups, which enhance water uptake through hydrogen bonding.^29^ Additionally, incorporating ionizable groups like −COOH or −SO_3_H can further accelerate swelling by generating osmotic pressure as these groups dissociate in response to changes in hydration and/or pH.^30^ This ability to modulate both cross-link density and hydrophilic/ionizable group content provides a versatile chemical approach to control hydrogel swelling behavior. The use of electro-osmotic mechanisms offers a dynamic method to modify hydrogel dimensions and properties, crucial for developing responsive biomimetic devices. Murdan highlighted how electric fields can be used to control drug release rates from polyelectrolyte gels in a dynamic fashion, providing essential adaptability for various biomedical applications,^31^ but fundamental investigation of the direct impact of electro-osmosis on swelling has received less attention.

This report delves into the electro-osmotic phenomenon as a means to control swelling by electrically drawing solvent molecules into the polymer matrix, thus inducing swelling rate increase. The intent is to compare model predictions to actual hydrogel response to develop a quantitative understanding to predict the extent that voltage can be used to accelerate/decelerate the diffusive swelling process. This will facilitate the development of responsive systems that can be used for precise actuation in artificial muscle applications.

## Materials and Methods

### Synthesis and Preparation of Gels

Hydrogels were synthesized from poly­(ethylene glycol diacrylate) (PEGDA) of various molecular weights, as shown in Table 1. The molecular weights were chosen based on their ease of dissolution in water. PEGDAs of low molecular weight, *M*_*n*_ = 700 g/mol (PEG700) and *M*_*n*_ = 3400 g/mol, were supplied by Advanced Biomatrix. Higher molecular weight poly­(ethylene glycol) (PEG) was acquired with hydroxyl end groups and modified. *M*_*n*_ = 4000 g/mol (PEG4k) was supplied by Millipore Sigma; 8000 g/mol (PEG8k) was supplied by Grainger; and 10 000 g/mol (PEG10k) was supplied by Fluka. PEG was dissolved in dichloromethane (Sigma-Aldrich) and functionalized to PEGDA using acryloyl chloride (Sigma-Aldrich) in the presence of triethylamine (Sigma-Aldrich) as described elsewhere.^32,33^ The synthesized PEGDA was then purified by precipitation in anhydrous diethyl ether, ACS grade stabilized with 5 ppm of BHT (99.9%) (Oakwood Chemicals).

**Table 1:** PEG Sample Designation (SD) Based on Number-Average Molecular Weight Reported by the Manufacturer, Where Samples Are Named PEG­(SD), and Number of Chemical Monomers Per Chain As Determined by NMR Analysis

SD	*M*_*n*_ ^a^ (g/mol)	*N* ^b^
PEGDA700	700	10
PEG4k	4,000	96
PEG8k	8,000	213
PEG10k	10,000	275

a PEG or PEGDA molar mass reported by the manufacturer. Note that PEGDA700 was received as PEGDA.

b Degree of polymerization (number of chemical monomers) from NMR end-group analysis after end-group functionalization to form PEGDA or as received in the case of PEGDA700.

To form hydrogels, PEGDA was first dissolved in deionized (DI) water at 15 wt % for 700 g/mol PEGDA and at 10 wt % for all other samples. To each PEGDA solution was added Irgacure 2959 (Advanced Biomatrix) at 1 wt % following the procedures described in refs 32 and 33. In order to form a robust hydrogel with 700 g/mol PEGDA, it was necessary to use a higher concentration (15% polymer) than that reported in the literature (10% polymer). PEGDA gel precursor solution was cross-linked by curing for 13 min under 365 nm UV light at $1.5\frac{W}{{\mathrm{cm}}^{2}}$ using a 36 W Melody Susie nail hardener. Elasticity was confirmed by gently touching the sample after the exposure time ended. As-synthesized hydrogels were disks of 4 ± 1 mm thickness and 5.5 cm diameter.

Hydrogels were placed over a 40 °C hot plate open to ambient conditions for 30 min to remove bulk water. Samples were then completely dried at 40 °C under vacuum overnight. After complete drying, dry sample mass (*m*_dry_) and thickness (*L*_dry_) were measured. Next, a swelling experiment was conducted by exposing the sample to DI water or an aqueous electrolyte solution of 0.5 M NaCl. Samples were stored under ambient conditions between experiments. Data sets reported in figures are from representative samples that exhibited median behavior of multiple experiments, whereas values reported in tables are from analysis of experiments conducted on five or more different samples. The reported uncertainties are one standard deviation.

### Characterization

For nuclear magnetic resonance (NMR), PEGDA samples were dissolved in deuterated chloroform (Sigma-Aldrich) at a concentration of 10 mg/mL. ^1^H NMR spectra were acquired using Bruker 400, 500, or 600 MHz NMR spectrometers, collecting 32 scans per spectrum.

Fourier transform infrared spectroscopy with attenuated total reflectance (FTIR-ATR) was also used to characterize the hydrogel synthesis. Spectra were collected using a PerkinElmer Frontier FTIR instrument with a Specac Golden Gate single-reflection diamond ATR accessory and a DTGS detector. After collecting a background, dry PEG, PEGDA, and hydrogel samples were placed directly on the ATR crystal and spectra collected with 16 scans at a resolution of 4 cm^–1^.

### Rheology

Moduli of PEGDA700, 4k, 8k, and 10k gels were examined in this study using an Anton Paar MC-304e rheometer to conduct small amplitude oscillatory shear rheology with 8 or 25 mm parallel plate geometry. Strain amplitude sweeps between 0.001% and 1% were conducted to determine the linear viscoelastic regime, and frequency sweeps were conducted between 0.01 and 100 Hz. Temperature sweeps were conducted from 70 to 20 °C at a cooling rate of 1 °C/min with a frequency of 1 Hz while maintaining 1–2 N of normal force to ensure adhesion. Amorphous moduli were averaged from measurements between 65 and 70 °C, where the strain amplitude was between 0.1% and 0.2%. The storage modulus is an important material property of TCA fibers as it influences maximum load that a TCA can lift.^22,34–36^ The modulus values were used to approximate the cross-link density of each sample, which is also important as it dictates the amount of swelling that can occur. Rheology was also used to monitor the UV curing by measuring the storage and loss moduli at 20 °C and a frequency of 1 Hz and amplitude of 0.01%. The gap was set to 1 mm, and the PEGDA gel precursor solution was injected to fill the gap and form a convex meniscus at the perimeter. Reported results are taken from measurements in which no air bubbles were present.

### Swelling Measurements

The swelling behavior of PEGDA700, 4k, 8k, and 10k gels was measured on a gravimetric basis using an A&D GH-202 balance with 0.01 mg resolution and on a dimensional basis using a Mitutoyo Absolute Drop Indicator C112EX with a custom 25 mm diameter foot drilled with holes for better solvent–sample surface contact. Water mass fraction was determined at equilibrium from dry and swollen hydrogel weights according to the following equation:

3 $$w_{s}=\frac{m_{\mathrm{swollen}}-m_{\mathrm{dry}}}{m_{\mathrm{swollen}}}$$

This was also used to determine the polymer mass fraction, *w*_p_ = 1 – *w*_s_ = *m*_dry_/*m*_swollen_.

Time resolved measurements of hydrogel thicknesses were recorded using the experimental setup in Figure S1 during swelling from the dry to the swollen state by exposing the dry hydrogel to DI water. A Mitutoyo RS-232 to USB adapter was used to connect the drop indicator to a computer where digital thickness was recorded in Measurlink. After swelling to equilibrium, the wet sample thickness (*L*_swollen_) and mass (*m*_swollen_) were recorded. The water volume fraction was calculated assuming isotropic swelling, which is a good assumption for cross-linked networks,^23,37^ based on the following equation:

4 $$\phi_{s}\left(L\right)=\frac{L_{\mathrm{swollen}}^{3}-L_{\mathrm{dry}}^{3}}{L_{\mathrm{swollen}}^{3}}$$

This was also used to determine polymer volume fraction ϕ_p_(*L*) = 1 – ϕ_s_(*L*) = *L*_dry_^3^/*L*_swollen_^3^.

### Voltage-Controlled Swelling

Voltage was applied during swelling experiments using a Biologic SP-150 Potentiostat. For these experiments, the sample was placed between nickel mesh electrodes of 25 mm diameter with a tab for alligator clip connection. Swelling solution was supplied below the bottom electrode via filter paper that wicked the solution from a separate reservoir. The hydrogels were subjected to an applied potential ranging in magnitude from 0 to 0.35 V to induce electro-osmotic migration.^23,38^ The transient swelling behavior was monitored by using the same Mitutoyo drop indicator setup.

In these experiments, a PEGDA4k gel sample was used sequentially for negative, neutral, and positive swelling under an applied EMF in a 0.5 M aqueous NaCl solution. After each experiment, the sample was dried under a vacuum at 40 °C and allowed to rest before the next swelling experiment. To prepare for the neutral swelling experiment, the sample was first swollen in pure DI water, without NaCl solution, to rinse out any free ions, and then dried. This ensured that the subsequent swelling experiment under neutral conditions was free of residual ions from previous experiments.

## Results and Discussion

### Chemical Characterization

In Figure 1, the NMR spectra of PEGDA samples are shown, normalized so that the most intense peak, associated with the methylene protons of the backbone repeat unit, has a maximum intensity value of one. The chemical shifts observed between 3.5 and 6.5 ppm are consistent with those of PEGDA across all samples. Characteristic PEGDA peaks are noted in the figure using lowercase letters ‘a’ through ‘f’ that represent protons designated in the chemical structure shown in Table 2. Each spectrum displayed in the insets is normalized by its respective maximum acrylate peak intensity near 5.83 ppm, assigning a value of 2 to represent the number of hydrogens associated with this peak (1 hydrogen per acrylate chain end with 2 chain ends per molecule). All three prominent peaks at approximately 5.83, 6.12, and 6.41 ppm are indicative of the acrylate groups, each containing 3 distinct hydrogens (see Table 2), confirming functionalization. Estimates of the ^1^H NMR peak locations of PEG are also reported in Table 2. As shown in Figures S2 and S3, there is no signal from the hydroxyl protons in the PEGDA samples, indicating that complete acrylate functionalization occurred. The resulting peak areas at the backbone proton chemical shift (3.65–3.68 ppm) represent the proportional number of ^1^H in the ethylene oxide backbone, where 4 H atoms are present in each chemical repeat unit. End-group analysis was performed as follows to calculate the degree of polymerization or average number of chemical monomers per chain, *N*:

5 $$N=\frac{H_{bb}}{H_{eg_{5.83+6.12}}}$$

The ratios of backbone peak area at 3.52 ppm, *H*_bb_, and end-group peak areas at 5.83 and 6.12 ppm, *H*_eg_, were scaled by the number of protons associated with each peak. These values are reported in Table 1 and show that *N* increases with PEG *M*_*n*_. The values of *N* are somewhat larger than expected based on manufacturer reported *M*_*n*_ with exception for the purchased PEGDA700, which is most likely due to chain extension caused by PEGDA chain propagation.^39^

**Figure 1:**
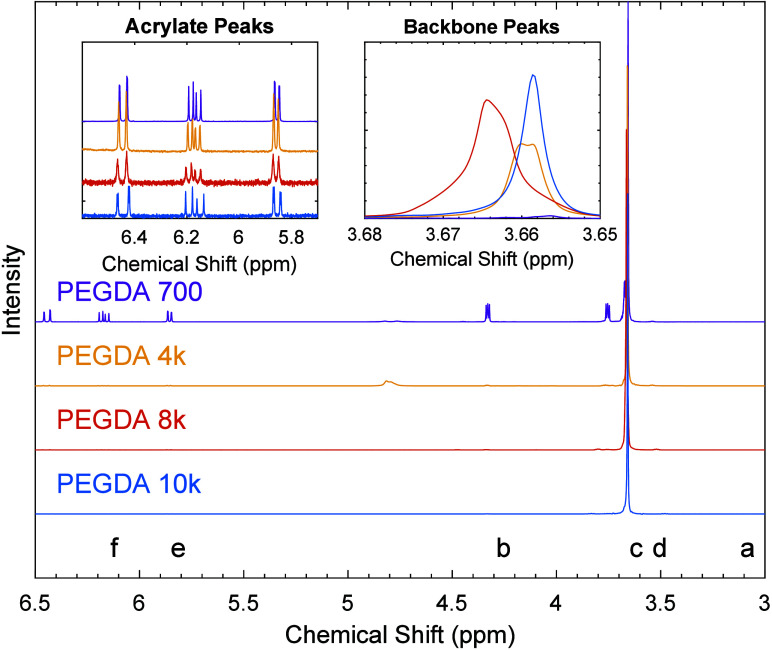
Offset NMR spectra of PEGDA samples dissolved in d-chloroform with molecular weights denoted in the figure. In insets, spectra are normalized by the intensity at 5.85 ppm, which corresponds to the acrylate end-group proton labeled ‘e’ in Table 2. The characteristic peaks of PEGDA at approximately 5.85, 6.15, and 6.42 ppm indicate the presence of acrylate groups, confirming successful functionalization.

**Table 2:**
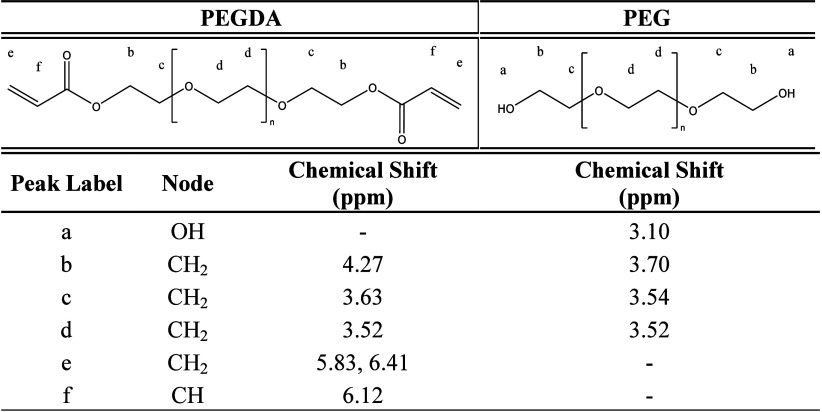
PEGDA and PEG Chemical Structures with Distinct Protons Labeled and ^1^H NMR Chemical Shift Estimations from PerkinElmer ChemDraw

FTIR-ATR spectra are presented in Figures 2 and 3. In Figure 2, the spectra of PEG8k, PEGDA8k, and PEGDA8k Gel are shown with insets focusing on specific regions of interest; refer to Figures S4–S11 for FTIR-ATR spectra of all polymers in this study as well as a PEGDA8k 20% gel that was synthesized but not studied further due to having poor properties. The peaks corresponding to C–H stretching (2500 to 3000 cm^–1^) are associated with the PEG repeat unit and do not change upon end-group functionalization, whereas C–O stretching (1700 to 1750 cm^–1^) corresponds to acrylate functionality that is not present in PEG but is apparent in PEGDA and in PEGDA Gel. The broadening of the acrylate C–O absorbance in the gel is due to the water chemical environment, e.g., hydrogen bonding that is known to cause broadening of FTIR peaks. Figure 3 provides a detailed comparison of the PEGDA samples with different molecular weights. In panel (a), the spectra exhibit distinct peaks in the highlighted regions. Panel (b) shows spectra normalized to the C–O peak at 1724 cm^–1^, thus normalizing absorbance by moles of acrylate end groups in the sample. Panel (c) reveals the relative changes in C–H peak absorbance postnormalization, thus showing the moles of PEG repeat unit per mole of acrylate end group. The integrated C–H peak area versus molecular weight is displayed in panel (d) and confirms the proportional relationship between molecular weight and C–H stretching peak area normalized to C–O stretching. Thus, FTIR further verifies that PEG to PEGDA functionalization occurred.

**Figure 2:**
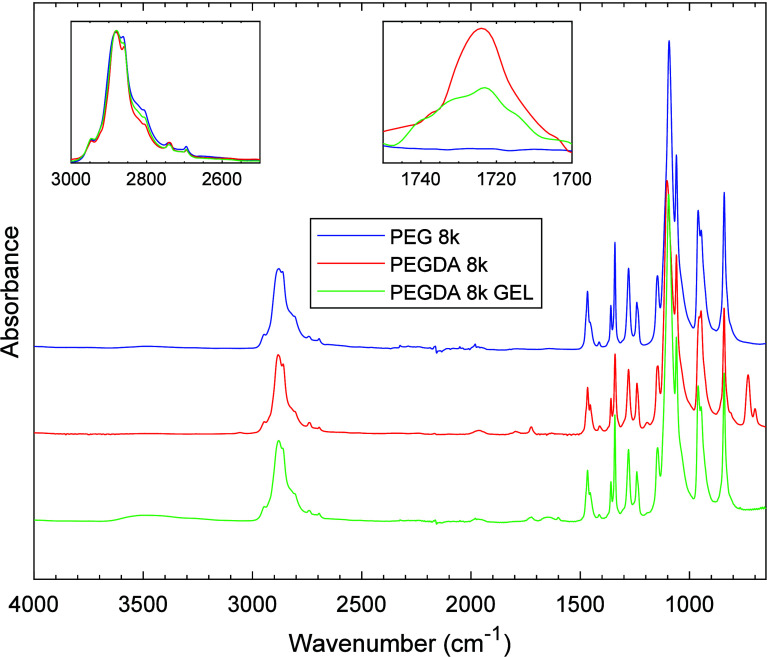
Comparison of FTIR-ATR spectra for 8000 g/mol PEG, PEGDA, and PEGDA gel, offset for clarity. Insets are zoomed in to the respective ranges, CH stretching (3000–2500 cm^–1^) and CO stretching (1750–1700 cm^–1^), and normalized by the maximum absorbance in the CH stretching region.

**Figure 3:**
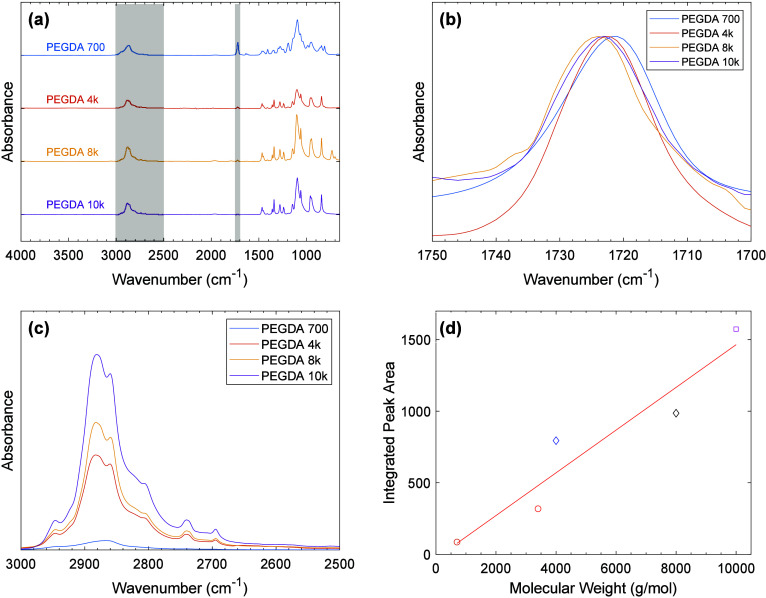
(a) FTIR-ATR scans of 4 molecular weights of PEGDA ranging from 650 to 4000 cm^–1^. Shaded ranges highlight peaks of interest: C–H bond stretching appearing in the range 2500 to 3000 cm^–1^ and C–O stretching appearing in the range from 1700 to 1750 cm^–1^. (b) All spectra are normalized to the C–O peak at 1724 cm^–1^. (c) Resulting relative absorbance of C–H peaks after normalization by acrylate CO stretching. (d) Integrated normalized C–H stretching peak area of PEGDA from 2500 to 3000 cm^–1^ vs number-average molecular weight (*M*_*n*_).

### Network Modulus

Small-amplitude oscillatory shear rheology was used to understand the shear behavior of the PEGDA gels. Figure 4 presents a comprehensive analysis of the temperature-dependent rheological properties of PEGDA hydrogels with varying molecular weights and swelling conditions. The storage modulus of PEGDA was measured as a function of UV exposure time and is shown in Figure 4(a). A sudden increase in storage modulus occurs upon gelation, and the modulus rapidly reaches a plateau value. PEGDA gels exhibit solid-like behavior indicated by the storage modulus being greater than the loss modulus at the plateau (see Figures S12–S15). PEGDA700 gel exhibits the largest storage modulus, as expected based on it being the tightest network. With increasing molecular weight between cross-links, the plateau modulus of the gels generally decreases. However, the PEGDA10k gel exhibits a larger plateau modulus than PEGDA8k gel, likely due to the greater contribution of entanglements with increasing molecular weight. The entanglement molecular weight of PEG is reported to be *M*_*e*_ = 2000 g/mol.^23^ Additional details of rheology results can be found in the Supporting Information, Figures S12 and S21 and Tables S1 and S2.

**Figure 4:**
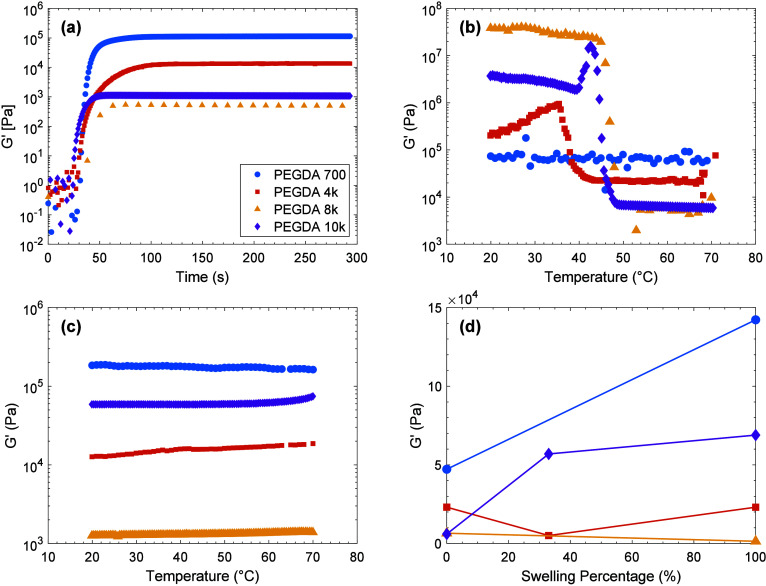
(a) Storage modulus of PEGDA versus UV exposure time from small amplitude oscillatory shear experiments with parallel plates at a frequency of 1 Hz, an amplitude of 0.01%, and a temperature of 20 °C. (b) Temperature sweeps of dry PEGDA gels ramping from 70 to 20 °C at 1 °C/min. The effect of PEG crystallization (causing modulus increase) is pronounced in all samples except PEGDA700 gel. (c) Temperature sweeps of fully water swollen PEGDA gels. PEG cannot crystallize in swollen gels. (d) Amorphous storage modulus as a function of gravimetric swelling percentage, where dry gel mass corresponds to 0% swollen and gel mass fully equilibrated with liquid water is 100% swollen. Intermediate swelling (33%) was achieved by controlling the amount of time a gel was soaked in water; it is shown for PEGDA4k (5 kPa) and PEGDA10k (57 kPA). The solid lines are guides for the eye.

The plateau storage moduli (*G*′) of dry PEGDA gels were measured with temperature sweeps, the results of which are shown in Figure 4(b). In the amorphous state (above 50 °C), the molecular weight dependence of the storage modulus of dry samples is the same as was observed upon gelation. However, the magnitudes of the moduli are considerably different, as shown in Table 3. Interestingly, a significant increase in modulus occurs upon cooling from 50 to 35 °C, in all samples except PEGDA700 gel. This is due to PEG crystallization, which does not occur in PEGDA700 gel due to the melting temperature being below 20 °C.^40,41^

**Table 3:** Hydrogel Storage Moduli from Rheology Measurements at 1 Hz in the Linear Viscoelastic Regime and the Conditions Noted^a^

Sample Name	*G*_cured_^′^ ^a^ (kPa)	*G*_dry_^′^ ^b^ (kPa)	*G*_swollen_^′^ ^c^ (kPa)
PEGDA700 Gel	113.6	47.2	142.3
PEGDA4k Gel	13.6	23.1	23.1
PEGDA8k Gel	0.5	6.5	1.4
PEGDA10k Gel	1.1	6.0	68.9

a Uncertainty based on one standard deviation is less than 2% for the cured modulus and averages less than 20% for the other measurements.

b *G*_cured_^′^ values are the average storage moduli from the last 20 s of gelation at 20 °C.

c *G*_dry_^′^ values are the average storage moduli from 65 to 70 °C of dry samples.

d *G*_swollen_^′^ values are the average storage moduli of 100% swollen samples from 65 to 70 °C.

Temperature sweeps of fully swollen samples are shown in Figure 4(c). The characteristic signature of crystallization is absent due to the presence of water causing crystallite dissolution.^42^ Trends in modulus with molecular weight are similar to gelled and dry, amorphous samples for 700, 4k, and 8k, but values are considerably different, as shown in Table 3. On the other hand, the 10k swollen modulus is an order of magnitude greater than the as-gelled and dry values. We speculate that crystallization causes changes in the network structure and distribution of cross-links that lead to an increased contribution from entanglements.

As shown in Figure 4(d), network swelling by water, for the most part, results in a considerable increase of modulus. This could be due to chain stretching as well as antiplasticization that has been observed to occur when solutes fill polymer free volume.^43–45^ We are not aware of a theoretical treatment of this behavior but note that similar behavior has been observed in other studies of PEGDA gels.^46^ Table 3 summarizes the dry and swollen storage moduli in the amorphous state, which was taken as the average modulus between 65 and 70 °C. The data reveal that the 700 g/mol sample has the highest amorphous modulus, indicating a highly cross-linked and dense network structure. As the molecular weight increases to 4000 and 8000 g/mol, the amorphous modulus decreases significantly, reflecting a reduction in cross-link density and a looser network formation. However, at 10 000 g/mol, in the as-cured and swollen states (solvent present), the amorphous modulus increases again, suggesting that at higher molecular weights the polymer chains become sufficiently entangled, enhancing the network stiffness despite a lower cross-link density. Note that in the absence of solvent (see dry state in Table 3) this is not the case. This seems to indicate that the engagement of entanglements requires that the gel be in a state similar to how it was synthesized and that collapse of the gel by drying it relaxes the engagement of entanglements such that they are not detected in linear viscoelastic, small-amplitude rheological measurements.

Interestingly, the trend in swollen moduli shows a significant increase when water is present at equilibrium quantities, particularly for the 10k sample, which demonstrates a high swollen modulus. This increase in modulus with swelling is indicative of the hydrogel’s ability to maintain its structural integrity and mechanical strength even when fully hydrated, a desirable property for applications in biomedical and soft robotics fields where materials are often required to operate in aqueous environments. These observations align with the principles discussed by Lodge and Flory along with others,^47–51^ which highlight the complex interplay between cross-link density, chain entanglement, and solvent interaction in determining the mechanical properties of polymer networks.

Recent studies have demonstrated that the polymerization of low molecular weight PEGDA leads to a network microstructure that is fundamentally different from that of classical polymeric gels, comprising PEG chains interconnected by multifunctional densely grafted rod-like polyacrylates (PAs) which serve as cross-linkers.^52^ This unique microstructure enables significant deformation capabilities, making PEGDA hydrogels highly suitable for various applications, including tissue engineering and drug delivery.^52^ Based on reference 51, PA contributes significantly to mechanical properties at low molecular weights, with a dominant contribution to network modulus in 700 g/mol gels and approximately 50% contribution to network modulus in 4k gels, but the PA contribution rapidly drops off with increasing PEGDA molecular weight, such that behavior of 8k and 10k hydrogels is predominantly dictated by the PEG network.

### Swelling and Diffusion

Results of the equilibrium gravimetric and thickness-based swelling measurements are reported in Table 4 and Figures S22–S24. The polymer mass fraction in the fully swollen state from gravimetric measurements, *w*_p_, generally decreases with increasing PEGDA molecular weight, indicating less polymer content per volume in the swollen gels, which is due to the looser networks taking up more water, as expected. The amount of gravimetric swelling appears to plateau with the PEGDA8k gel, whose value is not statistically different from the PEGDA10k gel. Mass fractions were converted to volume fractions, assuming volume additivity, using eq 6.

6 $$\phi_{p}\left(w_{p}\right)=\frac{\frac{w_{p}}{\rho_{p}}}{\frac{w_{p}}{\rho_{p}}+\frac{1-w_{p}}{\rho_{w}}}$$

**Table 4:** Equilibrium Polymer Mass and Volume Fractions from Gravimetric Measurements, Swollen Polymer Volume Fractions from Thickness Measurements, and Anisotropy

sample name	*w*_p_	ϕ_p_(*w*_p_) (mass based)^a^	ϕ_p_(*L*) (thickness based)	anisotropy
PEGDA700 gel	0.36 ± 0.17	0.34	0.33 ± 0.15	0.98
PEGDA4k gel	0.13 ± 0.03	0.12	0.34 ± 0.26	1.71
PEGDA8k gel	0.07 ± 0.02	0.06	0.41 ± 0.21	2.52
PEGDA10k gel	0.12 ± 0.07	0.11	0.55 ± 0.20	2.28

a Volume fractions were calculated using the volume additivity and densities of amorphous PEG and water. The densities of PEG and water at 70 °C (where the polymer is fully amorphous) are ρ_p_ = 1.09 g/mL^53^ and ρ_w_ = 0.97915 g/mL, respectively.

The water volume fraction, ϕ_s_ = 1 – ϕ_p_, of PEGDA700 gels from gravimetric measurements is 0.66 and agrees well with literature that reports a value of 0.62.^54^ The thickness-based polymer volume fraction, ϕ_p_(*L*), follows a somewhat similar decreasing trend with an increasing PEGDA molecular weight. However, visual observation during thickness swelling measurements indicated that the hydrogels swelled considerably more in the radial direction than in the thickness direction. This means that isotropic swelling, used to calculate ϕ_p_(*L*), is not a good assumption for these hydrogels under the conditions studied. To quantify the anisotropy of swelling, the swollen polymer volume fraction from gravimetric measurements, ϕ_p_(*w*_p_), was compared to the volume fraction from thickness measurements, ϕ_p_(*L*), as shown in Table 4. Anisotropy is reported as the following ratio that quantifies the amount that radial swelling exceeds swelling in the thickness direction:

7 $$\mathrm{anisotropy}={\left[\frac{\phi_{p}\left(L\right)}{\phi_{p}\left(w_{p}\right)}\right]}^{1/2}=\frac{D_{\mathrm{swollen}}/D_{dry}}{L_{\mathrm{swollen}}/L_{dry}}$$

The derivation of this equation is reported in the Supporting Information. The amount of anisotropy tracks with the amount of swelling, in that more highly swollen samples are less able to overcome the resistance in the thickness direction that is imposed by the experimental setup, as shown in Figure S1.

With both network modulus and equilibrium swelling in hand, it is possible to calculate cross-link density of the gels. Table 5 presents the molecular weight between cross-links (*M*_c_) and the corresponding cross-link densities (ρ/*M*_c_) for the set of PEGDA hydrogels. Molecular weight between cross-links was calculated using the following equation,^23^ which assumes that water is a theta solvent for PEG (a reasonable assumption^55^):

8 $$M_{c}=\frac{kTM_{0}}{b^{3}}\phi_{o}^{2/3}\phi_{p}^{1/3}\frac{1}{G^{\prime }\left(\phi \right)}$$

The values of *M*_c_ were determined for the dry and swollen states of the hydrogels. The as-formed gel volume fraction, ϕ_o_, is assumed to be the same as the polymer volume fraction in the precursor solution. The polymer volume fraction at the time of modulus measurement is ϕ_p_, which is 1 in the dry state and taken as ϕ_p_ (*w*_p_) for the fully swollen state (see Table 5). Other definitions and values of variables appearing in eq 8 are reported in Table 6. Our results show that the PEGDA700 gel has the lowest *M*_c_ and highest cross-link density compared to PEGDA4k, 8k, and 10k gels. The data suggests that as the molecular weight of PEGDA increases the *M*_c_ also increases, which corresponds to a decrease in cross-link density. The precursor polymer is end functionalized such that the network would ideally have an *M*_c_ that is equivalent to the precursor polymer molecular weight. This is approximately true for the two lowest molecular weights, noting that there is considerable uncertainty in the result, which is a known challenge in the study of gels due to a variety of factors that include sensitivity to slight variations in gel yield and variability in distribution of cross-links.^55,56^ Our results compare reasonably well with other literature reports that used either swelling or modulus exclusively to determine *M*_c_.^55^ This agreement can be seen in the comparison of our swelling results to those of ref 54 in Figure S23. We note that an advantage of locating cross-linking functionality at chain ends is elimination of the need for corrections for dangling chain ends. This structural difference can significantly affect the calculation of *M*_c_ and the interpretation of swelling behavior, making direct quantitative comparisons invalid.^57^ Qualitatively, all studies report poorer prediction of *M*_c_ with increasing precursor polymer molecular weight, due to a combination of factors that include entanglements, specific interactions, and imperfections resulting from dilution of cross-link functionality in the precursor solution.

**Table 5:** Swollen Polymer Volume Fraction, *ϕ*_p_, Molecular Weight between Cross-Links, *M*_c_, and Cross-Link Density, ρ/*M*_c_ ^a^

sample name	ϕ_p_(*w*_p_)	*M*_c_(*G*_dry_^′^) (g/mol)	*M*_c_(*G*_swollen_^′^) (g/mol)	ρ/*M*_c_(*G*_dry_^′^) (mol/m^3^)	ρ/*M*_c_(*G*_swollen_^′^) (mol/m^3^)
PEGDA700 gel	0.34	2,759	640	395	1,704
PEGDA4k gel	0.12	4,292	2,102	254	519
PEGDA8k gel	0.06	15,247	28,478	71	38
PEGDA10k gel	0.11	16,526	678	66	1,607

a ρ/*M*_c_ is calculated using PEG density at 70 °C (the temperature of the rheology measurement), where the sample is fully amorphous. ρ = 1.09 g/mL.^53^

**Table 6:** Physical Constants Used for Cross-Link Density Calculations

symbol	(units)	value	definition	source
*M*_o_	(g/mol)	44	Monomer molecular weight	
*b*	(nm)	0.75	Monomer segment length	^58^
*k*	(J/K)	1.38 × 10^–23^	Boltzmann’s constant	
*T*	(K)	340.65	Temperature	
ρ_PEG_	(g/mL)	1.09	Dry polymer density at 70 °C	^53^
ρ_w_	(g/mL)	0.97915	Water density at 70 °C	

Transient swelling behavior of the PEGDA hydrogels was investigated by monitoring the change in thickness over time with the hydrogel submerged in a solvent (DI water). Figure 5 illustrates the swelling kinetics for these hydrogels, where the thickness (normalized to the initial thickness) is plotted as a function of time. The swelling curves demonstrate a typical swelling profile, with a rapid initial increase in thickness that gradually approaches a plateau, indicating equilibrium swelling. The results show that the swelling *rate* varies with molecular weight, with lower molecular weight PEGDA gels swelling more quickly than higher molecular weight PEGDA gels. Specifically, the PEGDA700 gel swells rapidly, reaching approximately 90% swelling within 1000 s. PEGDA4k gel reaches a thickness of 90% of the maximum swelling by 2000 s. PEGDA10k gel shows a slower swelling rate, reaching a swelling of 90% in just under 10,000 s. PEGDA8k is an outlier due, we speculate, to the extremely large dispersity of molecular weight, as reported by the supplier.

**Figure 5:**
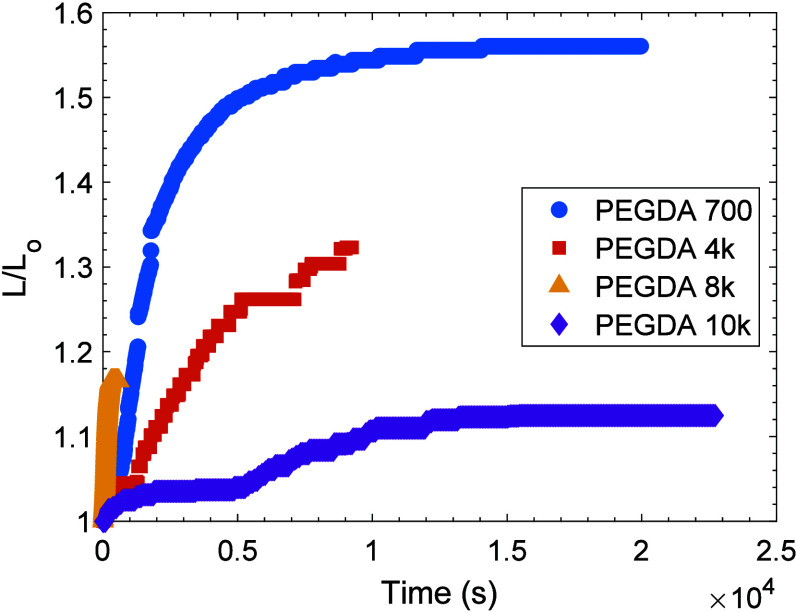
Nondimensionalized thickness of representative samples of PEGDA gels in DI water over time. Different markers represent each molecular weight: PEGDA700 gel (blue circles), PEGDA4k gel (orange squares), PEGDA8k gel (yellow triangles), and PEGDA10k gel (purple diamonds). The initial and final dimensional thicknesses in mm, *L*_0_ and *L*_f_, are, respectively, as follows: 3.590 and 5.431 for PEGDA700, 1.017 and 1.217 for PEGDA4k, 1.178 and 1.383 for PEGDA8k (which rapidly achieved the swollen value), and 1.756 and 1.975 for PEGDA10k.

The more rapid swelling of PEGDA700 gel is due, in large part, to the lack of crystallinity in the sample (Figure 4b). PEG crystals are known to immobilize water molecules during the crystallite dissolution process.^42^ Thus, crystallinity not only slows diffusion but also mechanically resists macroscopic swelling until the slow crystallite dissolution process is complete. The signature of crystallinity in Figure 4b appears for PEGDA4k gel and is significantly greater for PEGDA10k gel, which explains the gradual decrease in rate with increasing molecular weight (i.e., crystallinity). The decrease in *magnitude* of equilibrium swelling with increasing molecular weight is due to the stiffer amorphous PEGDA700 gel being able to generate sufficient force to raise the drop indicator and thereby swell isotropically. On the other hand, the highest molecular weight (PEGDA10k gel) exhibits highly anisotropic swelling, predominantly in the radial direction, due to the soft nature of the swollen, amorphous sample being unable to resist the gravitational weight of the drop indicator as well as the weight of the sample itself.

In order to quantitatively compare early rates of swelling across all PEGDA samples with different initial thicknesses and molecular weights, the thickness data was converted to equivalent solvent mass in a dimensionless form that ranges from zero at initial time to one at equilibrium. The conversion to equivalent mass enables a traditional sorption kinetics model to be used to empirically classify the type of sorption occurring,^59^ as follows:

9 $$\frac{M_{s}}{M_{s,f}}=\frac{L{\left(t\right)}^{3}-L_{0}^{3}}{L_{f}^{3}-L_{0}^{3}}=kt^{n}$$

Here, *M*_s_ is the mass of solvent absorbed at time *t*, *M*_s,f_ is the equilibrium solvent mass at infinite time, *L*(*t*) is the thickness at time *t*, *L*_0_ is the initial thickness, *L*_f_ is the final equilibrium thickness, *k* is the kinetic constant (with units of time^–*n*^), and *n* is the diffusional exponent that characterizes the nature of swelling.^59^ This approach, as described by Neogi, allows for a direct comparison of swelling kinetics regardless of the initial dimensions of the samples. By linearizing the early time mass-uptake data, we determined the *n* and *k* values which are listed in Table 7 and illustrated in Figures S25–S28. Early time data was defined as the region of time when thickness began to increase significantly and thus excludes any time lag associated with crystallite dissolution. This method provides a clear analysis of the swelling rate and behavior across different molecular weights and experimental conditions with the understanding that it will break down at long times in samples that swell anisotropically.

**Table 7:** Kinetic Sorption Model Fit Results: Coefficient, *k*, and Power, *n*, Values (Average and Standard Deviation Based on up to Three Repeat Experiments on at Least Three Different Samples) of All Gel Samples

symbol	*n*	*k* (s^*–n*^)
PEGDA700 gel	1.22 ± 0.72	(1.23 ± 2.13) × 10^–2^
PEGDA4k gel	1.06 ± 0.16	(2.63 ± 4.08) × 10^–4^
PEGDA8k gel	1.55 ± 0.71	(2.22 ± 2.21) × 10^–4^
PEGDA10k gel	2.32 ± 1.63	(3.42 ± 5.85) × 10^–5^

Figure 6 presents characteristic dimensionless swelling results of PEGDA gels and shows the section of data used to extract the *n* and *k* values. These data are also presented in linearized form in Figures S25–S28. The linearized slope indicates the mass uptake behavior, distinguishing between Fickian and non-Fickian diffusion, where *n* = 1/2 for Fickian diffusion, *n* = 1 for Case II diffusion, and *n* > 1 for Supercase II diffusion.^59^ The parameter *k*, represented by the linearized intercept, $\ln\left(\frac{M_{s}}{M_{s,f}}\right)=n\ln\left(t\right)+\ln\left(k\right)$ , is the empirical power law constant, with higher *k* values indicating a faster initial uptake. Table 7 shows the specific values of *n* and *k* from eq 9 fit to the data. All samples in this study exhibit Supercase II sorption kinetics.

**Figure 6:**
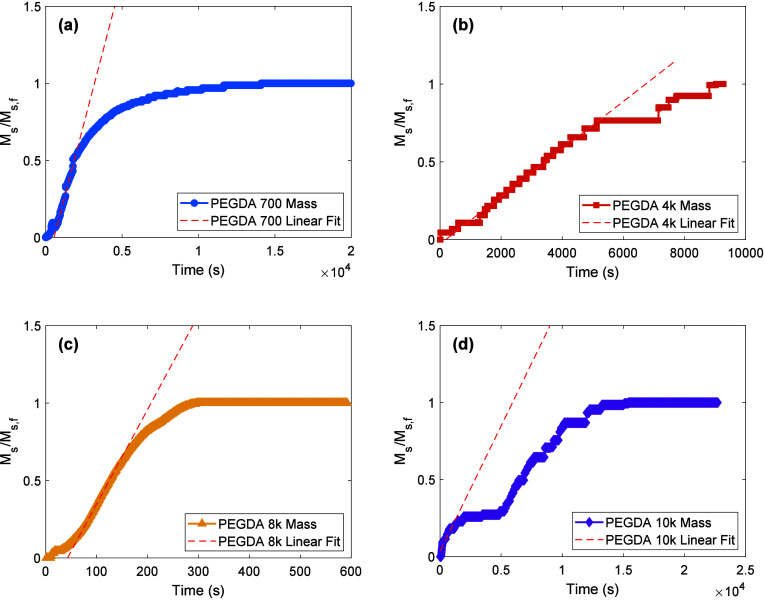
Dimensionless swelling versus time of the same 4 example PEGDA samples shown in Figure 5. The section of the thickness data overlapped by dashed lines represents the data used to extract *n* and *k* values from regression shown in Figures S25–S28. For PEGDA (a) 700, (b) 4k, (c) 8k, and (d) 10k, the *k* values are 9.25 × 10^–7^, 2.89 × 10^–5^, 1.68 × 10^–4^, and 6.01 × 10^–11^, while the *n* values are 1.74, 1.01, 1.63, and 4.13, respectively.

In order to examine how swelling kinetics evolve from early time to late time, characteristic times required to reach 5% swelling (τ_5%_) and 90% swelling (τ_90%_) were determined and are plotted against molecular weight in Figure 7, panels (a) and (b), respectively, as well as in Figures S29 and S30. This representation is sensitive to the absolute thickness of the sample and the qualitative swelling response (e.g., one- versus two-stage sorption), resulting in significant scatter. However, on average we observed that higher molecular weight PEGDA hydrogels exhibit slightly longer swelling times both initially (τ_5%_) and when nearing equilibrium (τ_90%_). This suggests that the correlation between molecular weight and swelling kinetics observed at an early time persists throughout the swelling experiment. These findings are consistent with previous studies that indicate larger polymer networks take longer to hydrate fully.^55^

**Figure 7:**
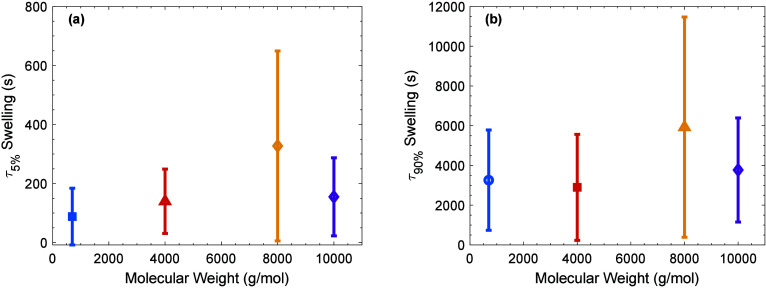
Swelling behavior of PEGDA hydrogels averaged with standard deviations as a function of molecular weight. (a) Time to 5% swelling (τ_5%_). (b) Time to 90% swelling (τ_90%_).

### Comparison of Model and Experimental Swelling Data

In this section, we show that the physical mechanism causing the sorption kinetics to follow Supercase II behavior is due to the moving boundary of the sample as a result of volumetric swelling. Previously, we developed a numerical model that accounts for swelling-based volumetric changes during solute diffusion.^22^ In order to apply the numerical model, an approximate diffusion coefficient is needed and was determined by applying Crank’s early time approximation for one-dimensional diffusion in a plane sheet.^60,61^

10 $$\frac{M_{s}}{M_{s,f}}\approx \frac{4}{\sqrt{\pi }}\sqrt{\frac{Dt}{L^{2}}}$$

*D* is the apparent diffusion coefficient, and *L* is the thickness of the sample. The apparent diffusion coefficient values for Figure 6 data are reported in Table S3, Table S4, and Figure S31.

Equations 2a and 2b were solved numerically by discretizing the sample thickness into small segments. The numerical model is a one-dimensional diffusion model that assumes (1) a constant diffusion coefficient, (2) isotropic swelling, and (3) volume additivity such that the increase in length of each differential element is the cubed root of the local water volume fraction. Thus, the diffusion path length increases as water diffuses into the network. The boundary conditions are no flux at the inner boundary and constant concentration at the outer boundary. The outer boundary concentration (in terms of solvent volume fraction) is calculated from Flory–Huggins Theory. The model predicts concentration profiles over time, as shown in Figure S32. These profiles were generated using the apparent diffusion coefficient determined above and illustrate the diffusion of water within the hydrogel. This prior work^22^ (eq 2a) includes an additional electro-osmotic flux term that accounts for the transport of water in the solvation shell of an ion migrating under an applied voltage gradient. The voltage gradient is set to zero in pure diffusion experiments and, by integrating across the samples, takes on a value of applied voltage/gel thickness in electro-osmotic measurements. As reported in our previous paper, volume additivity can be used to convert these profiles into sample thickness. Figure 8 presents the dimensionless linear sample thickness $\left(\frac{L\left(t\right)-L_{o}}{L_{f}-L_{o}}\right)$ from the numerical model and the experimental swelling data for PEGDA700 gel, which exhibited isotropic swelling. The numerical solution provides an excellent fit to the experimental data at early time, indicating the model’s ability to accurately capture the early time swelling kinetics. This indicates that the moving boundaries caused by large degrees of volumetric swelling can cause sorption kinetics to appear as Supercase II. A small deviation in the experimental data at later times characterized by the slowing of the rate and subsequent increase of the diffusive rate is noticeable and apparent in several other sample measurements especially for higher MW samples. We attribute this experimental behavior to nonidealities rooted in a second mechanism beginning to dominate the kinetics such as disentanglement.^55^ Comparisons between model predictions and experimental data for the other molecular weights are reported in Figures S33–S35. They demonstrate the deterioration of model prediction with increasing molecular weight due to increasing swelling anisotropy. Additional details of diffusion coefficient estimation are reported in the Supporting Information, Figures S36–S49.

**Figure 8:**
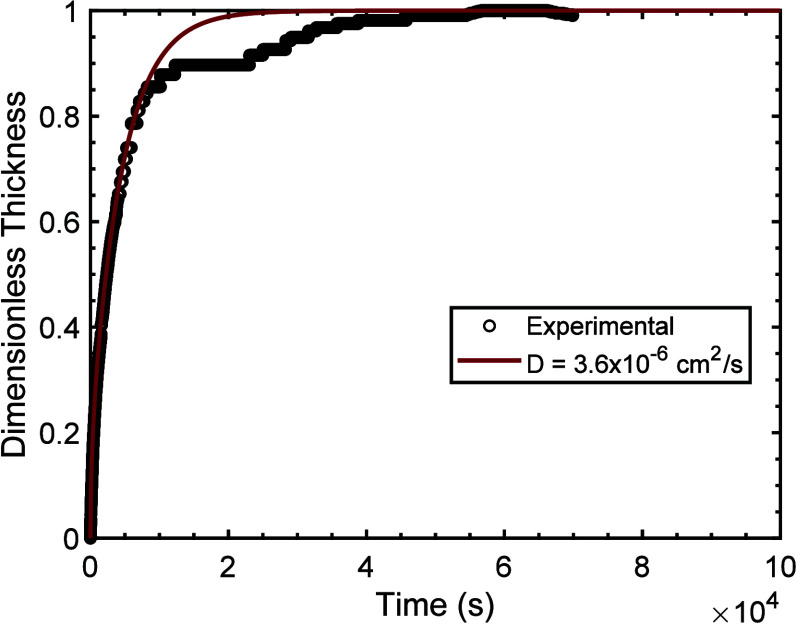
Transient dimensionless thickness of PEGDA700 gel and corresponding numerical solution from reference 22. The experimental swelling data are represented by black circles, while the numerical solution using the extracted early time diffusion coefficient, *D*, is shown by a dark red curve.

### Electroosmotic Drag

Figure 9 presents the swelling behavior of PEGDA4k hydrogel under applied voltages of ±0.35 and 0 V, with both model data (Figure 9a) and experimental data (Figure 9b) displayed. The normalized swelling ratio, $\frac{M_{s}}{M_{s,f}}$ , is plotted as a function of time. In the model data (Figure 9a), the curves for all applied potentials show an initial rapid increase in swelling, with the rates under +0.35 and 0 V being quite similar, and only a slight delay observed for −0.35 V. All three model curves eventually plateau as they approach equilibrium, with a nearly uniform progression toward this final state regardless of the applied potential.

**Figure 9:**
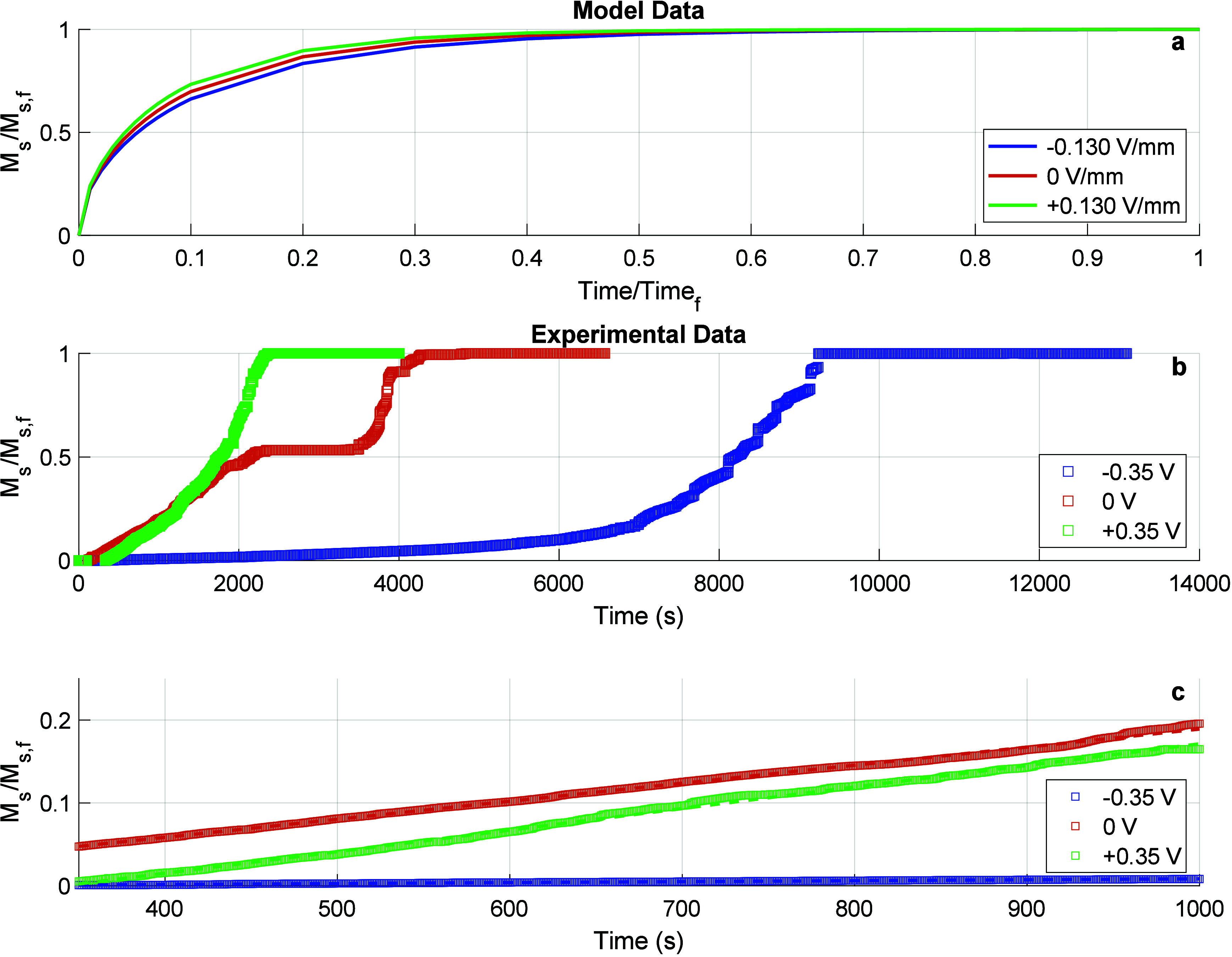
(a) Normalized model prediction of swelling influenced by migration caused by applied EMFs of −0.130 V/mm (−0.013 V), 0 V/mm (0 V), and +0.130 V/mm (+0.013 V). (b) Normalized swelling ratio, $\frac{M_{s}}{M_{s,f}}$ , as a function of time for 2.69 mm thick PEGDA4k hydrogel under applied voltages of −0.35 V (−0.13 V/mm), 0 V, and +0.35 V (+0.13 V/mm) in a 0.5 M NaCl solution. The main plot shows the progression of swelling over time, where significant differences between the voltage conditions can be observed, especially at later stages. (c) The third subplot zooms in on the initial swelling stages between 350 and 1000 s, highlighting the kinetic rate differences. The slopes in the early time window reveal distinct initial swelling rates for each applied potential. The neutral voltage (0 V) exhibits a two-stage swelling behavior, unlike the more uniform swelling observed under the EMF conditions.

In contrast, the experimental data (Figure 9b) reveal a more pronounced difference in swelling kinetics under the applied potentials. The +0.35 V experiment shows the most rapid swelling in the initial stages, followed by the neutral condition (0 V), while the −0.35 V case exhibits the slowest swelling rate. After approximately 2000 s, swelling under the +0.35 V condition quickly reaches equilibrium, while the neutral condition demonstrates a two-stage swelling process, with a noticeable transition before reaching equilibrium. Swelling under −0.35 V remains much slower throughout the experiment, only approaching equilibrium after 12,000 s. The time to 90% swelling for the data and the model is presented in Figure S50.

Figure 9c zooms in on the early stages of swelling, between 350 and 1000 s, providing a more detailed view of the swelling kinetics during this period. The difference in slopes between the curves under the various applied potentials highlights the differences in the initial swelling rates, with the +0.35 V condition clearly showing the steepest slope, corresponding to the highest swelling rate. The −0.35 V condition, on the other hand, exhibits the flattest slope, indicating minimal swelling during the early stages of the experiment. The model’s prediction of this early stage (Figure 9a) is not as differentiated as the experimental data, where the model predicts relatively similar early swelling behavior across all three voltage conditions. These slopes were quantified with the same power law approach used previously, and those values are reported in Table S5.

The differences between the experimental and model results highlight the complex role of electro-osmotic drag and ionic migration in influencing the swelling behavior under applied EMFs. The model’s uniform prediction contrasts with the experimental results, particularly in the initial stages of swelling, where the experiment shows significant variance between the positive and negative potentials. This suggests that further refinement of the model may be necessary to fully capture the ion-specific transport dynamics that drives these asymmetric swelling behaviors. In particular, the magnitude of electro-osmotic drag is sensitive to the solvation number of the migrating ion(s). In this work, it was set to a value of one but could be used as a fitting parameter to improve agreement between model and data.

## Conclusions

This study provides a comprehensive characterization of PEGDA hydrogels, examining both their chemical and mechanical properties as well as their behavior under applied EMFs. FTIR and NMR were used to confirm acrylate functionalization of PEG and subsequent cross-linking into gels, while rheological tests provided insights into the storage moduli and cross-link densities of the PEGDA gels. The acrylate cross-links were found to contribute to moduli at low molecular weights (700 and 4k), but 8k and 10k hydrogel moduli are predominantly dictated by the PEG network, resulting in sensitivity to chain dispersity observed as anomalous swelling behavior of the 8k hydrogel. The study found that cross-link density decreases with increasing precursor PEG molecular weight, resulting in looser networks that take up more water when swollen. Swelling experiments demonstrated the significant impact of cross-link density on water uptake rates, with PEGDA700 gel exhibiting rapid swelling and higher modulus compared to the larger molecular weight PEGDA gels. The combination of experimental data with theoretical modeling advanced the understanding of both solvent diffusion and mechanical performance by providing quantitative insights into ion migration dynamics, cross-link density, and their effects on swelling kinetics, allowing for more accurate predictions of hydrogel behavior under varying environmental and applied conditions. While the model should be modified to account for crystallite dissolution as well as the hydration shells of cations, it serves as a starting point for an enhanced predictor of behavior where more detailed parameters can be added to better capture hydrogel behavior.

The investigation of the effect of applied EMFs on the swelling behavior of PEGDA hydrogels revealed asymmetric control over the ion migration through the gel. Under positive EMF (+0.35 V), sodium ions migrate from the positively charged bottom electrode toward the top electrode, enhancing the solvent uptake and increasing the swelling rate. In contrast, under negative EMF (−0.35 V), sodium ion migration opposes the direction of diffusion-based swelling, leading to a significant reduction in swelling rate compared to neutral conditions. As shown in Figure 10, the negative-voltage slowing observed in experiments is significantly greater than migration-based acceleration due to positive voltage. The model, however, underpredicts the magnitude of the migration effect, highlighting the model’s assumption that one water molecule is dragged per sodium ion and indicating that a sodium hydration shell of order 10 is more appropriate. This indicates that the swollen state of the hydrogels does not significantly perturb the solvation shell of Na^+^ with respect to that in bulk water.^62^

**Figure 10:**
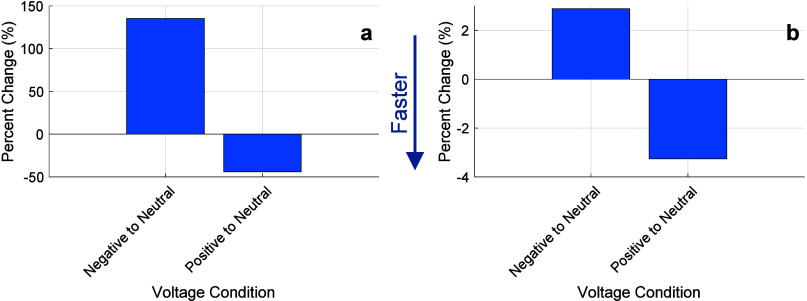
Percent change in the time to 90% swelling for PEGDA4k gel under different voltage conditions. (a) Experimental results showing a 135.3% increase in swelling time under negative volts compared to neutral and a 44% decrease under positive volts relative to neutral. (b) Model predictions show a much smaller percent change, with a 2.9% increase in swelling time under negative volts and a 3.3% decrease under positive volts compared to neutral.

In conclusion, this study highlights the responsiveness of PEGDA hydrogels to applied EMFs, demonstrating their potential for controlled, asymmetric swelling behavior driven by ion migration. The ability to manipulate swelling kinetics through the application of EMFs opens new avenues for optimizing these hydrogels for precision control in various applications. The results lay a foundation for further advancements in tuning ion migration and electro-osmotic behavior to achieve desired swelling rates, offering potential improvements in soft robotics and biomedical devices. Future work can build upon these findings by refining the model to incorporate additional ion-specific transport dynamics and exploring alternative ion concentrations, geometries, and materials. With these enhancements, such as addition of charge to the backbone, PEGDA hydrogels could become an even more versatile and effective tool in fields requiring responsive, load-bearing materials with tailored swelling properties.

## Supplementary Material



## Notes

Experimental setup, chemical characterizations, rheology, swelling and diffusion, and additional references (PDF)
