# Supplementary material for: Diffusive and Electro-osmotic Swelling of Neutral and Ion-Containing Poly(ethylene glycol) Hydrogels
Source: ACS Omega. 2025 Aug 19;10(34):38413–26. doi: 10.1021/acsomega.5c00865 (PMC12409565; doi:10.1021/acsomega.5c00865)
Supplement: Supplementary file 1 [file ao5c00865_si_001.pdf]

Supporting Information for

Diffusive and Electro-osmotic Swelling of Neutral and Ion-Containing Poly(ethylene glycol) Hydrogels

*AUTHOR NAMES. Shefik D. Bowen, Lexy D. Herrera, Daniel T. Hallinan**

AUTHOR ADDRESS. Florida A&M University-Florida State University (FAMU-FSU) College of Engineering, Department of Chemical and Biomedical Engineering and Aero-propulsion, Mechatronics, and Energy Center, Tallahassee, FL, 32310, USA

Contents

Experimental Setup 2
Chemical Characterization. 3
Rheology 15
Swelling and Diffusion. 27
References 57

Experimental Setup

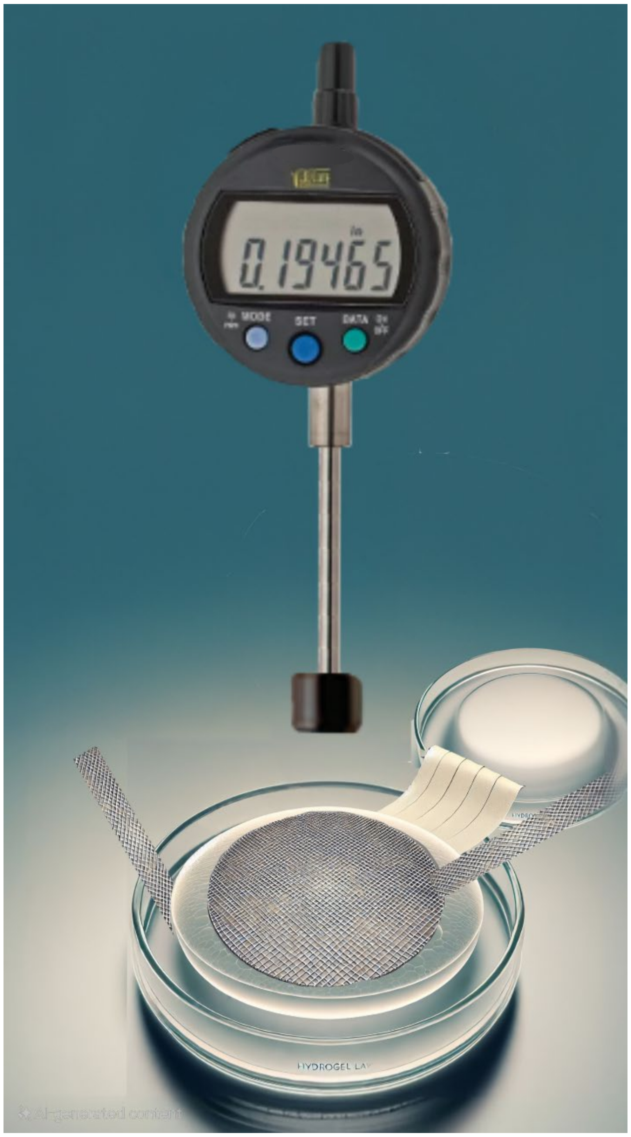

Figure S1. Experimental setup for measuring gel samples with nickel mesh ensuring solvent flow to polymer surface. Filter paper is shown wicking solvent from a reservoir to the Petri dish.

Chemical Characterization.

Figure S2 illustrates the ^{1}H NMR spectra of the acrylate-functionalized polyethylene glycol diacrylate (PEGDA) samples. The main spectrum provides a broad overview of the chemical shifts, while the insets focus on specific regions: acrylate peaks and the OH absorbance region.

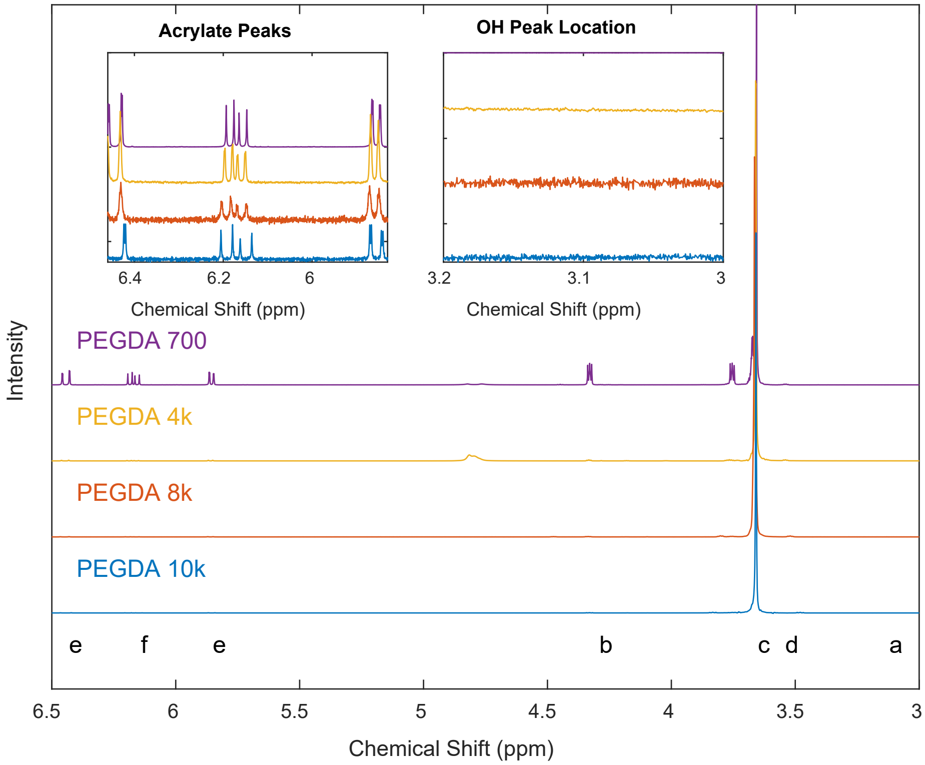

Figure S2. Normalized NMR spectra of PEGDA samples. The absence of peaks between 2.8 and 3.4 ppm confirms the acrylation reaction, which removes the OH signal at this chemical shift. The insets highlight the acrylate peaks and OH peak location.

In the Figure S3 (a) inset labeled Backbone Peaks, the spectra reveal that the chemical shifts of the backbone protons do not perfectly align between PEG 4k and PEGDA 4k. This misalignment can be attributed to the chemical modification process, where the introduction of acrylate groups alters the electronic environment around the backbone protons[1]. Despite this shift, we confidently assign these peaks to the backbone protons due to their consistent presence and relative position in both spectra. The acrylate peak inset highlights the new peaks present in the

PEGDA 4k spectrum, confirming the successful functionalization of PEG 4k with acrylate groups. The OH peak inset shows the proton signals from the hydroxyl groups, further supporting the structural changes upon acrylation. Similar behavior is exhibited by the 8k and 10k samples in panels (b) and (c) respectively.

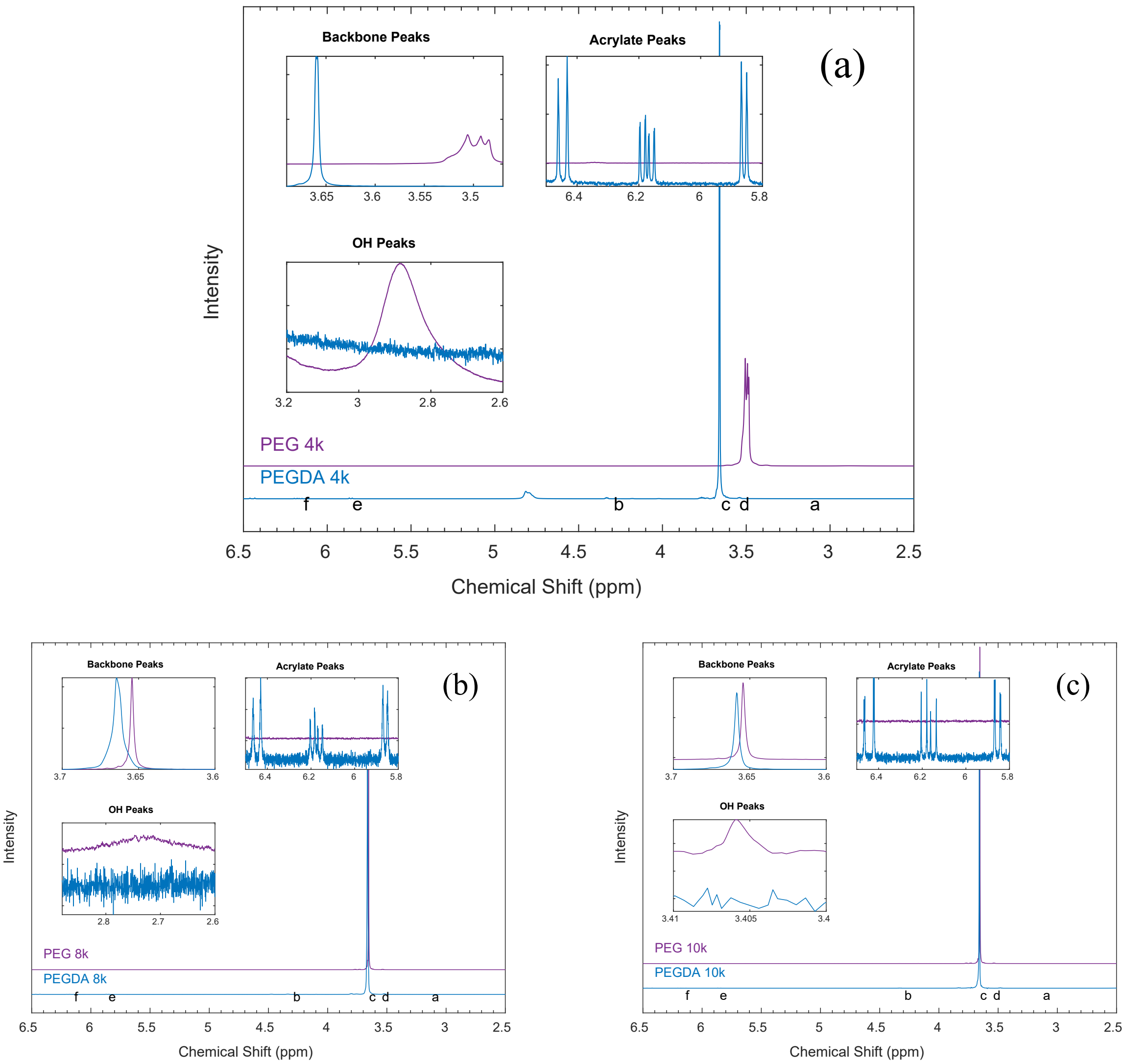

Figure S3. Comparison of 1H NMR spectra of (a) PEG 4k and PEGDA 4k, (b) PEG 8k and PEGDA 8k, and (c) PEG 10k and PEGDA 10k. The main spectra show the overall chemical shifts of PEG (purple) and PEGDA (blue). The insets highlight specific regions of interest: Backbone Peaks (3.65–3.5 ppm), Acrylate Peaks (6.4–6.0 ppm), and OH Peaks (3.2–2.6 ppm).

To corroborate our NMR findings, we utilized Fourier transform infrared – attenuated total reflectance (FTIR-ATR) spectroscopy (Figure S4), which provided additional evidence of the acrylate functional groups, around 1700 cm^{-1}. The combined analysis from both NMR and FTIR-ATR spectroscopy allows us to confidently assign the large peaks in the backbone region to the backbone protons, despite their slight chemical shift discrepancies. This comprehensive approach confirms the successful synthesis of PEGDA 4k from PEG 4k.

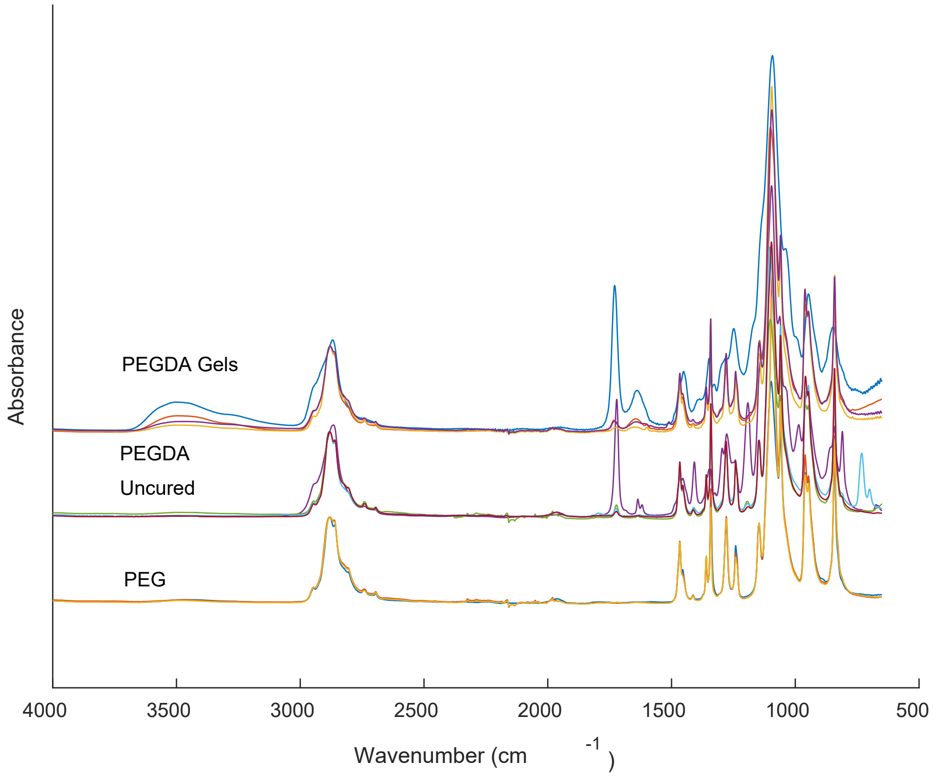

Figure S4. Normalized FTIR-ATR spectra of various molecular weights offset into groups (PEG, PEGDA uncured, PEGDA Gel).

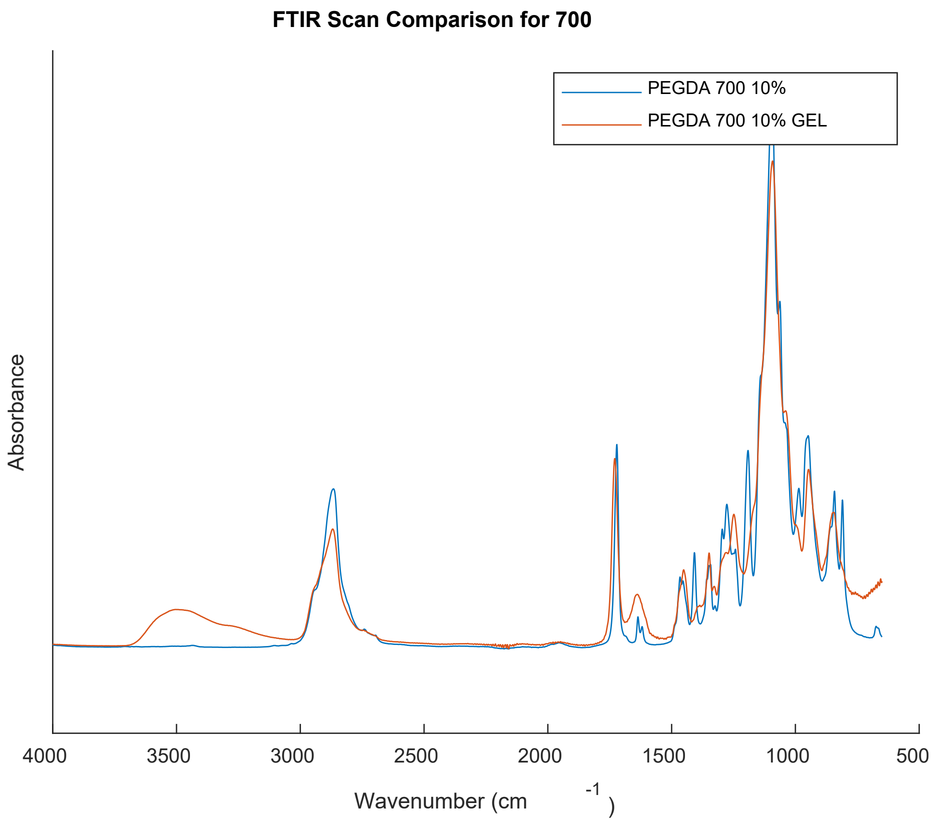

Figure S5. FTIR-ATR comparison of PEGDA 700 10% gel to PEGDA 700 Precursor liquid.

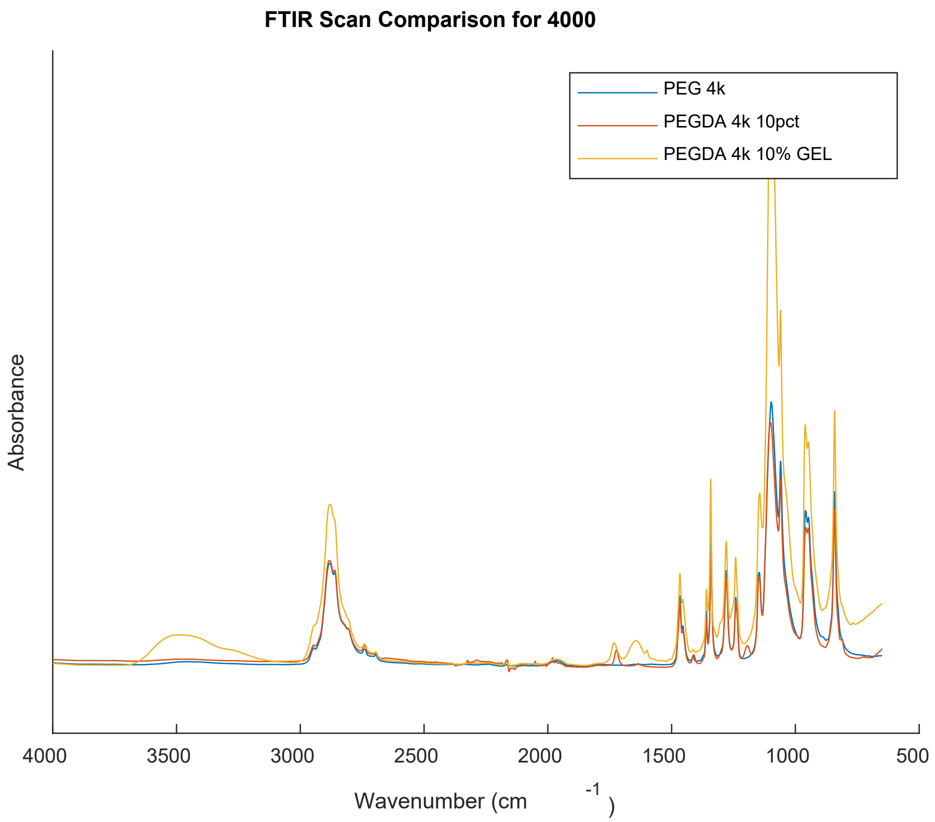

Figure S6. FTIR-ATR comparison of PEGDA 4k 10% Gel to PEGDA 4k.

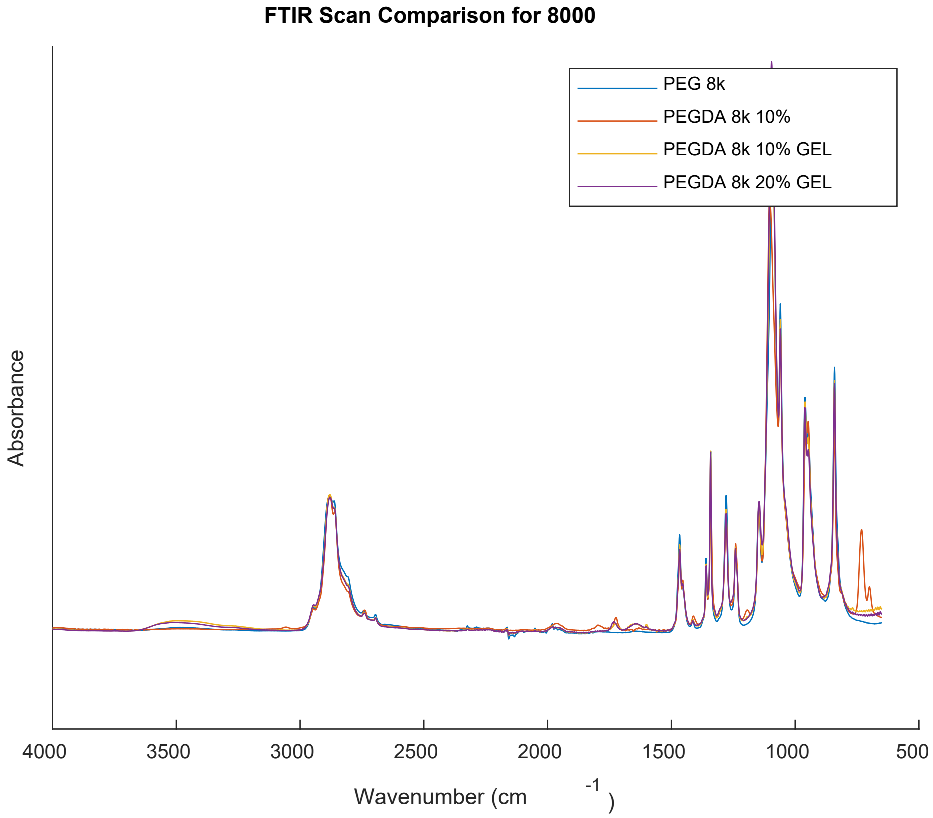

Figure S7. FTIR-ATR comparison of PEGDA 8k 10% Gel to PEGDA 8k.

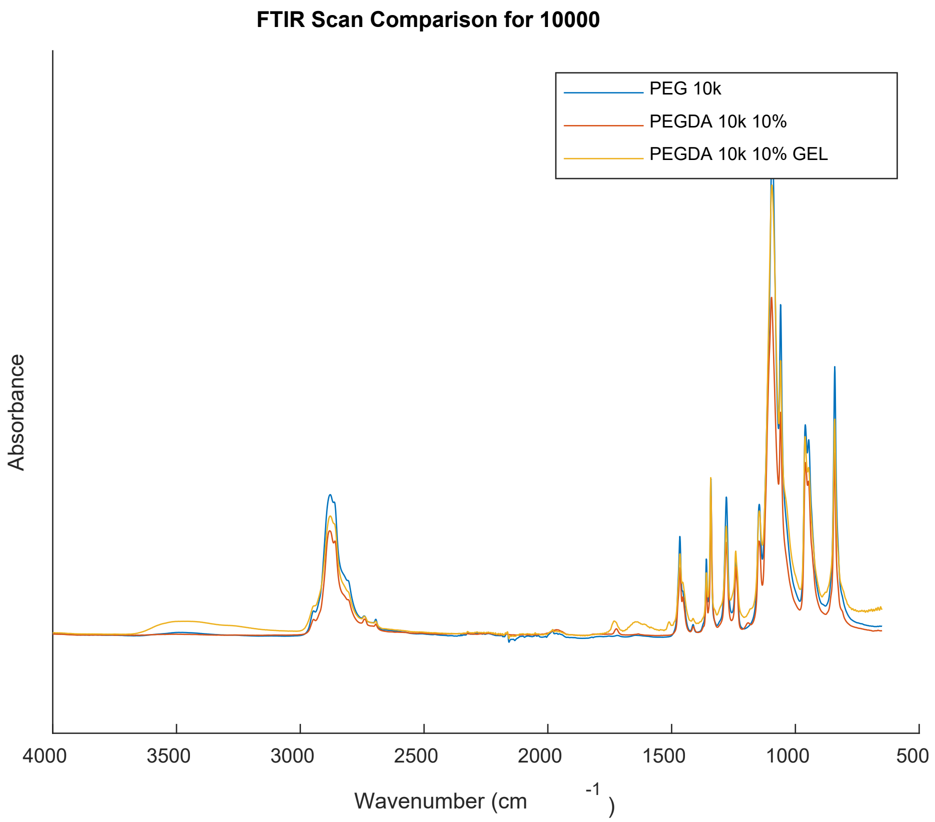

Figure S8. FTIR-ATR comparison of PEGDA 10k 10% Gel to PEGDA 10k.

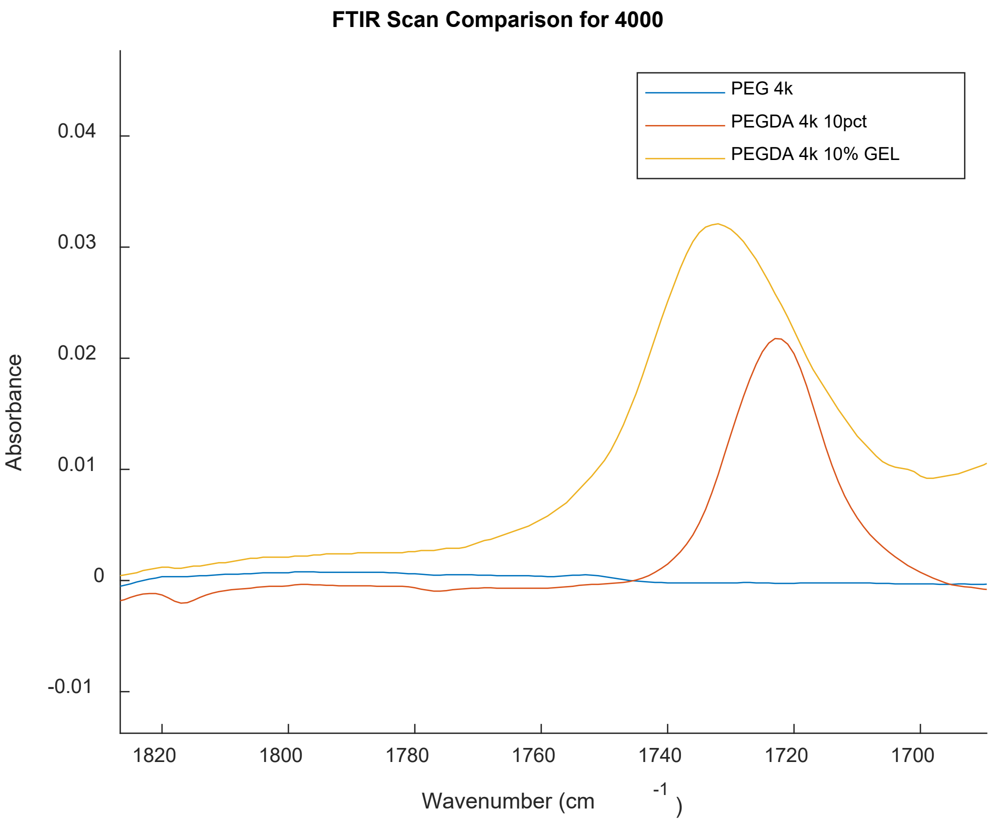

Figure S9. FTIR-ATR spectrum of PEG, PEGDA, and PEGDA Gel 4k zoomed to 1700 to 1820 cm^{-1}.

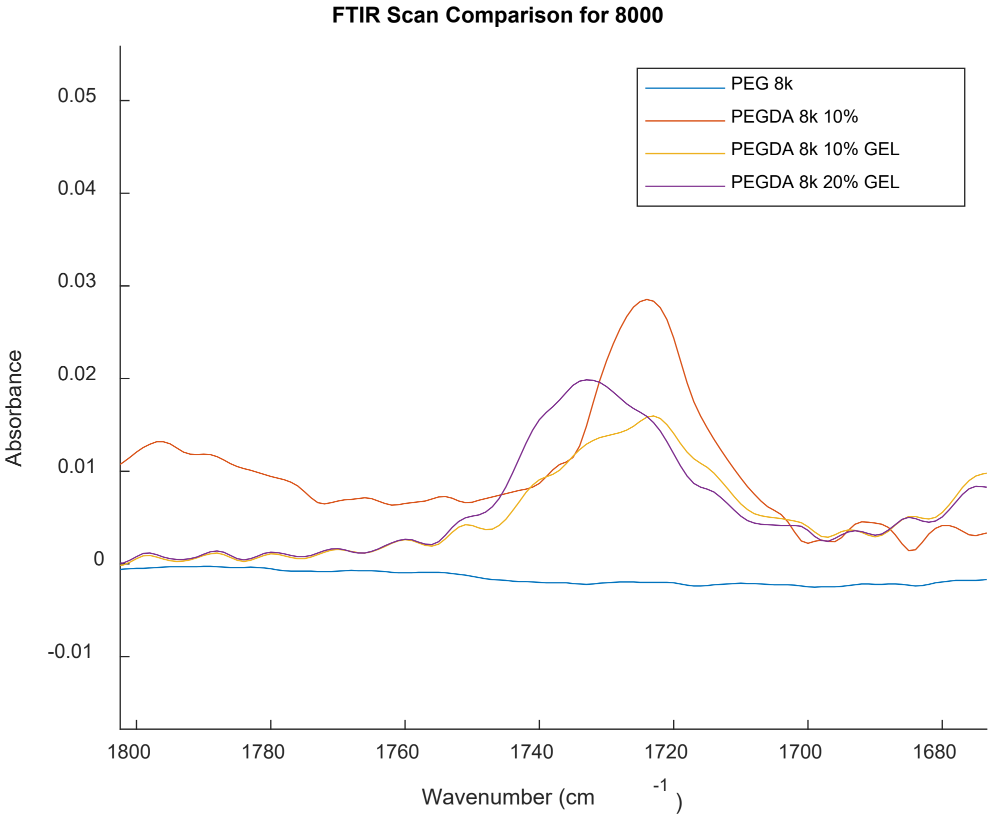

Figure S10. FTIR-ATR spectrum of PEG, PEGDA, and PEGDA Gel 8k zoomed to 1700 to 1820 cm^{-1}.

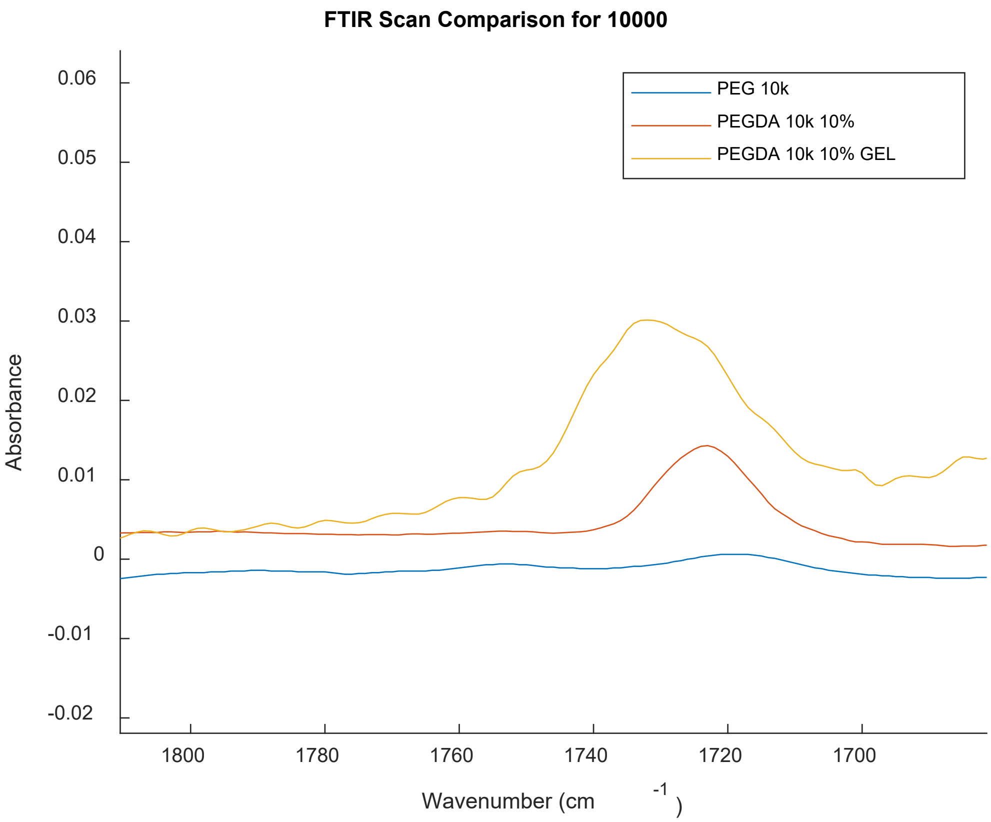

Figure S11. FTIR-ATR spectrum of PEG, PEGDA, and PEGDA Gel 10k zoomed to 1700 to 1820 cm^{-1}.

DETREND OF FTIR-ATR SPECTRA PROCEDURE:

The detrending methodology is designed to eliminate linear trends from the transmittance data of PEG, PEGDA powder, and PEGDA gel samples. These trends may arise due to instrumental or experimental conditions rather than the chemical characteristics of the samples. Below is a detailed description of the steps involved in this process:

First, the data is read from a CSV file where the wavenumber is denoted as x and the transmittance measurements as y. The absorbance is then calculated using the formula $\left(Absorbance = 2 - log_{10}(y)\right)$, which converts the transmittance data (in %) into absorbance by subtracting the base-10 logarithm of the transmittance from 2.

Next, a change detection process is initiated using the "*ischange*" function with the 'linear' option to detect points where significant linear trends occur in the transmittance data. This function calculates slopes, S_l, of the potential linear segments and identifies change points C_p.

A histogram of the slopes is then created using 50 bins (histcounts), which categorizes the slopes into different ranges. The histogram bin with the highest count (Max) is identified, corresponding to the most frequent slope value (Binmax). This value indicates a prevailing linear trend in the data.

To identify the linear region, a logical vector "LinearRegion" is created, marking areas where the slopes are equal to the most frequent slope. A linear fit (polyfit) is then performed on this identified linear region, resulting in coefficients (B) that describe the best-fit line.

The linear fit is evaluated polyval over the entire range of wavenumber data (x), producing a linear trend line (L). This linear trend L is subtracted from the original transmittance data (Transmittance), resulting in detrended transmittance data (y). This subtraction process effectively

removes the identified linear trend, allowing for the analysis of the intrinsic spectral features of the sample.

Finally, the detrended data is plotted against the wavenumber for visual analysis. The plot is configured with specific axes limits and directions, and a legend is included for clarity. This systematic approach ensures that any subsequent analysis, such as peak identification or quantitative analysis, is based on features inherent to the sample, free from the effects of non-sample-related trends in spectral data.

Rheology

Figure S12 displays the storage modulus G′ of PEGDA samples with different molecular weights as a function of time during UV curing. Each dataset is represented by markers of different colors: blue for PEGDA 700, orange for PEGDA 4k, red for PEGDA 8k, and purple for PEGDA 10k. Measurements range from 10^{-4} Pa to 10^{6} Pa, while the x-axis represents time in seconds.

Initially, at low modulus levels (below 1 Pa), significant noise is observed in the data. This noise is evident from the scattered and erratic points at the beginning of the time for all samples. This noise indicates the challenges faced by the rheometer in recording precise data within the low modulus regime. Such noise arises because the rheometer operates at its detection limits, especially for the uncured liquid PEGDA samples, which have very low initial moduli.

Once the PEGDA samples begin to cure and transition into a gel state, their moduli increase rapidly, becoming more stable and significantly reducing noise. The storage modulus levels off, indicating the formation of a solid gel. The linear viscoelastic regime, appropriate for a solid gel, is reached, and the measurements become more reliable and consistent.

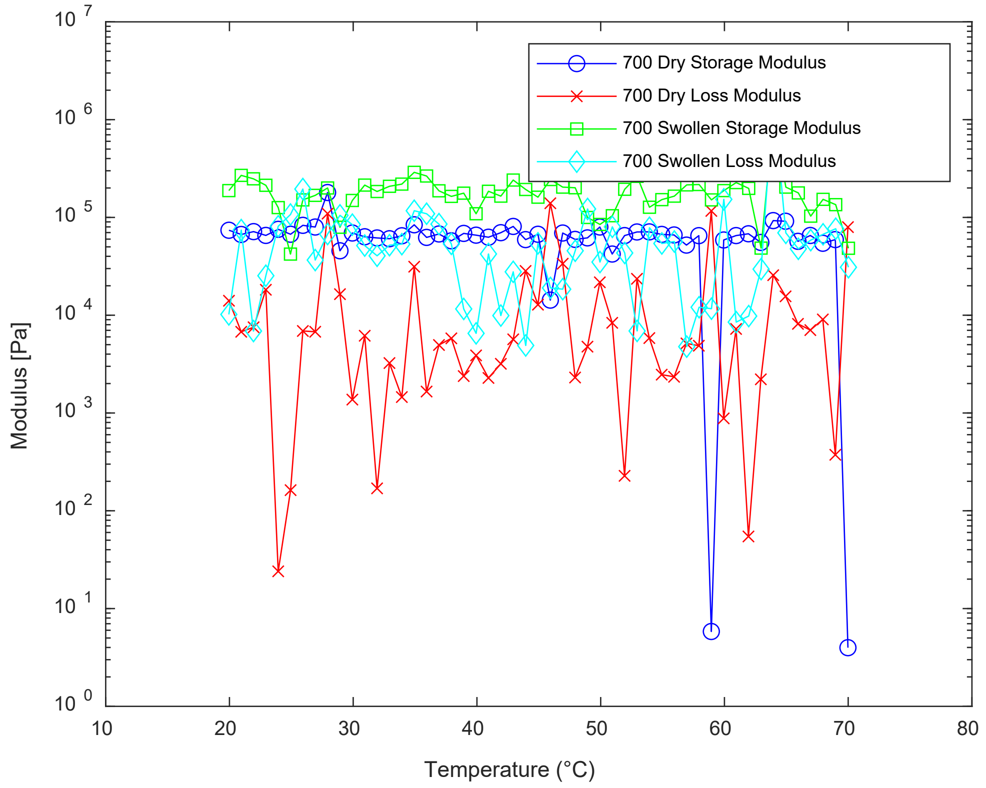

Figure S12. PEGDA 700 Gel dry storage and loss moduli and swollen storage and loss moduli.

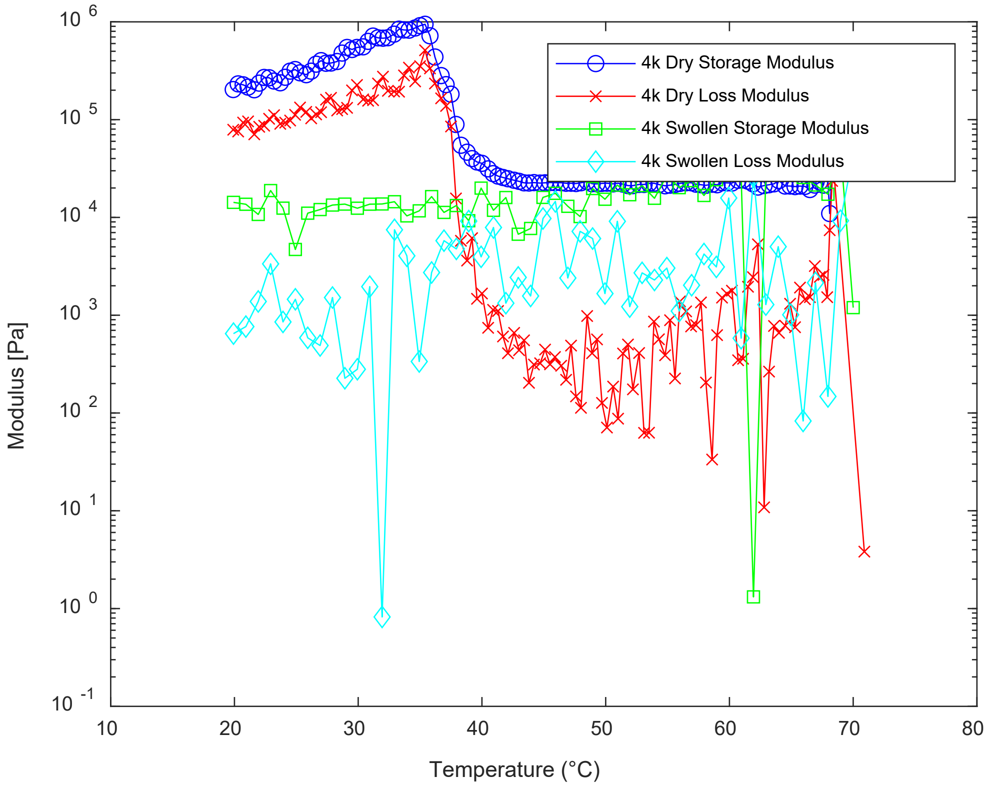

Figure S13. PEGDA 4k Gel Dry storage and loss moduli and swollen storage and loss moduli.

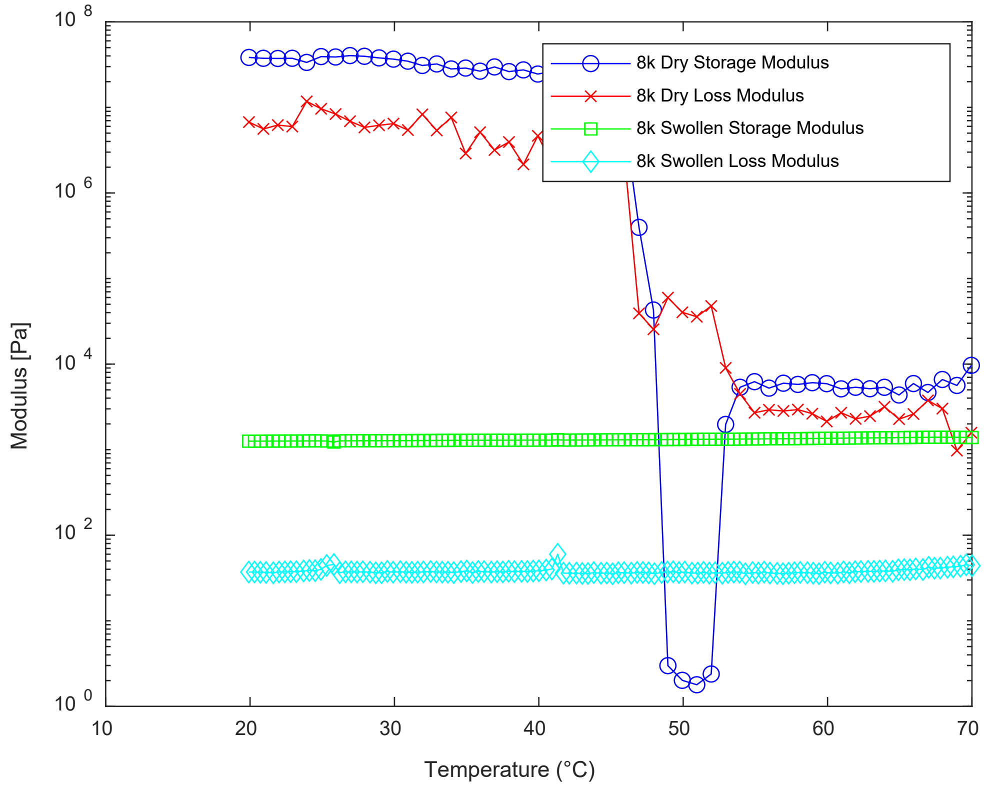

Figure S14. PEGDA 8k Gel dry storage and loss moduli and swollen storage and loss moduli.

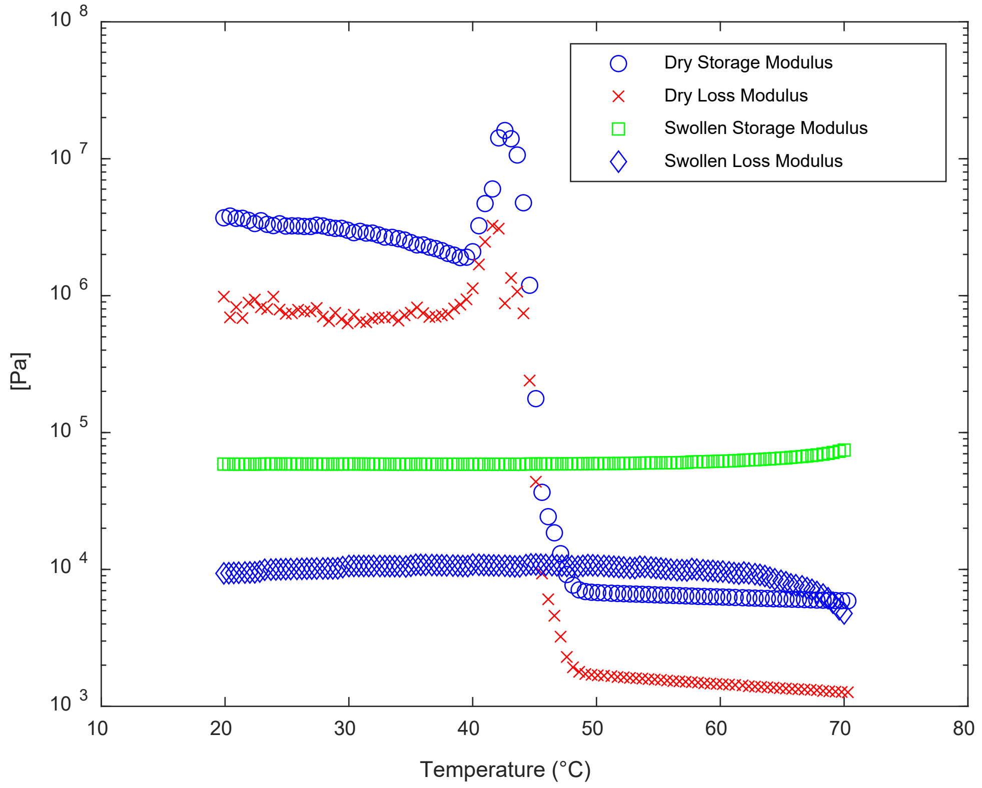

Figure S15. PEGDA 10k Gel dry storage and loss moduli and swollen storage and loss moduli.

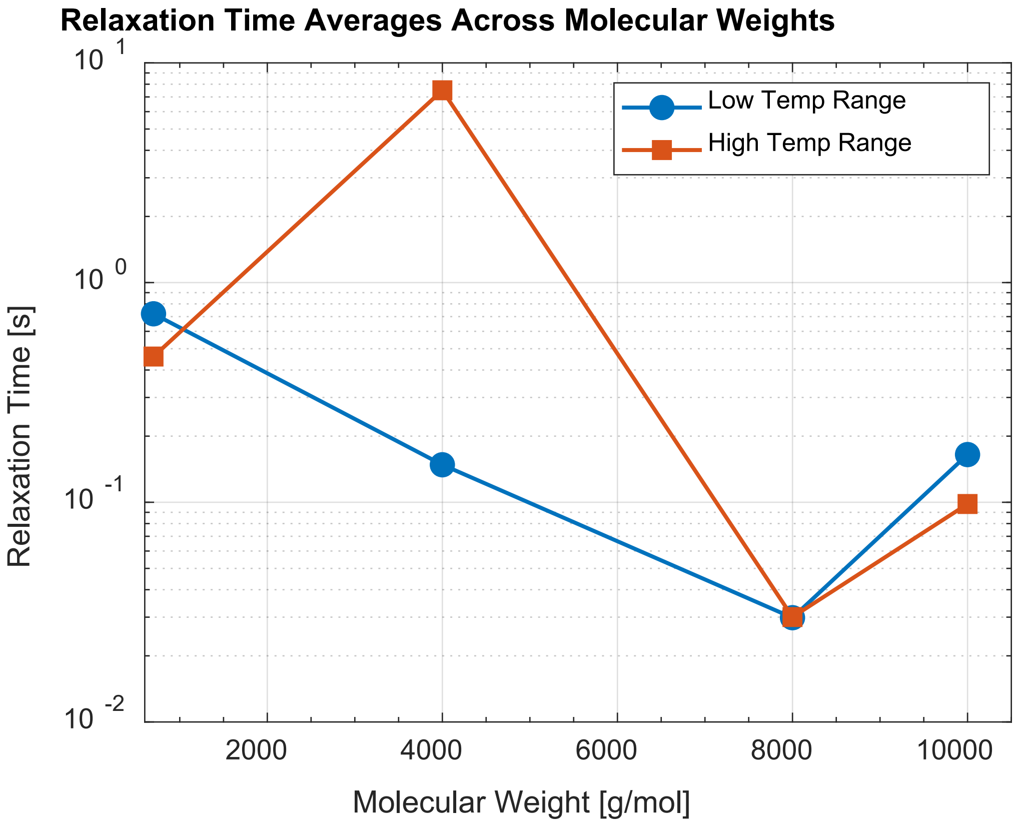

Figure S16. Average relaxation times for PEGDA samples with different molecular weights (700 g/mol, 4000 g/mol, 8000 g/mol, and 10,000 g/mol) at low (20-25°C) and high (65-70°C) temperature ranges. Relaxation times were calculated using $\tau_{relax} = \left(\frac{G''}{G'}\right)\left(\frac{1}{\omega}\right)$, where G″ is the loss modulus, G′ is the storage modulus, and ω is the angular frequency. The low temperature range, represented by blue circles, captures the glassy state behavior, while the high temperature range, represented by red squares, highlights the rubbery state behavior. The y-axis is plotted on a logarithmic scale to emphasize differences in relaxation times across temperature regimes.

Table S1. Relaxation times based on storage moduli (G') and loss moduli (G") for samples averaged between 30 – 45 °C (Low Temp) and 45 – 70 °C (High Temp).

Molecular Weight g mol	$\tau_{Low\ Temp}$ (s)	$\tau_{High\ Temp}$ (s)
PEGDA 700 Gel	4.28×10^{-01}	3.49×10^{-01}
PEGDA 4000Gel	3.49×10^{-01}	8.02×10^{02}
PEGDA 8000 Gel	2.94×10^{-02}	2.79×10^{-02}
PEGDA 10000 Gel	1.82×10^{-01}	1.52×10^{-01}

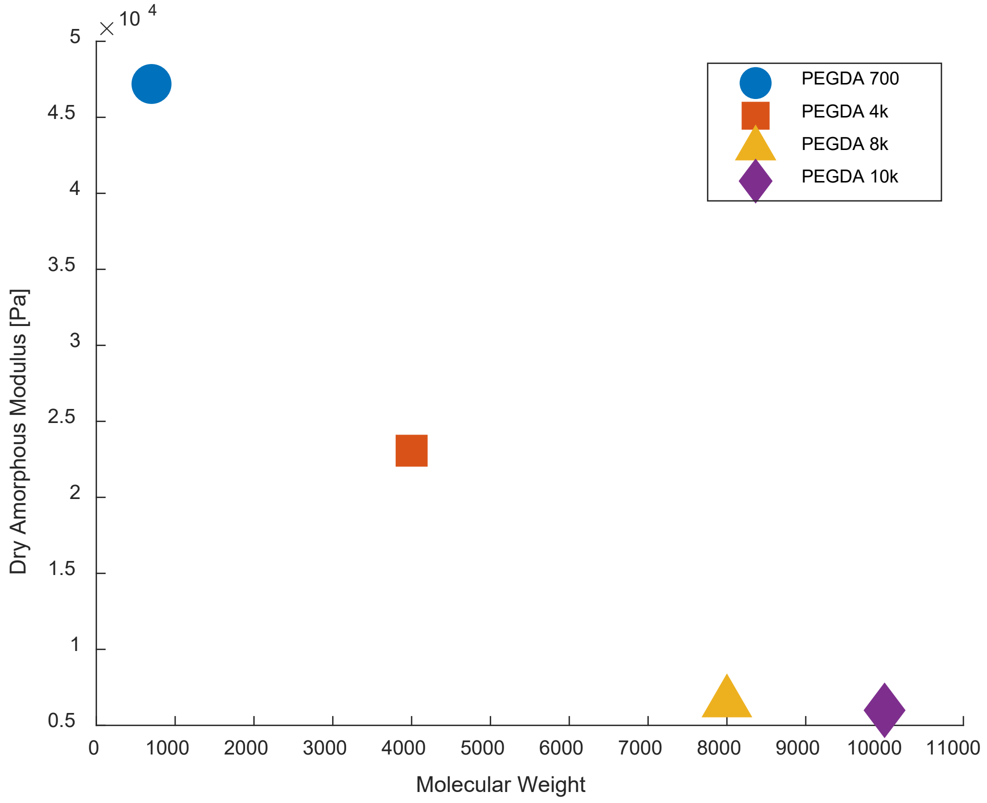

Figure S17. Dry amorphous storage modulus between 65 C and 70 C for various molecular weights.

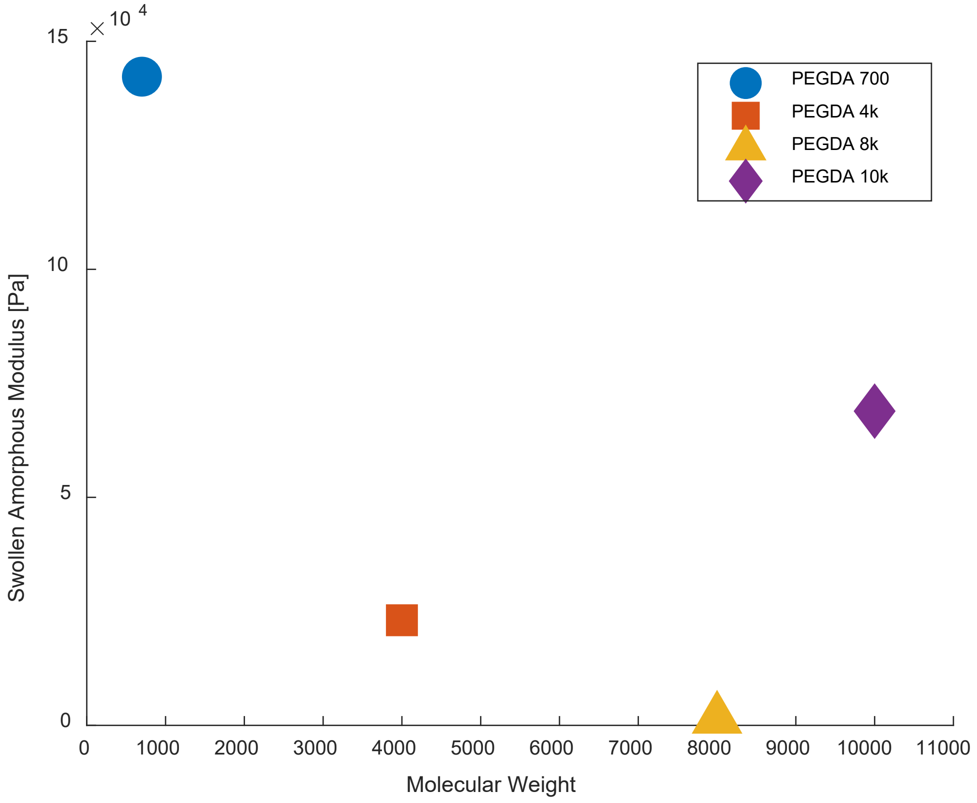

Figure S18. Swollen amorphous storage modulus between 65 C and 70 C for various molecular weights.

Figure S18 illustrates the swollen storage moduli of PEGDA gels with molecular weights of 700, 4k, 8k, and 10k g/mol at 10% gelation concentration and 100% swelling. The data show a pronounced decrease in storage modulus as the molecular weight increases from 700 to 8,000 g/mol, followed by a substantial increase at 10,000 g/mol. This trend suggests that lower molecular weight PEGDA forms a denser and stiffer network, while intermediate molecular weights result in a looser network structure, reducing the modulus. However, at higher molecular weights, such as 10,000 g/mol, the network possibly becomes more entangled, leading to an increase in modulus. According to Lodge and Bard & Faulkner, such behavior can be attributed to the balance between crosslinking density and chain entanglement. The dip in modulus values at intermediate molecular weights implies a less efficient crosslinking and network formation, which could affect the

mechanical stability and potential applications of the hydrogels in biomedical and soft robotics fields, where precise control over mechanical properties is crucial.

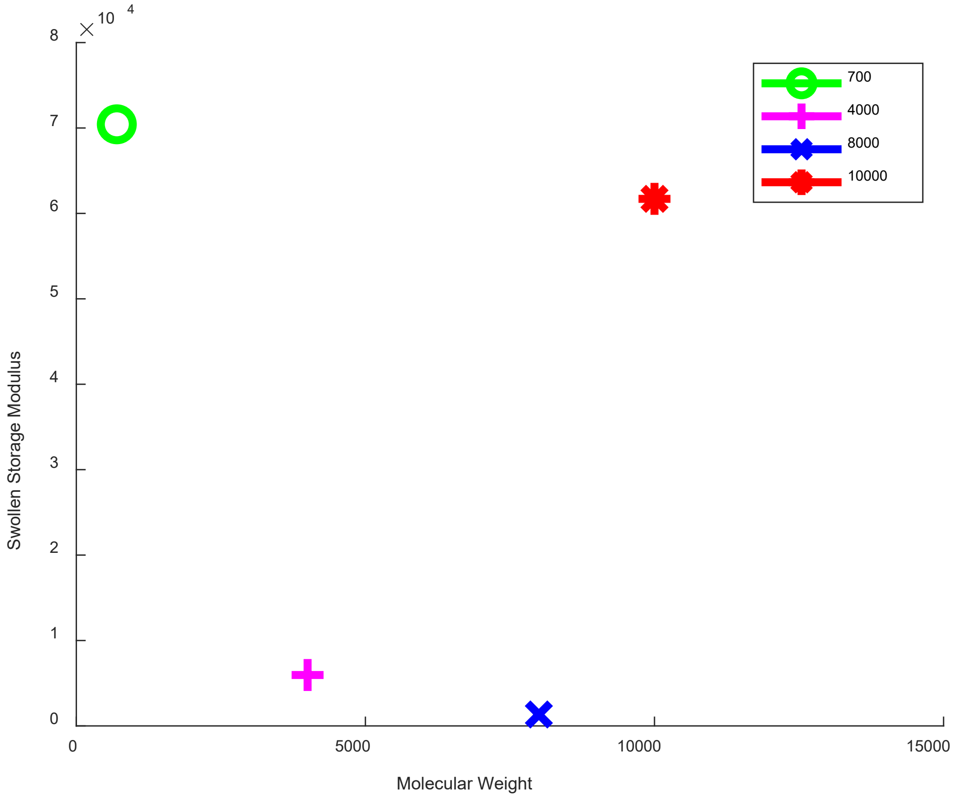

Figure S19. Storage moduli of PEGDA gels 700, 4k, 8k, 10k g/mol at 10% gelation concentration at 100% swelling. Measurements were taken using an Anton Paar MC-304e.

Table S2 presents the amorphous storage moduli ($G'_{amorphous}$) of polyethylene glycol diacrylate (PEGDA) hydrogels across a range of molecular weights from 700 to 10,000 g/mol. The storage modulus, a measure of the hydrogel's stiffness, is crucial for understanding the mechanical properties relevant to their potential use in applications such as artificial muscles and soft robotics. The data shows a trend where lower molecular weight hydrogels (700 g/mol) have significantly

higher modulus values, indicating stiffer materials, whereas intermediate molecular weights (4000 and 8000 g/mol) exhibit considerably lower moduli, suggesting softer materials. The modulus increases again at the highest molecular weight (10,000 g/mol). This trend could imply a relationship between crosslink density, indicated by X_c, and the network entanglement in the hydrogel, affecting their mechanical stability and performance at the intermediate molecular weights. The measurements of $G'_{\text{amorphous}}$ at higher temperatures (above glass transition, revealing amorphous behavior) provide insights into the polymer networks' behavior under conditions that simulate end-use environments, allowing for a more practical understanding of material performance.

Table S2. Amorphous Storage Moduli versus Molecular Weight

**Sample Name**	M_w **(g/mol)**	$G'_{amorphous}$ **(Pa)**	X_c **(mol/m³)**
700	700	72344	2.70
4k	4,000	5025.6	0.23
8k	8,000	1390.5	0.05
10k	10,000	68907	2.34

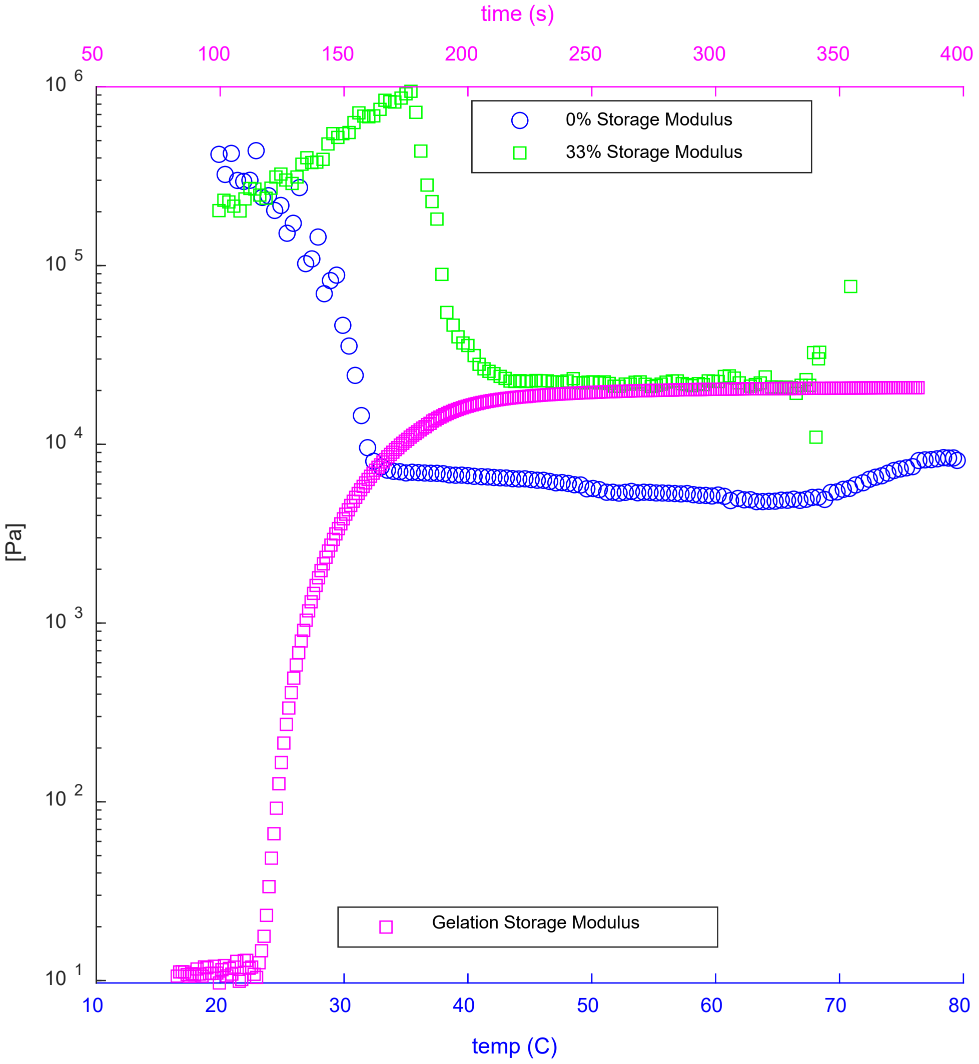

Figure S20. Temperature-dependent storage modulus profiles for PEGDA hydrogels with a molecular weight of 4,000 g/mol at 10% weight concentration. The graph shows three distinct curves: the 0% storage modulus (blue circles) representing the dry state, the 33% storage modulus (green squares) indicating the modulus at 33% swelling, and the gelation storage modulus (pink squares) which captures the modulus during the gelation process over time. Measurements were conducted from 20°C to 70°C, illustrating the changes in hydrogel stiffness as a function of temperature and hydration level. The stability of the hydrogel structure across a range of temperatures can be observed, with the transition points indicating critical temperatures for gelation and material performance under simulated conditions.

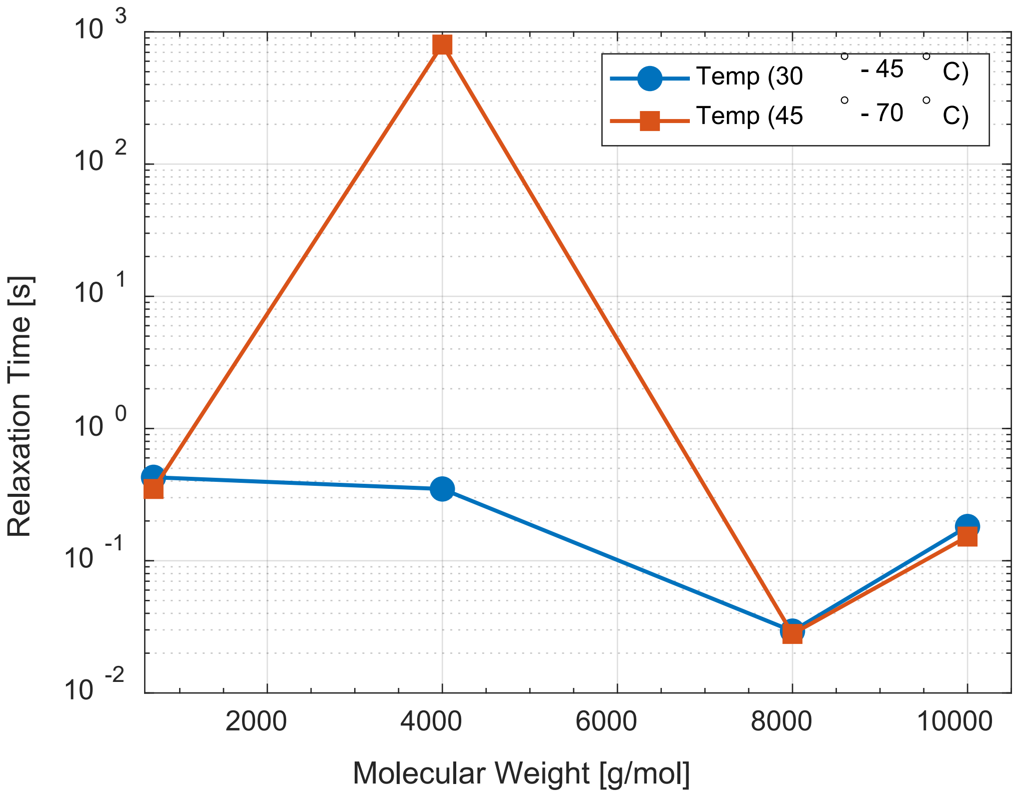

Figure S21. Relaxation time calculated from rheological temperature sweeps at low ranges, 30C - 45C, and high ranges 45C - 70C.

Swelling and Diffusion.

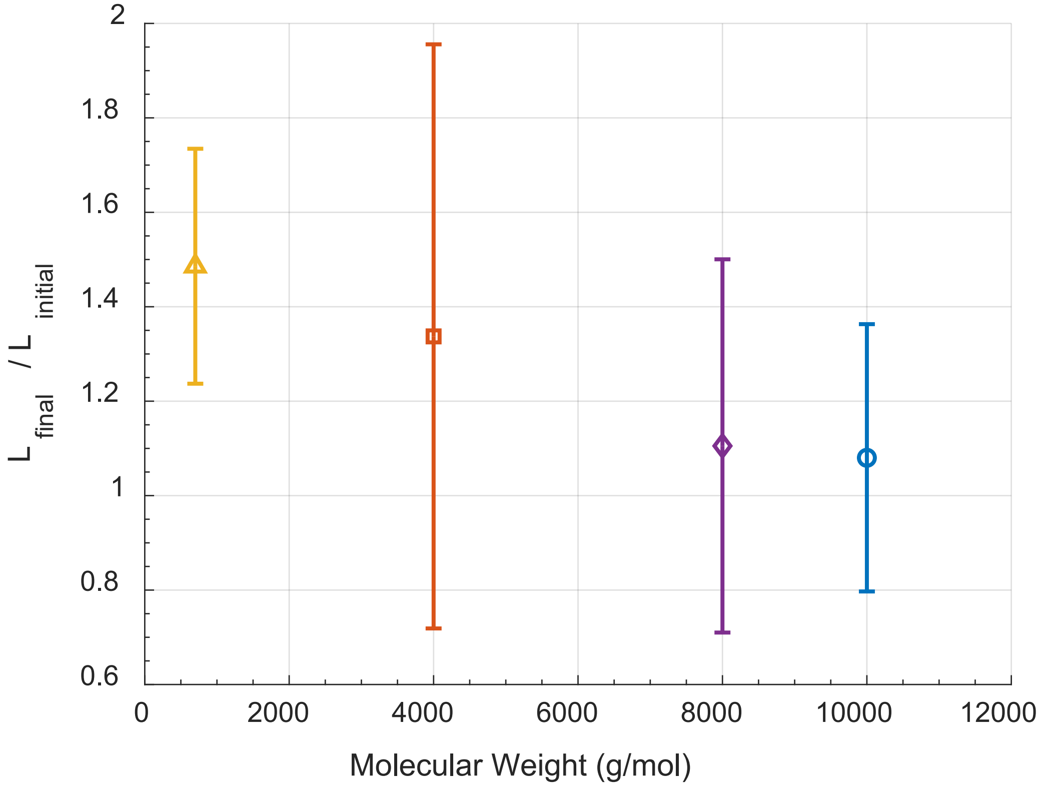

Figure S22. Average sample thickness ratios, L/L_0 versus molecular weight with standard deviations.

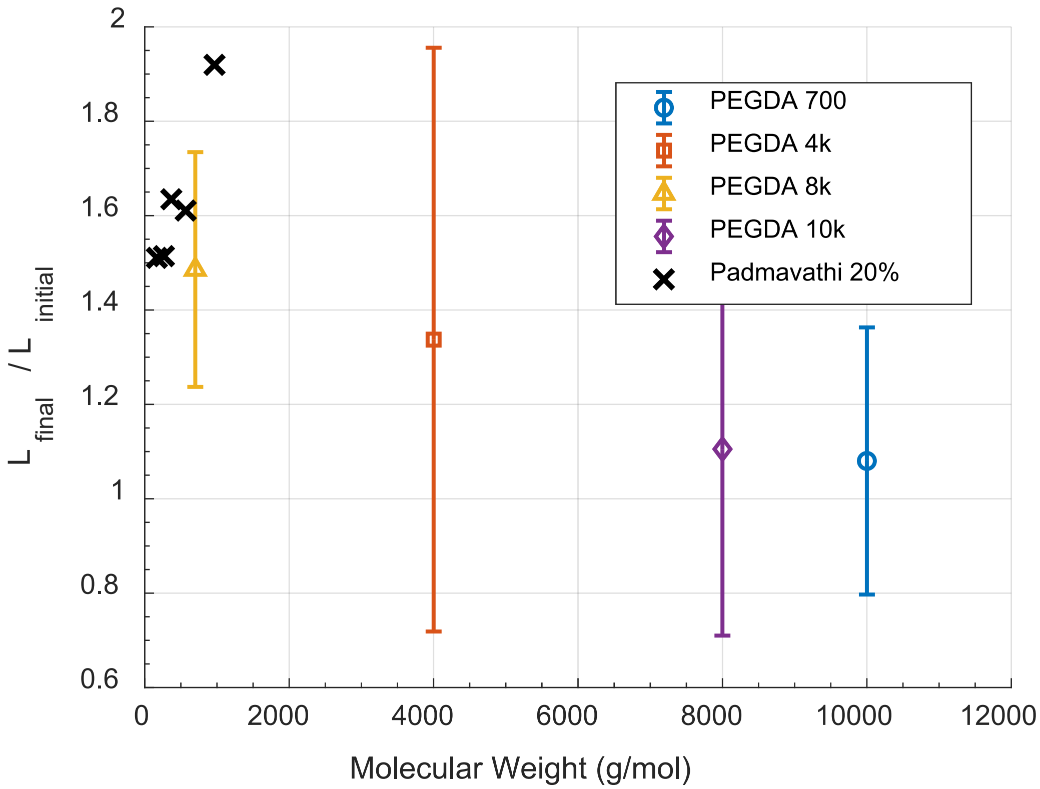

Figure S23. Average sample thickness ratios, L/L_0 versus molecular weight with standard deviations including Padmavathi PEGDA 20% Gel.[Padmavathi, N. C.; Chatterji, P. R. Structural Characteristics and Swelling Behavior of Poly(ethylene glycol) Diacrylate Hydrogels. *Macromolecules* **1996**, *29* (6), 1976-1979. DOI: 10.1021/ma950827r.]

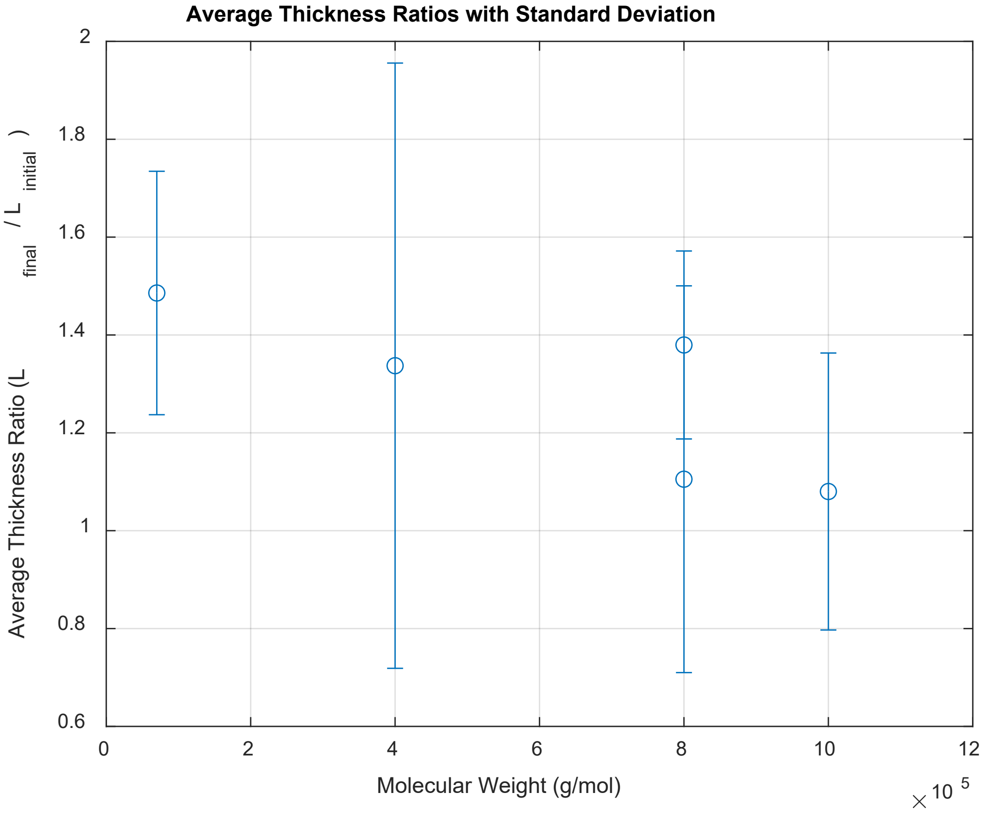

Figure S24. Average sample thickness ratios, L/L_0 versus molecular weight with standard deviations. PEGDA 8k was measured with both 10% and 20% weight gelation concentrations.

Anisotropy Derivation

We quantify the degree of swelling anisotropy by comparing gravimetric swelling results with thickness swelling. The volume fraction of polymer from gravimetric swelling measurements, as calculated using equation 6 of the main manuscript, can be related to the dimensions of a cylindrical sample as follows:

$$\phi_p(w_p) = \frac{V_{dry}}{V_{swollen}} = \frac{L_d \frac{\pi}{4} D_d^2}{L_s \frac{\pi}{4} D_s^2} = \frac{L_d D_d^2}{L_s D_s^2} \quad (S1)$$

Subscript ‘d’ refers to the dry state, and ‘s’ refers to the swollen state. If the sample were to swell isotropically, then the thickness measurement could also be used to calculate a polymer volume fraction as follows:

$$\phi_p(L) = \frac{V_{dry}}{V_{swollen}} = \frac{L_d^3}{L_s^3} \quad (S2)$$

For perfectly isotropic swelling, the ratio of these two polymer volume fractions would be unity.

$$\frac{\phi_p(L)}{\phi_p(w_p)} = \frac{L_d^3/L_s^3}{L_d D_d^2/L_s D_s^2} = \frac{L_d^2/L_s^2}{D_d^2/D_s^2} \quad (S3)$$

Otherwise, the ratio will diverge from a value of one depending on whether the cylindrical sample swells more in the radial or axial (thickness) direction. The square root of the polymer volume fraction ratio gives a direct indication of relative swelling in these two directions as follows:

$$\left[\frac{\phi_p(L)}{\phi_p(w_p)}\right]^{1/2} = \left[\frac{L_d^2/L_s^2}{D_d^2/D_s^2}\right]^{1/2} = \frac{L_d/L_s}{D_d/D_s} \quad (S4)$$

In other words, anisotropy as defined here and in equation 7 in the main text, indicates that radial swelling exceeds axial swelling if the value is greater than one; rearranging the equation makes this clear.

$$\left[\frac{\phi_p(L)}{\phi_p(w_p)}\right]^{1/2} = \frac{D_s/D_d}{L_s/L_d} \quad (S5)$$

The denominator of equation S5 is directly measured in drop indicator experiments, while the numerator is inferred from gravimetric experiments using the assumption of volume additivity,

i.e. that the density of the mixture is the weighted average of the pure component densities or that there is no volume change upon mixing.

Method for Determining the Apparent Diffusion Coefficient

- **Method for Determining the Apparent Diffusion Coefficient Using Modified Non-Dimensionalized Mass**
- **Step 1: Data Collection**
- **Sample Preparation:** Polyethylene glycol diacrylate (PEGDA) hydrogels with varying molecular weights (MWs) were prepared.
- **Swelling Experiment:** The hydrogels were subjected to a swelling experiment, and the change in thickness over time was recorded.
- **Data Logging:** The recorded thickness measurements were stored in designated file paths for subsequent analysis.
- **Step 2: Data Preprocessing**
- **File Organization:** All data files were stored in a structured directory, organized based on the molecular weight of the PEGDA samples.
- **File Selection:** Specific files corresponding to different PEGDA molecular weights (700, 4k, 8k, 10k) were selected for analysis to ensure that relevant data was used.
- **Step 3: Data Processing**
- **Dimensionalization:** The measured thickness data was converted to absolute thickness using the initial thickness of each sample. The absolute thickness values were then used to calculate the volume of the hydrogel samples, assuming a cylindrical geometry.
- **Mass Calculation:** The mass of the hydrogel samples was calculated using the density of PEG (1.09 g/cm^3). This step involved converting the computed volume to mass by multiplying it by the density.
- **Modified Non-Dimensionalization:** The mass was then non-dimensionalized by normalizing it with respect to the initial mass. This step allows for a comparison of the swelling behavior across different samples.
- **Step 4: Data Analysis**
- **Section Selection:** A specific time section was selected for each PEGDA sample to focus on the most relevant swelling behavior. The analysis was limited to the chosen time range to calculate the diffusion coefficient accurately.
- **Linear Fit:** A linear fit was performed on the selected time section of the modified non-dimensionalized mass data. The slope of this linear fit was determined.
- **Calculation of Diffusion Coefficient:** The apparent diffusion coefficient (D) was calculated using the formula:

$$\frac{M_t}{M_\infty} \approx \frac{4}{\sqrt{\pi}} \sqrt{\frac{Dt}{L^2}} \quad (S6)$$

where the thickness at the end of the experimental swelling segment is L.

- **Tabulate Results**: The molecular weight, date of the experiment, and calculated diffusion coefficient for each sample were recorded in a table.

Step 7: Statistical Analysis

- **Group by Molecular Weight**: The diffusion coefficients were grouped by the molecular weight of the PEGDA hydrogels.
- **Compute Averages and Standard Deviations**: For each molecular weight group, the average diffusion coefficient and its standard deviation were calculated to summarize the data.
- **Display Averages**: The results, including the average diffusion coefficients and standard deviations for each molecular weight group, were presented in a separate table.

In order to apply the numerical model, an approximate diffusion coefficient is needed and was determined by applying Crank's early time approximation for one-dimensional diffusion in a plane sheet.[2, 3]

$$\frac{M_s}{M_{s,f}} \approx \frac{4}{\sqrt{\pi}}\sqrt{\frac{Dt}{L^2}} \quad (S7)$$

where M_s is the mass of solvent absorbed at time t, $M_{s,f}$ is the equilibrium solvent mass at infinite time, D is the apparent diffusion coefficient, and L is the thickness of the sample. The apparent diffusion coefficient values for Figure 6 data are reported in Table S3.

Table S3. Apparent diffusion coefficients, D_{app}, and corresponding parameters for PEGDA samples of different molecular weights over specified time sections. Each sample is labeled with a letter corresponding to its Figure 6 panel. Values for n and k are mass transfer coefficients of the experimental data for each sample.

**Symbol (units)**	$\boldsymbol{D_{app}}\left(\frac{cm^2}{s}\right)$	**Time Section (s)**	**n**	**k** (s^{-n})
PEGDA 700 (a)	1.63×10^{-8}	1001 to 2000	1.74	9.25×10^{-7}
PEGDA 4k (b)	2.43×10^{-12}	1857 to 2320	1.01	2.89×10^{-5}
PEGDA 8k (c)	8.45×10^{-9}	88 to 115	1.63	1.68×10^{-4}
PEGDA 10k (d)	1.01×10^{-11}	1 to 1137	4.13	6.01×10^{-11}

The diffusion coefficients of the various samples and measurements have been averaged to arrive at diffusion coefficients listed in Table S4:

Table S4. Apparent diffusion coefficients by sample.

Sample Name	Tau (s)	Diffusion Coefficient
700 15% 10/28/2023 5:08:41:PM	1.03e+03	1.12e-09
700 15% 9/29/2023 5:43:05:PM	8.90e+03	9.23e-12
700 15% 9/5/2023 12:33:02:PM	5.57e+03	9.32e-12
700 15% 1/20/2024 11:45:24:AM	1.91e+03	2.37e-11
700 15% 1/27/2024 11:23:35:AM	2.55e+03	3.85e-10
700 15% 1/29/2024 5:52:21:PM	9.15e+03	4.32e-12
700 15% 2/26/2024 6:00:12:PM	3.92e+03	6.11e-11
700 15% 6/17/2024 5:45:45:PM	9.25e+03	7.33e-12
4 15% 10/28/2023 5:33:26:PM	3.49e+05	3.49e-19
4 15% 2/17/2024 11:24:56:AM	3.61e+03	0.00e+00
4 15% 2/29/2024 12:56:27:PM	1.33e+05	2.29e-18
4 15% 2/8/2024 1:17:02:PM	6.21e+03	2.09e-12
4 15% 3/23/2024 12:34:35:PM	2.23e+04	0.00e+00
4 15% 4/24/2024 5:34:20:PM	8.19e+03	1.57e-13
4 15% 5/17/2024 3:10:54:PM	1.83e+04	5.87e-13
8 15% 5/16/2023 12:15:57:PM	4.56e+04	4.04e-14
8 15% 10/17/2023 2:37:03:PM	1.23e+03	3.06e-10
8 15% 10/25/2023 6:50:05:PM	7.72e+03	1.42e-12
8 15% 10/30/2023 5:46:20:PM	1.88e+20	0.00e+00
8 15% 2/1/2024 1:45:37:PM	3.16e+04	1.72e-13
8 15% 3/6/2024 6:00:55:PM	4.90e+04	3.15e-15
8 15% 10/19/2023 1:23:33:PM	7.94e+03	1.98e-14
10 15% 10/23/2023 11:16:59:AM	5.51e+04	7.51e-13
10 15% 10/6/2023 4:45:59:PM	2.72e+03	6.96e-10
10 15% 11/21/2023 1:19:58:PM	-1.66e+20	0.00e+00
10 15% 2/19/2024 5:39:00:PM	-3.95e+20	0.00e+00
10 15% 2/7/2024 5:46:55:PM	1.28e+04	1.07e-13
10 15% 3/23/2024 12:47:06:PM	-9.05e+04	1.69e-16
10 15% 3/28/2024 1:06:18:PM	1.49e+04	4.31e-13
10 15% 3/2/2024 2:52:32:PM	5.48e+03	4.19e-12
10 15% 3/6/2024 5:57:58:PM	1.19e+04	1.59e-14
10 15% 4/15/2024 5:41:08:PM	3.00e+04	2.94e-15
10 15% PEGDA_10k_6_25_2024 vs 1:length(PEGDA_10k_6_25_2024)	1.25e+04	1.04e-12

Figure S25 presents the linearized dimensionless swelling for the 700 g/mol PEGDA hydrogel. The experimental data is compared with the analytical solutions for various diffusion coefficients. The analytical solutions, represented by the colored lines, closely match the experimental data, demonstrating the validity of the analytical approach for this molecular weight. Figure S26 depicts the dimensionless linearized swelling for the 4000 g/mol PEGDA hydrogel. Both numerical and analytical solutions are plotted against the experimental data. The numerical solutions, represented by the colored lines, show a closer fit to the experimental data compared to the analytical solutions, highlighting the necessity of numerical methods for accurately predicting swelling behavior at higher molecular weights. Figure S27 shows the dimensionless linearized swelling for the 8000 g/mol PEGDA hydrogel. The experimental data is compared with both numerical and analytical solutions. Similar to the 4000 g/mol case, the numerical solutions provide a better fit to the experimental data, indicating that numerical methods are essential for capturing the swelling kinetics of higher molecular weight PEGDA hydrogels. Figure S28 presents the dimensionless linearized swelling for the 10000 g/mol PEGDA hydrogel. The experimental data is compared with the numerical solutions, which accurately capture the swelling behavior. The close match between the numerical solutions and the experimental data confirms the reliability of the model for high molecular weight hydrogels.

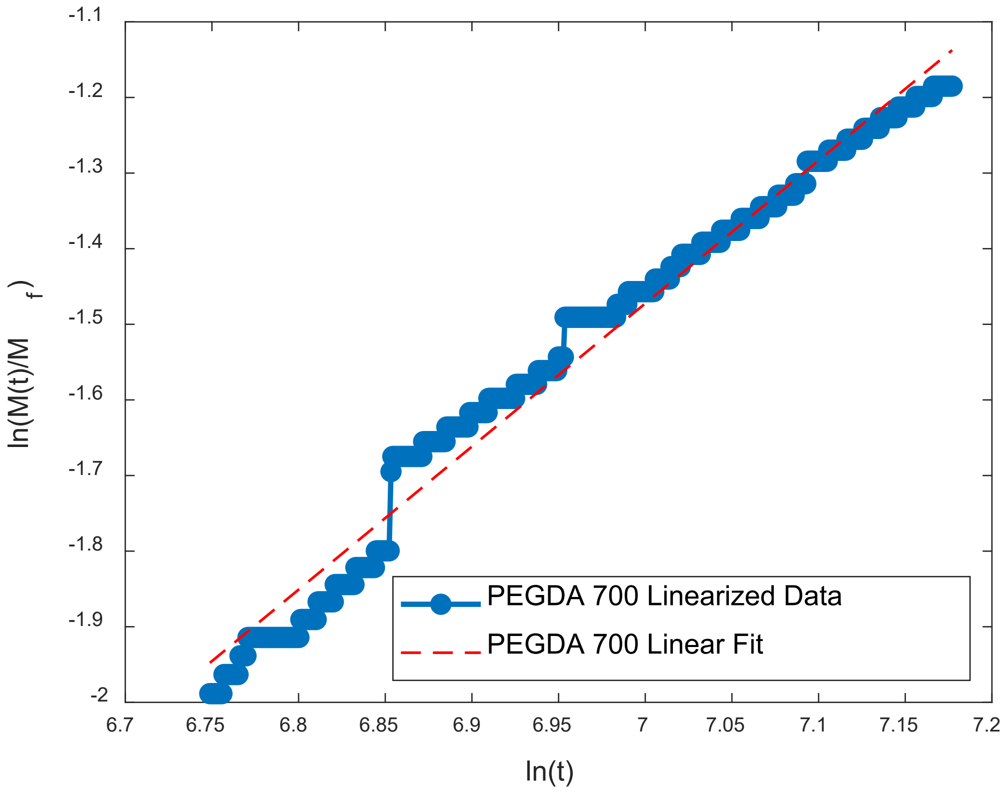

Figure S25. Linearized plot for PEGDA 700 Gel from 850s to 2000s.

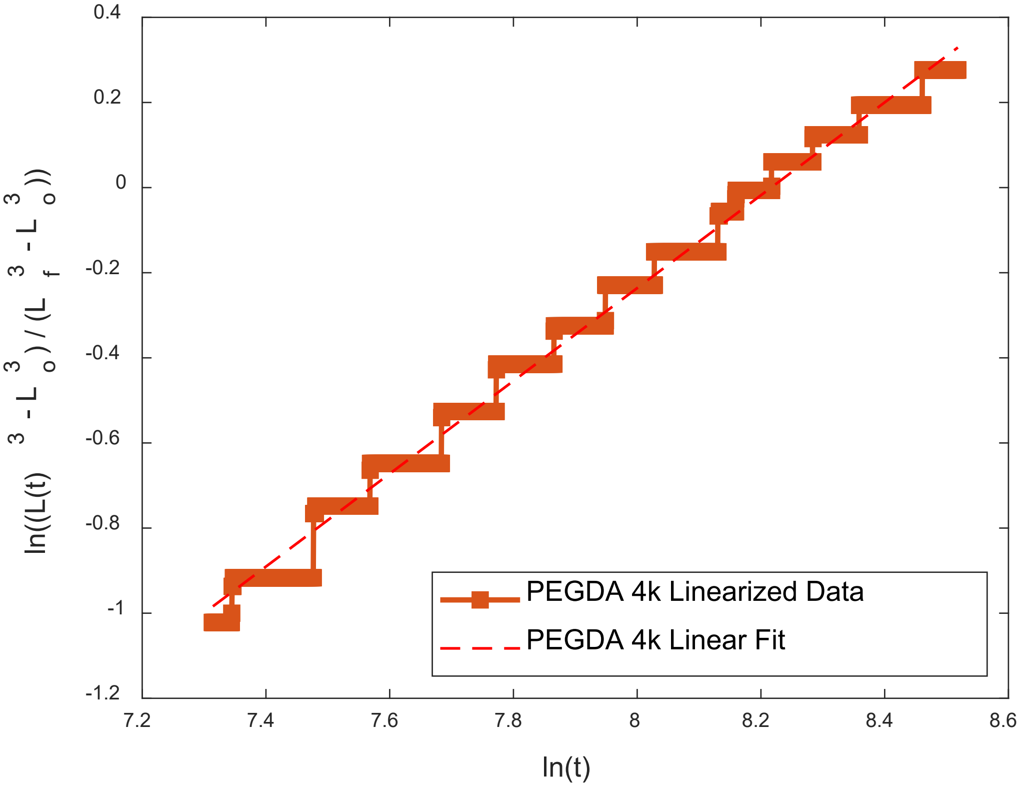

Figure S26. Linearized plot for PEGDA 4k Gel from 1500 s to 5000 s.

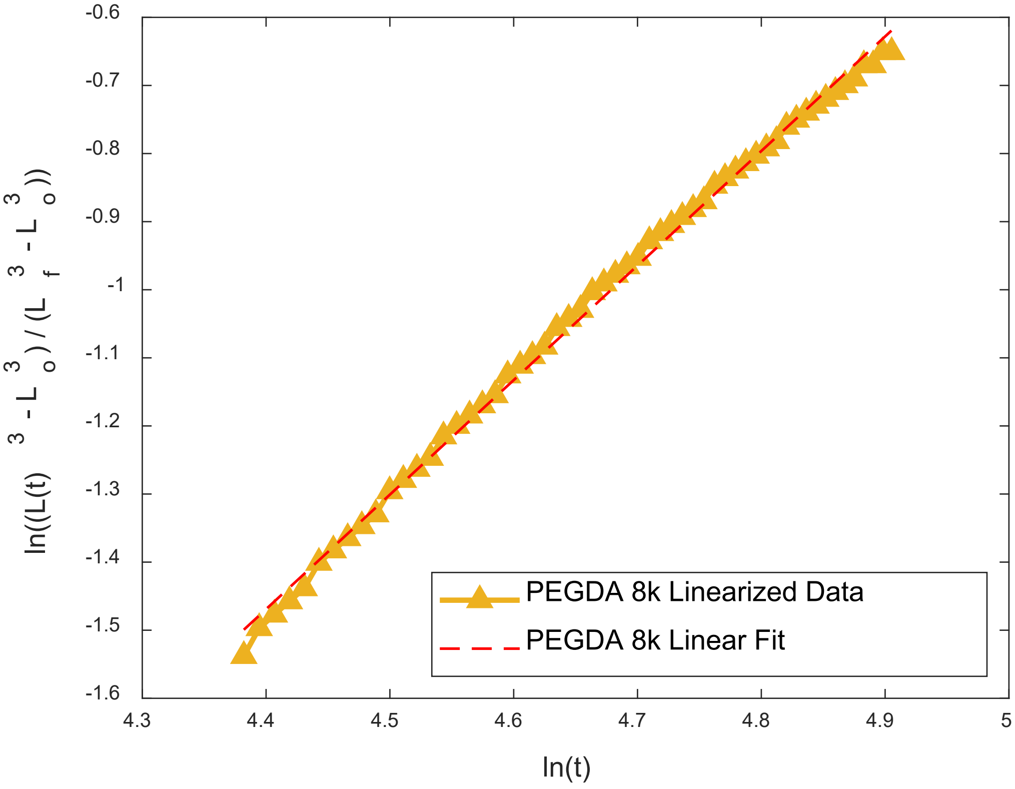

Figure S27. Linearized plot for PEGDA 8k Gel from 80 s to 135 s.

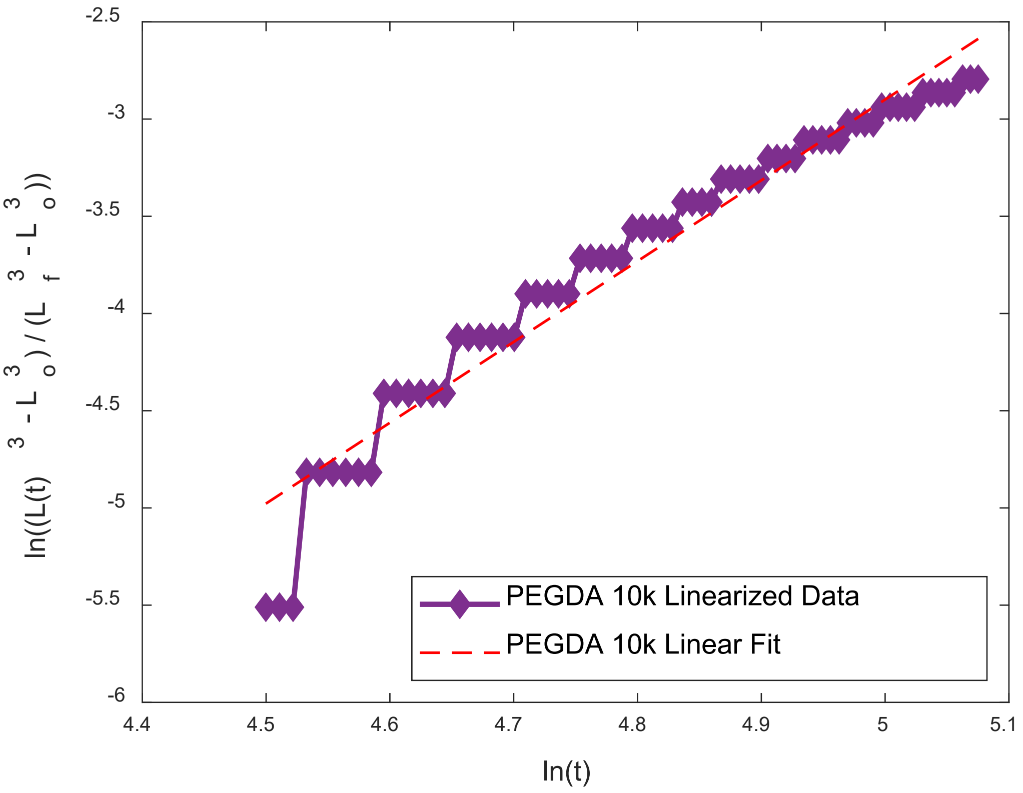

Figure S28. Linearized plot for PEGDA 10k Gel from 90 s to 160 s.

To further understand the initial swelling behavior, we also analyzed the time required for the hydrogels to reach 5% and 90% of their equilibrium swelling. This metric provides insights into the early and late stages of swelling and helps compare the initial swelling rates across different molecular weights. The initial swelling rate is crucial for applications where rapid response is necessary.

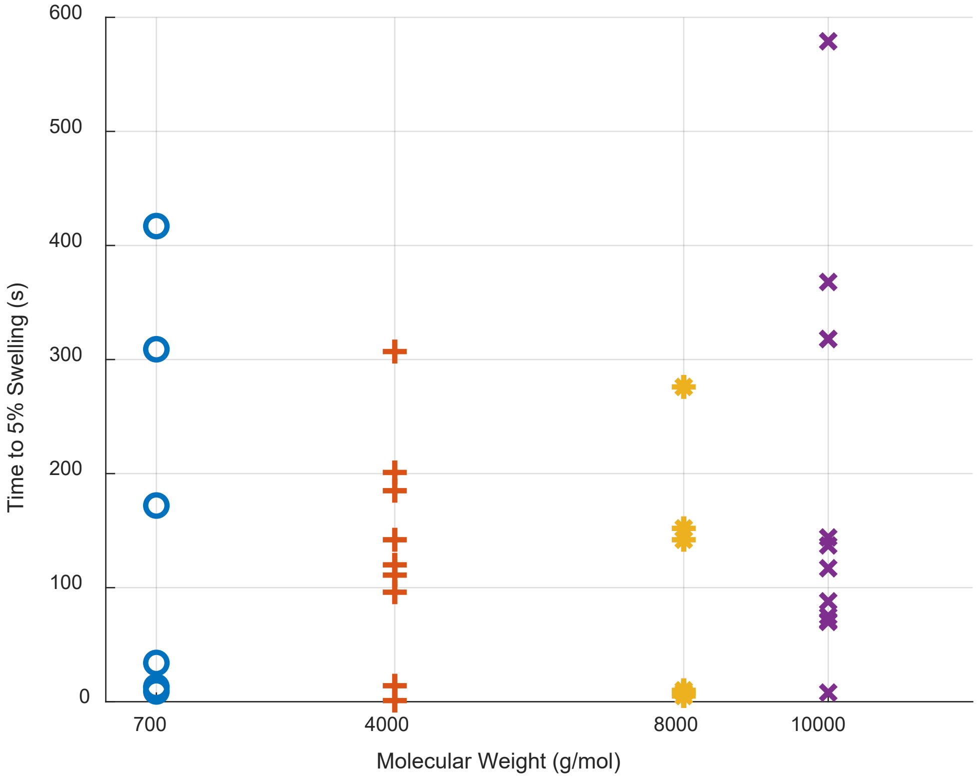

Figure S29. Time to reach 5% swelling for PEGDA hydrogels with varying molecular weights (700, 4000, 8000, and 10000 g/mol). Each point represents a measurement of time (in seconds) taken for the hydrogel to achieve 5% of its equilibrium swelling. The data shows that higher molecular weight PEGDA hydrogels generally require longer times to reach 5% swelling, indicating a slower initial swelling rate compared to lower molecular weight hydrogels.

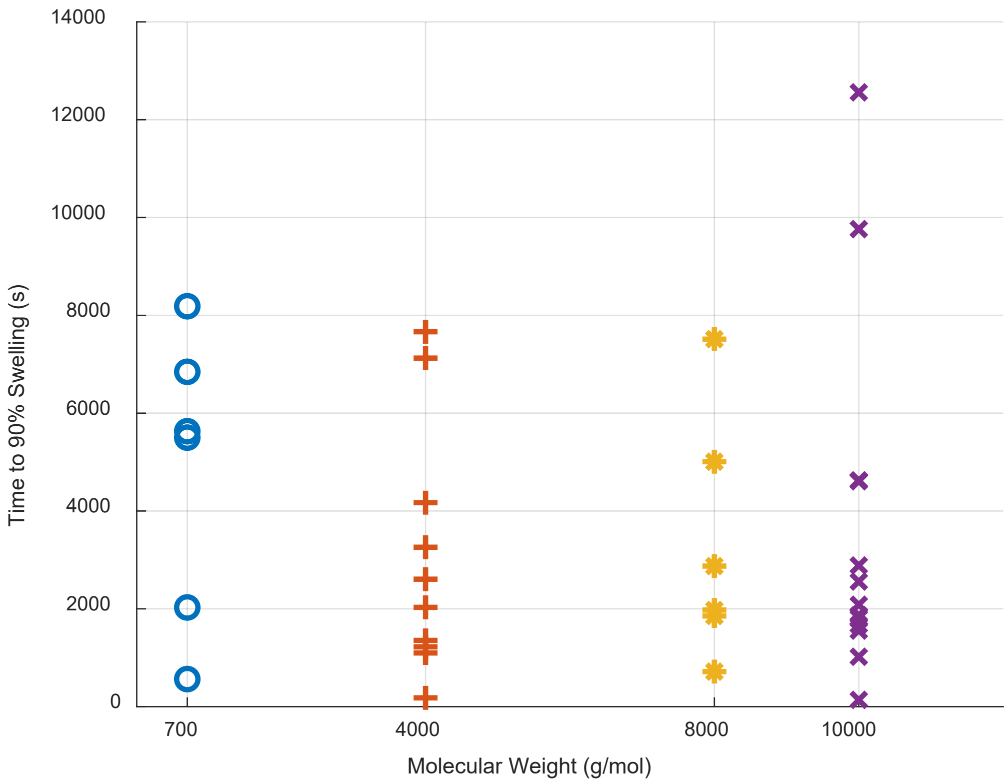

Figure S30. Time to reach 90% swelling for PEGDA hydrogels with varying molecular weights (700, 4000, 8000, and 10000 g/mol). Each point represents a measurement of time (in seconds) taken for the hydrogel to achieve 90% of its equilibrium swelling. The data shows that higher molecular weight PEGDA hydrogels generally require longer times to reach 90% swelling, indicating a slower swelling rate compared to lower molecular weight hydrogels.

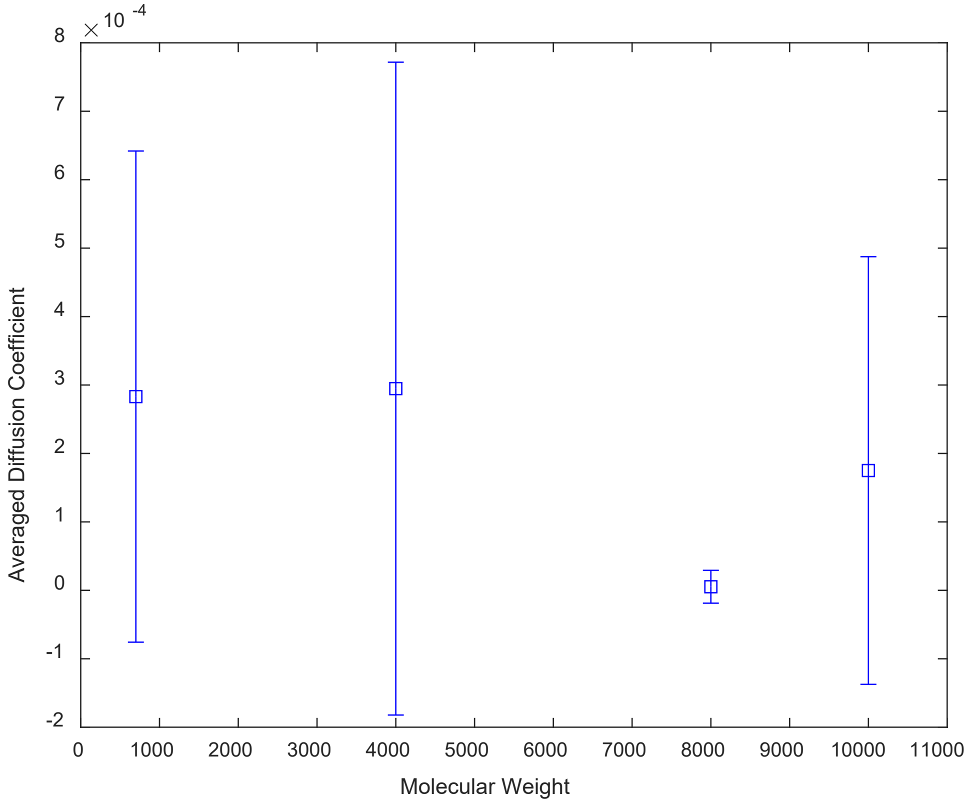

Figure S31. Averaged diffusion coefficient as a function of molecular weight for all PEGDA samples measured.

The data presented in Figure S31 illustrate the averaged diffusion coefficient as a function of molecular weight for all PEGDA samples measured. The diffusion coefficients exhibit a broad range of values, with significant variability indicated by the large error bars. This suggests that the molecular weight, UV exposure time, and a host of other factors like temperature, barometric pressure, and relative humidity of PEGDA polymers play a substantial role in determining their diffusion characteristics. The observed trend implies that higher molecular weight polymer-solvent systems may have reduced diffusion coefficients for the solvent, which is consistent with literature on larger polymer chain hydrogels[4]. The solvent used in all these experiments for Figure S31 is DI water. The results suggest that larger polymers have more entanglements and a

higher molecular weight between the crosslinks, which hinder diffusion and allow larger volume ratios respectively. The ethylene oxide backbone of the PEGDA polymers attracts polar water molecules at the atomic level, facilitating the diffusion process[5]. However, the increased entanglement of larger polymers may hinder diffusion, resulting in lower diffusion coefficients. Additionally, the variability in the diffusion coefficients could be attributed to the variability in UV curing of the polymeric hydrogels, which may affect the consistency of the volumetric swelling measurements[5]. The use of a Mitutoyo drop indicator for measuring thickness changes over time appears effective, but the large error margins indicate that further refinement of this method might be necessary to reduce measurement uncertainties and obtain more precise data on hydrogel swelling behavior. The data indicate that molecular weight significantly impacts the diffusion behavior of PEGDA in DI water, highlighting the importance of polymer structure in determining hydrogel properties.

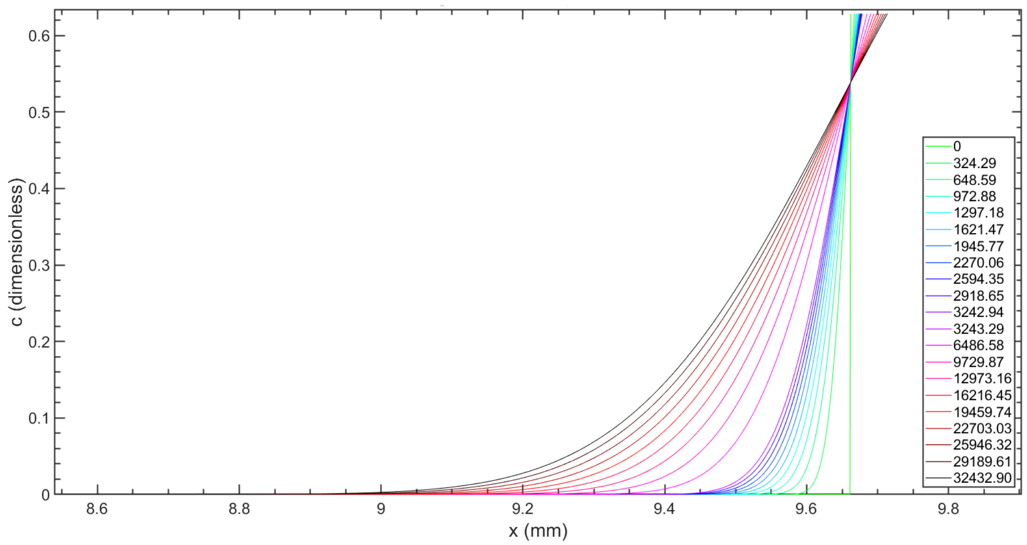

Figure S32. Numerical solution to the 1 dimensional diffusion problem with no voltage applied to the 700 g/mol PEGDA hydrogel swollen with water here shown as a concentration profile where the outer right edge of the polymer is initially dry and begins to be infiltrated with solvent as time passes.

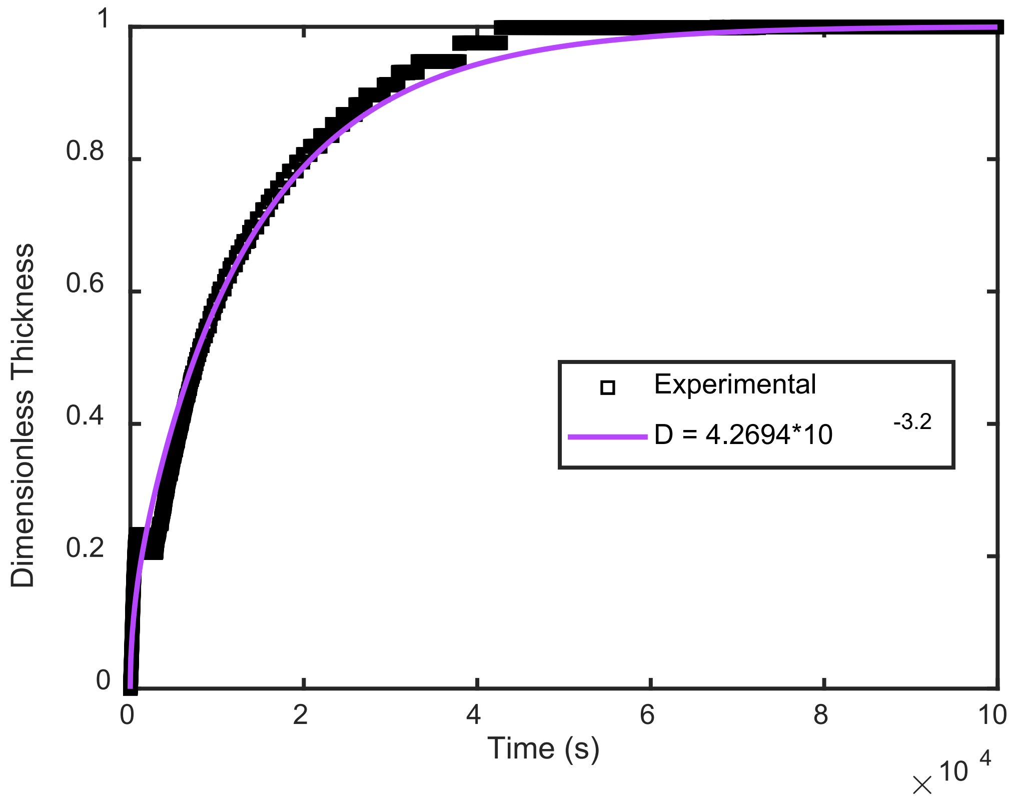

Figure S33. 4000 g/mol PEGDA gel numerical solution to swelling model prediction plotted with experimental swelling data.

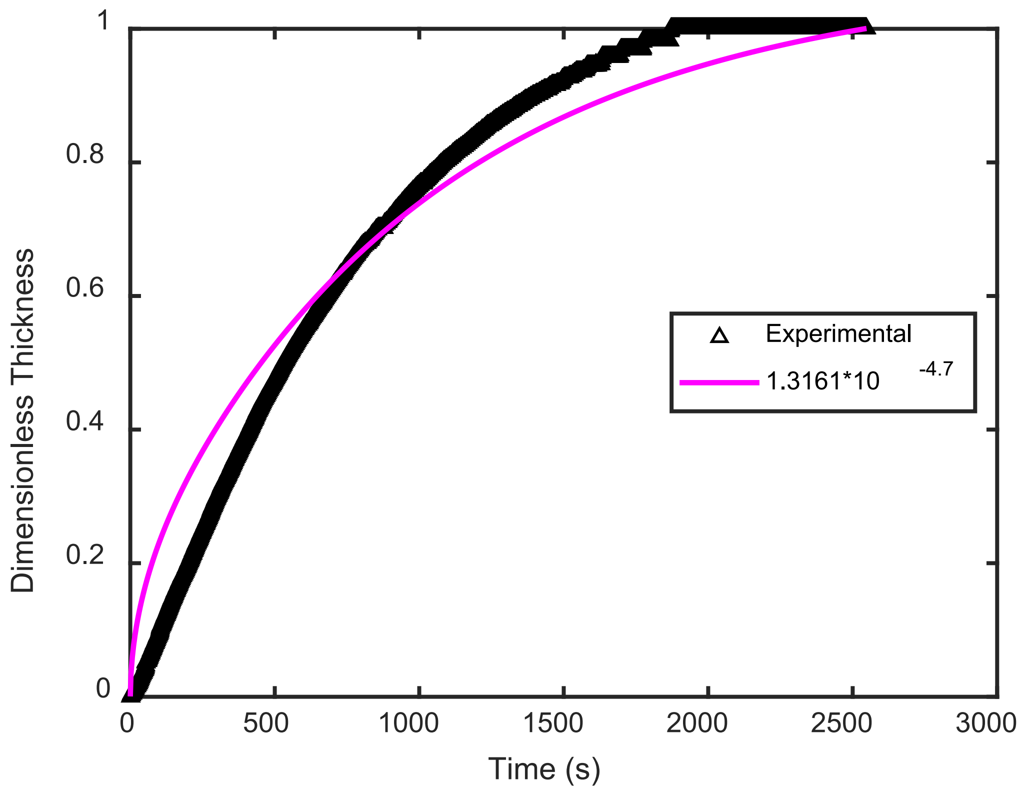

Figure S34. 8000 g/mol PEGDA gel numerical solution to swelling model prediction plotted with experimental swelling data.

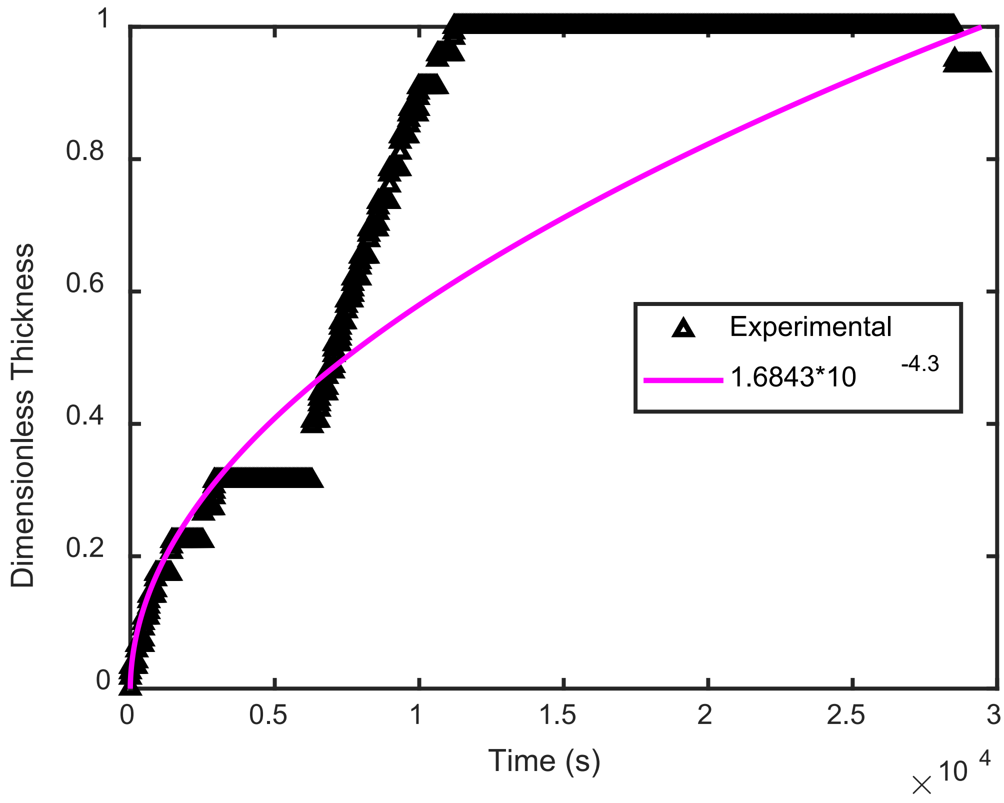

Figure S35. 10000 g/mol PEGDA gel numerical solution to swelling model prediction plotted with experimental swelling data.

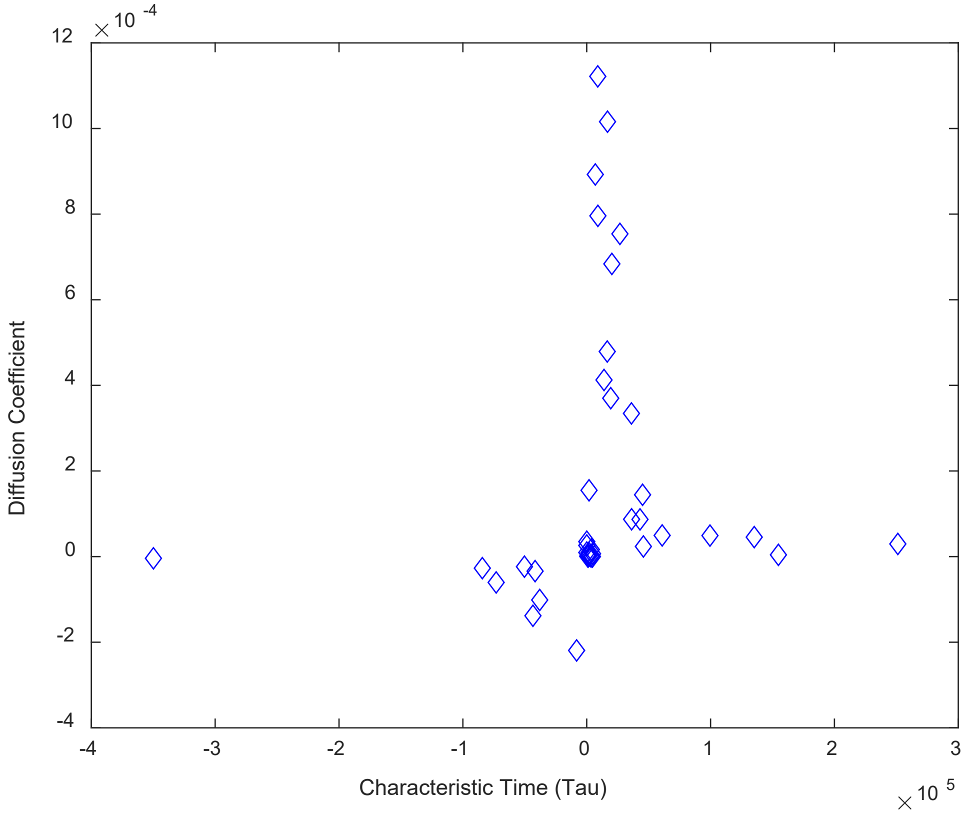

Figure S36. Analysis of quality of swelling experimental data showing samples with deswell that are removed from analysis because of negative diffusion coefficient values with negative characteristic times. These negative diffusion coefficients show deswell for a minority of sample and a majority of diffusion coefficients at a value on the order of 10^{-7}.

The rate was determined based on the slope during a specific section of the final swelling time, which was selected to be equivalent to 20 time intervals. This particular 1/20th segment of the overall time was utilized to calculate the diffusion coefficient as it transitioned from high to low. In Figure S36, the diffusion coefficients for the 700 g/mol, 15% sample are presented. Notably, the diffusion coefficient decreases as swelling increases until the polymer chains reach their maximum stretch. At this equilibrium between polymer and solvent, the experiment w stopped.

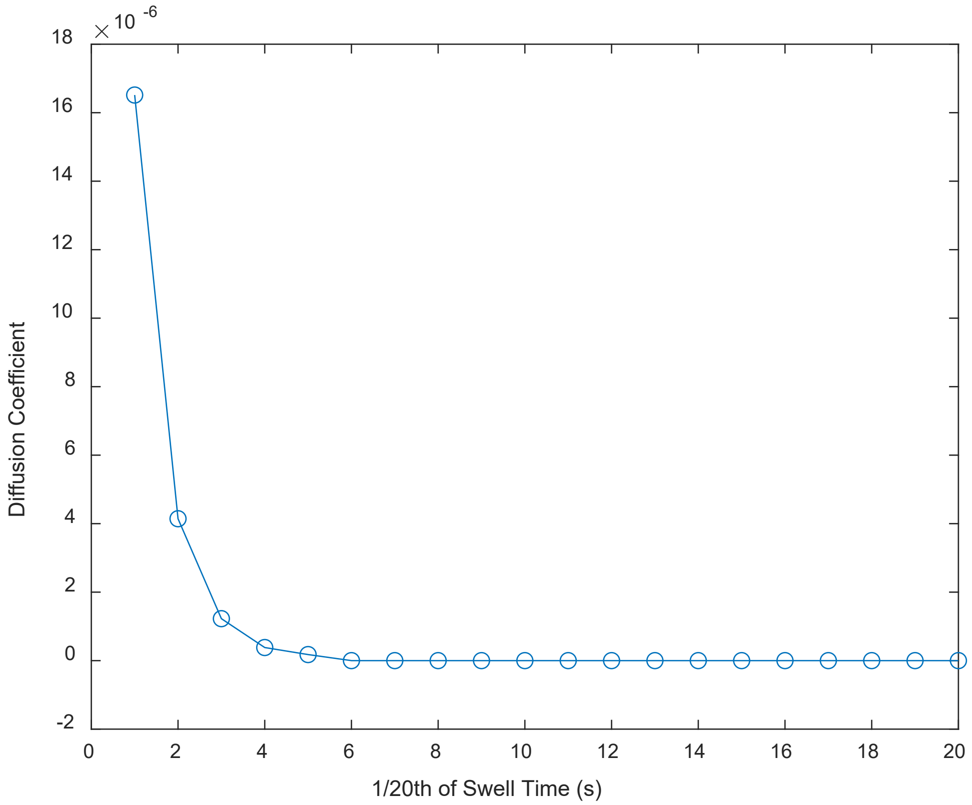

Figure S37. Diffusion coefficient of a swelling experiment on 700 g/mol, 15% PEGDA Gel. The diffusion coefficient drops as swelling increases until the polymer chains are stretched to a maximum.

In order to extract a diffusion coefficient consistent with common practice, PEGDA gel samples were swollen until no thickness change was measured while DI water was present and in contact with the surfaces of the sample discs. The entire swelling dataset was trimmed to 1/20th of the final time to capture the initial swelling rate.

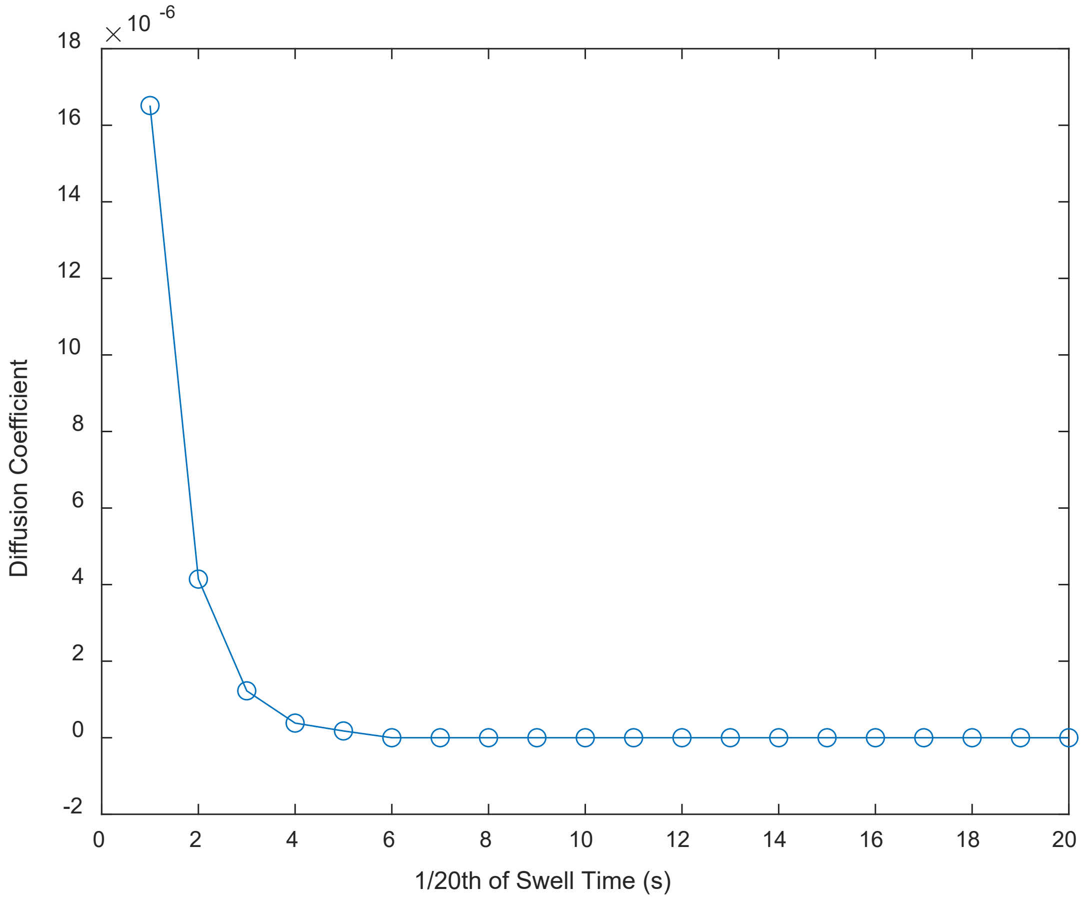

Figure S38. Diffusion Coefficient vs Section for 700g/mol 15% 10/28/23 plotted in linear scale (left) and log scale (right).

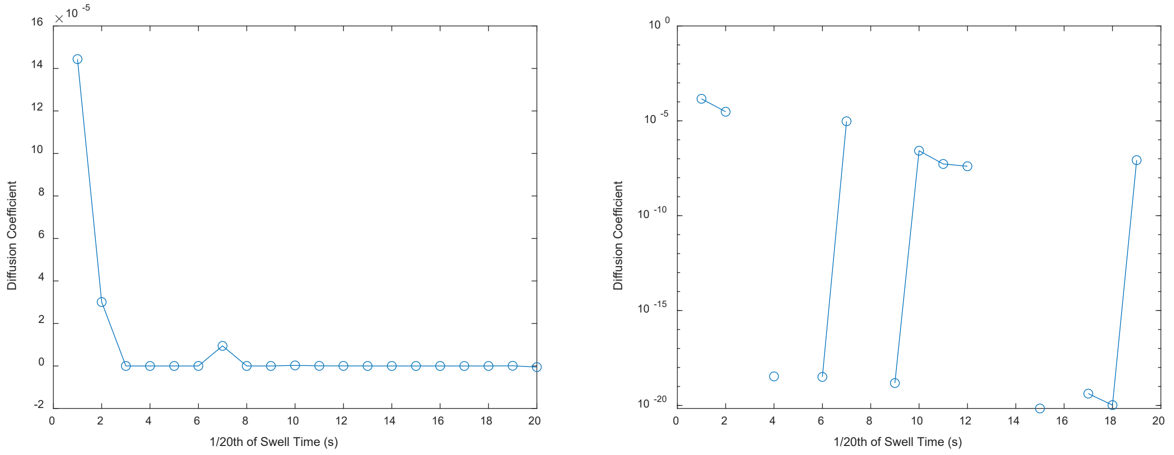

Figure S39. Diffusion Coefficient vs Section for 700 g/mol 15% 02/26/24 plotted in linear scale (left) and log scale (right).

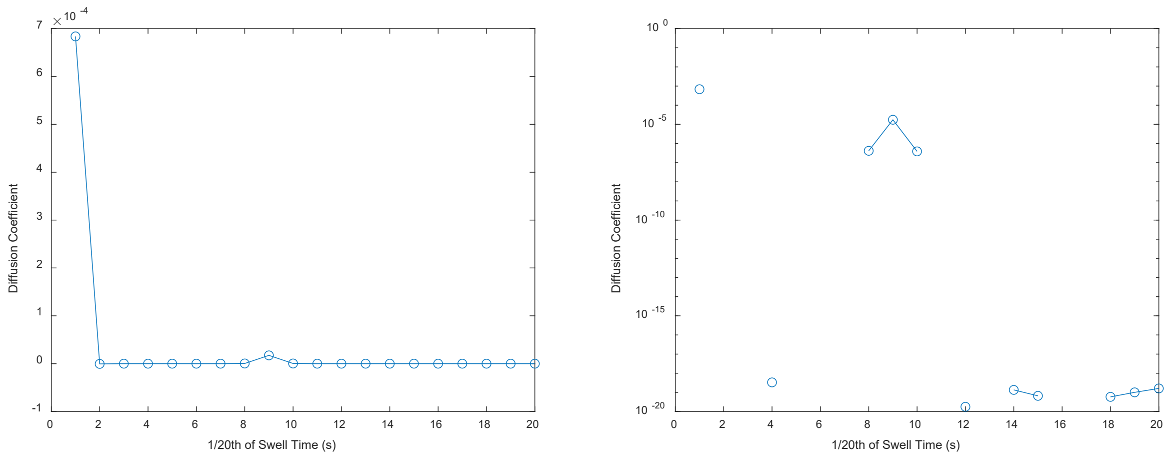

Figure S40. Diffusion Coefficient vs Section for 700 g/mol 15% 01/29/24 plotted in linear scale (left) and log scale (right).

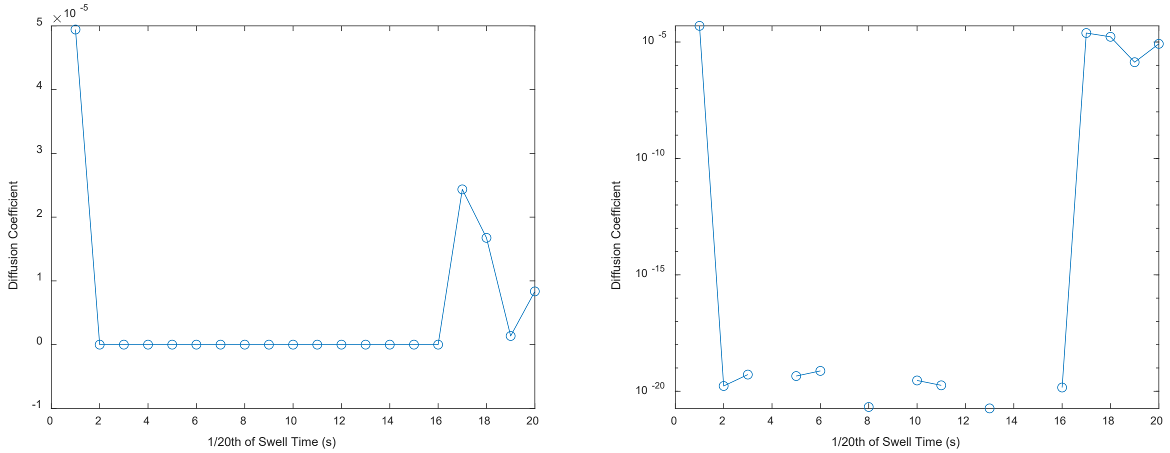

Figure S41. Diffusion Coefficient vs Section for 700 g/mol 15% 01/27/24 plotted in linear scale (left) and log scale (right).

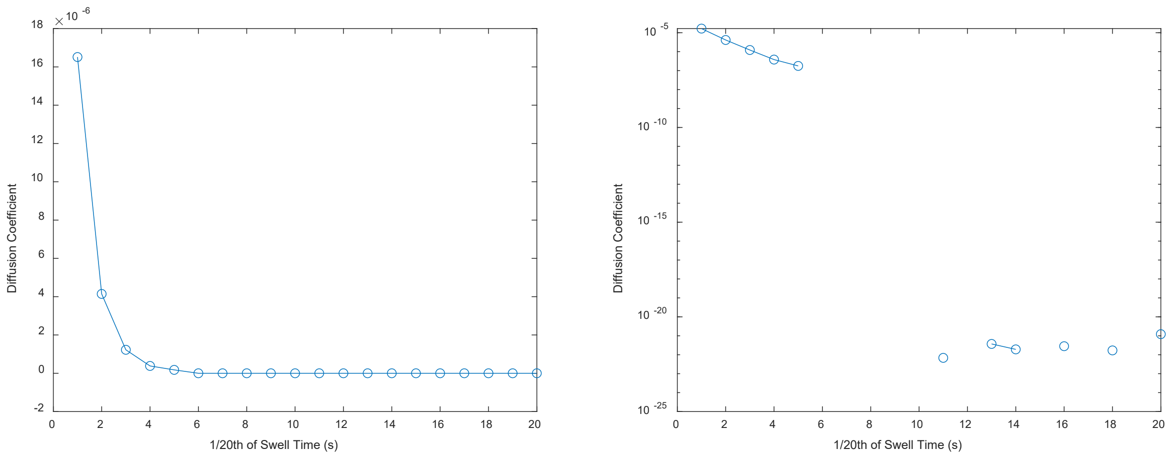

Figure S42. Diffusion Coefficient vs Section for 700g/mol 15% 10/28/23 plotted in linear scale (left) and log scale (right).

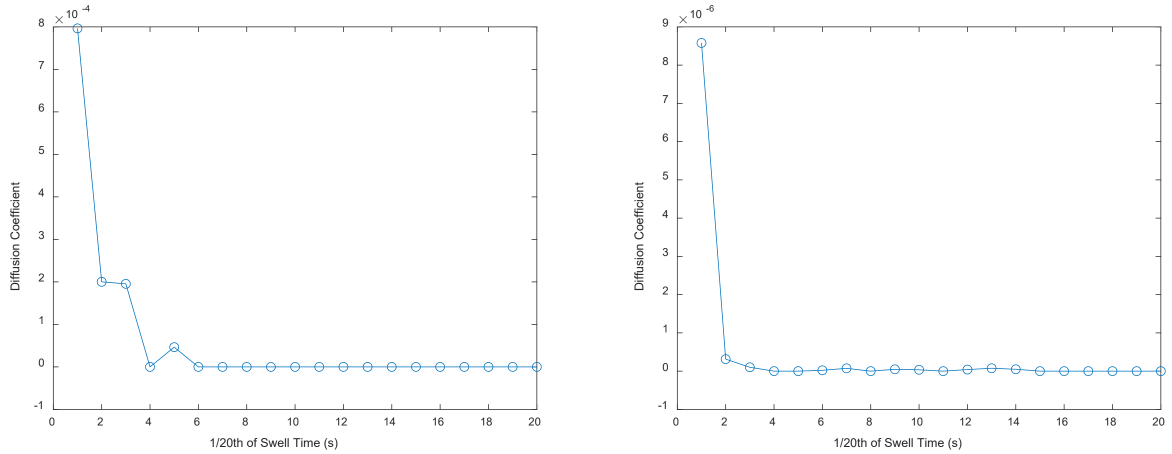

Figure S43. Diffusion Coefficient vs Section for 700 15% 1/20/24 plotted in linear scale (left) and Diffusion Coefficient vs Section for 700 15% 9/29/23 (right.)

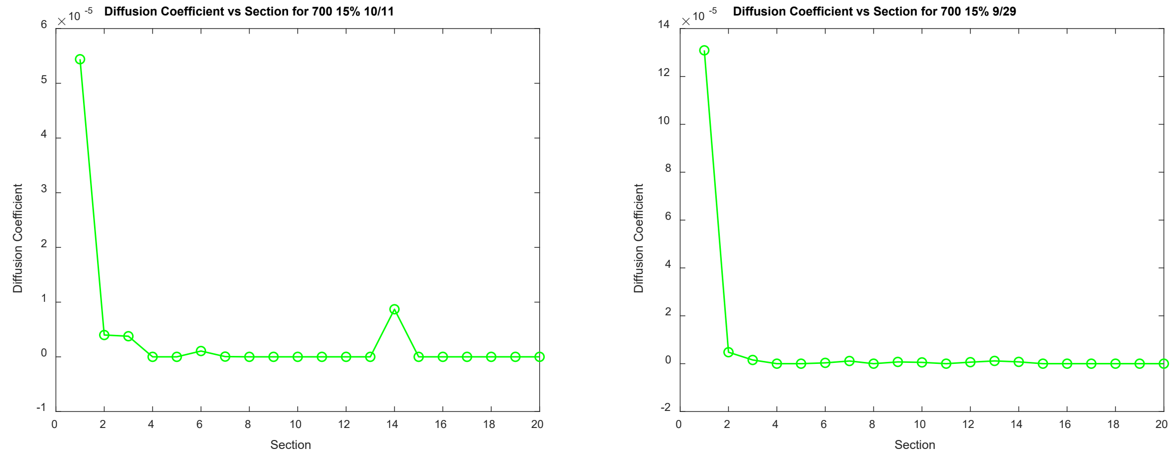

Figure S44. Diffusion coefficient versus section of swelling divided into 20 equal sections for individual experiments on the 700 g/mol M.W. samples.

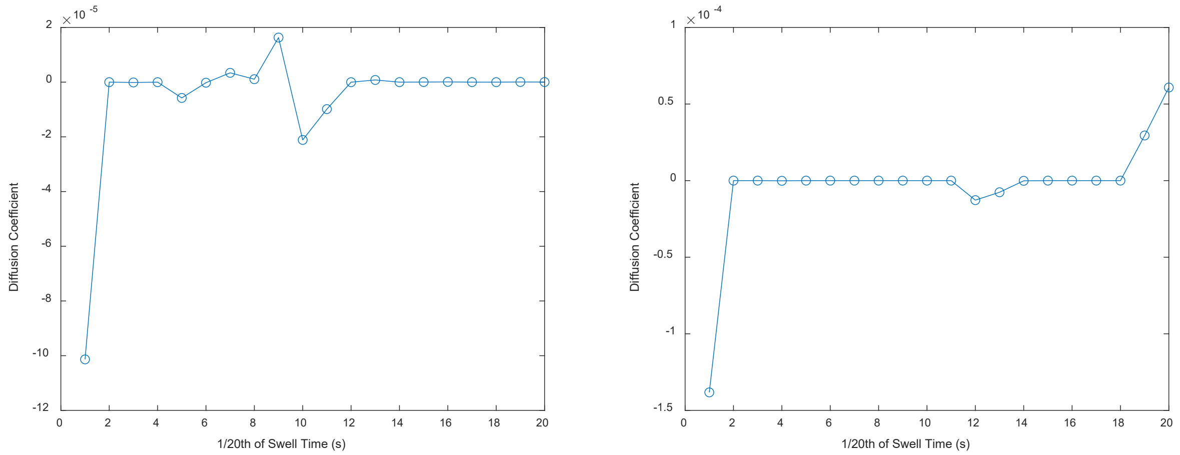

Figure S45. Diffusion Coefficient vs Section for 4000 10% 04/03/24 plotted in linear scale (left) and Diffusion Coefficient vs Section for 4000 10% 03/23/24 (right).

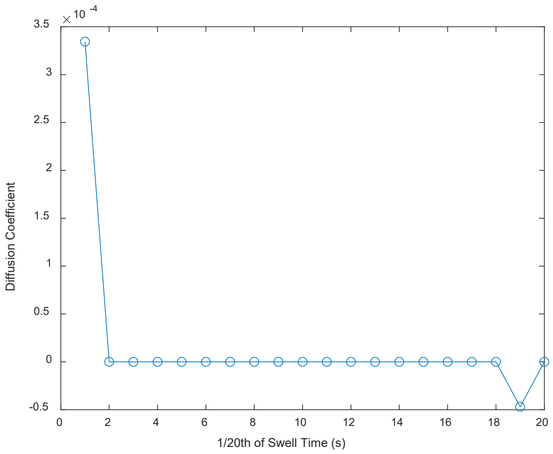

Figure S46. Diffusion Coefficient vs Section for 4000 10% 02/29/24 plotted in linear scale.

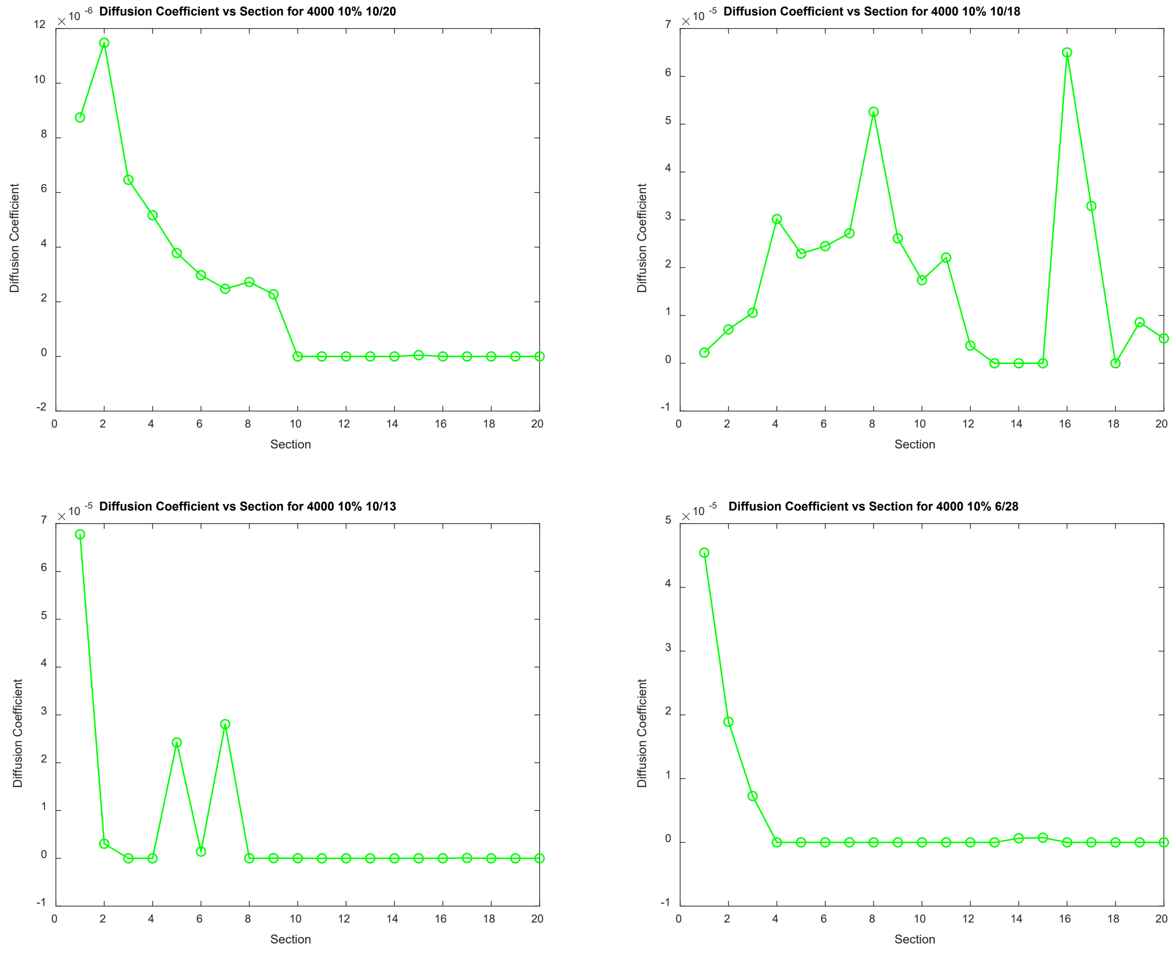

Figure S47. Diffusion coefficient versus section of swelling divided into 20 equal sections for individual experiments on the 4000 g/mol M.W. samples.

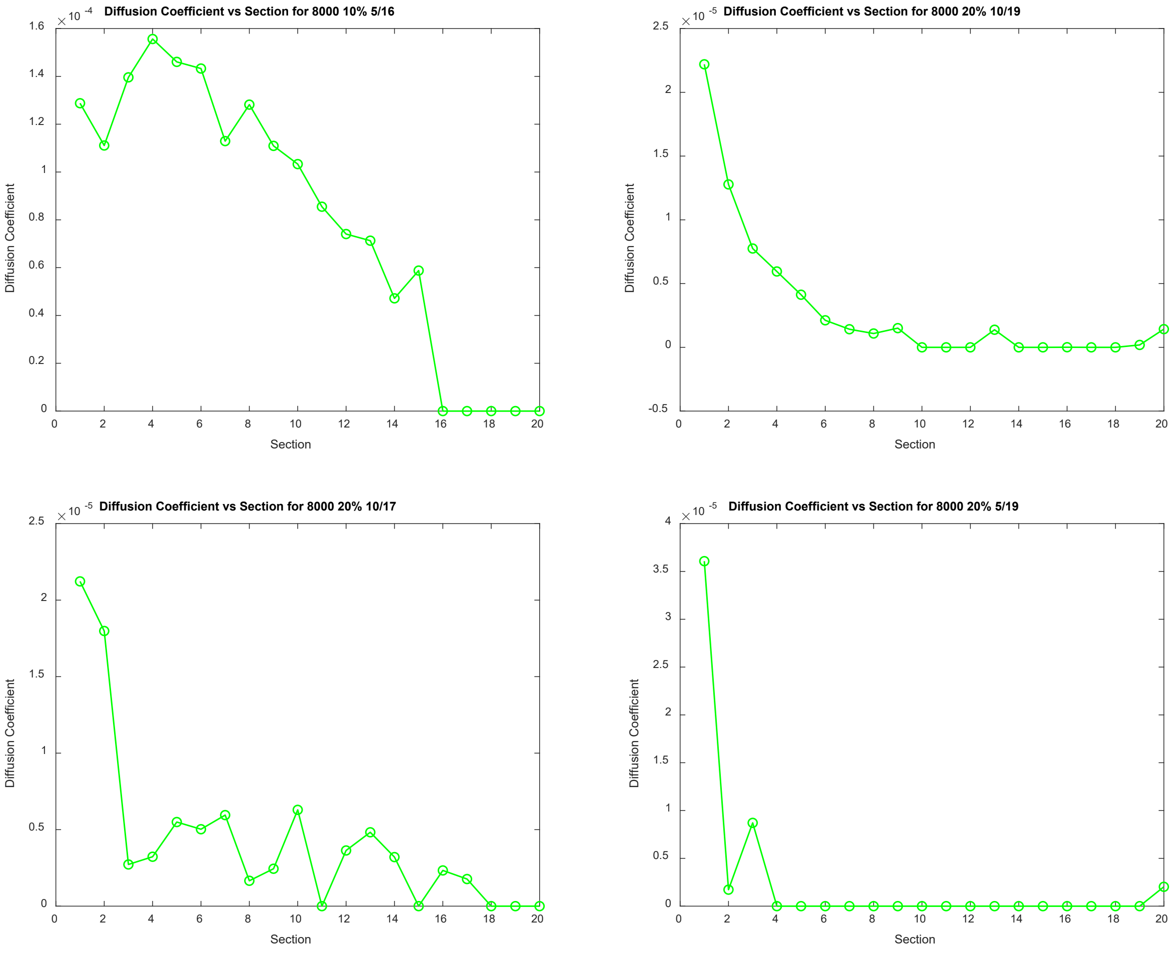

Figure S48. Diffusion coefficient versus section of swelling divided into 20 equal sections for individual experiments on the 8000 g/mol M.W. samples

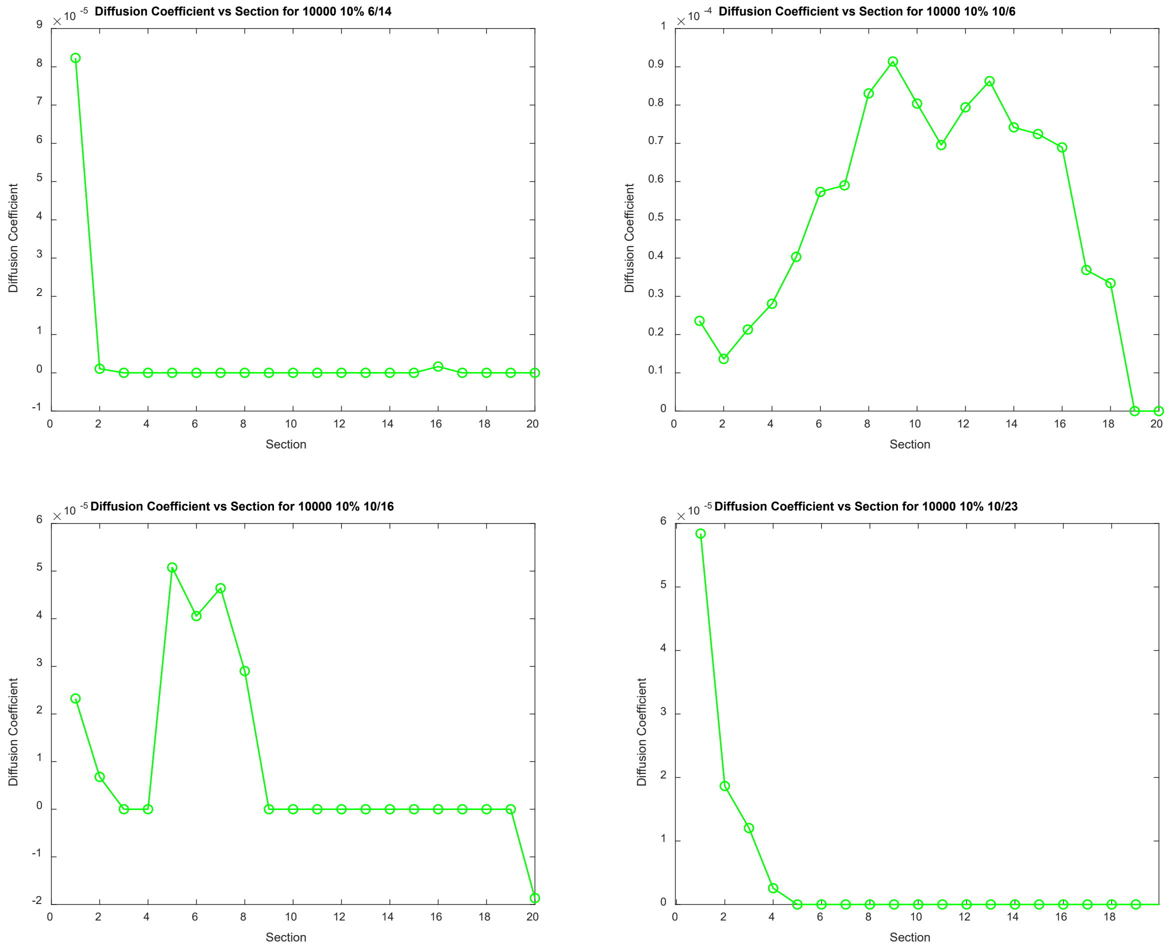

Figure S49. Diffusion coefficient versus section of swelling divided into 20 equal sections for individual experiments on the 10000 g/mol M.W. samples

Table S5. Electrochemical kinetic sorption model fitting parameter results: coefficient, k, and power, n, values of PEGDA 4k Gel swelling at early time, 1000 – 2200 s.

	Experiment		Model			
	$\boldsymbol{n}$	$\boldsymbol{k}\ (\boldsymbol{s^{-n}})$	$\boldsymbol{n}$	$\boldsymbol{k}\ (\boldsymbol{s^{-n}})$	$\boldsymbol{D_{app,Exp}}\ \left(\frac{\boldsymbol{mm^2}}{\boldsymbol{s}}\right)$	$\boldsymbol{D_{app,Model}}\ \left(\frac{\boldsymbol{mm^2}}{\boldsymbol{s}}\right)$
Negative Voltage	2.08	5.74×10^{-9}	0.30644	0.18509	2.58×10^{-12}	4.42×10^{-9}
Neutral	1.29	2.64×10^{-5}	0.29184	0.20346	9.95×10^{-8}	5.34×10^{-9}
Positive Voltage	2.73	1.38×10^{-9}	0.27697	0.22342	1.14×10^{-7}	6.36×10^{-9}

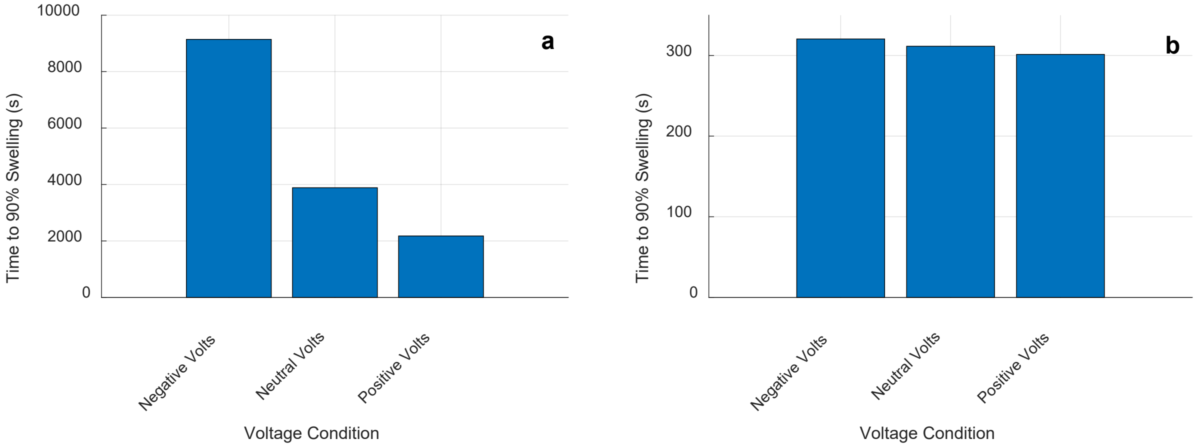

Figure S50. (a) Experimental data showing the time to 90% swelling for PEGDA 4k Gel under negative (-0.35 V), neutral, and positive (+0.35 V) electric fields, in the presence of 0.5 M NaCl in water. Significant differences in swelling times are observed, with negative volts resulting in a 135.3% increase and positive volts leading to a 44% decrease relative to neutral conditions. (b) Theoretical model predictions for time to 90% swelling under negative (-0.35 V), neutral, and positive (+0.35 V) electric fields. The model shows minimal variation with a 2.9% increase for negative volts and a 3.3% decrease for positive volts relative to neutral conditions.

References

(1) Silverstein, R. M.; Webster, F. X.; Kiemle, D. J.; Bryce, D. L. *Spectrometric Identification of Organic Compounds*; Wiley, 2015.
(2) Crank, J. *The Mathematics of Diffusion*; Oxford university press, 1979.
(3) George, K. A.; Wentrup-Byrne, E.; Hill, D. J. T.; Whittaker, A. K. Investigation into the Diffusion of Water into HEMA-co-MOEP Hydrogels. *Biomacromolecules* **2004**, *5* (4), 1194-1199. DOI: 10.1021/bm034477p.
(4) Peppas, N. A. *Hydrogels in Medicine and Pharmacy*; CRC press Boca Raton, FL, 1986.
(5) Qiu, Y.; Park, K. Environment-sensitive Hydrogels for Drug Delivery. *Advanced Drug Delivery Reviews* **2001**, *53* (3), 321-339. DOI: https://doi.org/10.1016/S0169-409X(01)00203-4.
